# Innovative Amino-Functionalization of Pyrido[2,3-*d*]pyrimidine Scaffolds for Broad Therapeutic Applications Supported by Computational Analyses

**DOI:** 10.3390/ph18101472

**Published:** 2025-09-30

**Authors:** Hagar S. El-Hema, Haitham E. Shehata, Mohamed A. Hawata, Eman S. Nossier, Ahmed F. El-Sayed, Najla A. Altwaijry, Asmaa Saleh, Modather F. Hussein, Amr Sabry, Adel A.-H. Abdel-Rahman

**Affiliations:** 1 Basic Science Department (Chemistry), Thebes Higher Institute for Engineering, Thebes Academy, Maadi 11434, Egypt; 2 Chemistry Department, Faculty of Science, Menoufia University, Shebin El-Kom 32511, Egypt; hythamshehata@science.menofia.edu.eg (H.E.S.); adelnassar63@yahoo.com (A.A.-H.A.-R.); 3 Pharmaceutical Medicinal Chemistry and Drug Design Department, Faculty of Pharmacy (Girls), Al-Azhar University, Cairo 11754, Egypt; dr.emannossier@gmail.com; 4 The National Committee of Drugs, Academy of Scientific Research and Technology, Cairo 11516, Egypt; 5 Microbial Genetics Department, Biotechnology Research Institute, National Research Centre, Giza 12622, Egypt; ahmedfikry.nrc@gmail.com; 6 Egypt Center for Research and Regenerative Medicine (ECRRM), Cairo 11517, Egypt; 7 Department of Pharmaceutical Sciences, College of Pharmacy, Princess Nourah Bint Abdulrahman University, P.O. Box 84428, Riyadh 11671, Saudi Arabia; naaltwaijry@pnu.edu.sa (N.A.A.); asali@pnu.edu.sa (A.S.); 8 Chemistry Department, College of Science, Jouf University, P.O. Box 2014, Sakaka, Aljouf 72341, Saudi Arabia; mfhussin@ju.edu.sa; 9 Department of Pharmaceutical Manufacturing, College of Pharmaceutical Sciences and Drug Manufacturing, Misr University for Science and Technology (MUST), 6th of October City, P.O. Box 77, Giza 12591, Egypt; 79365@must.edu.eg

**Keywords:** pyridopyrimidine derivatives, EGFR inhibition, apoptosis, wound healing assay, antibacterial activity, antiviral prediction, molecular docking, molecular dynamics, DFT

## Abstract

**Background:** Derivatives of Pyrido[2,3-*d*]pyrimidine-6-carboxylate are promising multi-target scaffolds. This study focused on synthesizing 16 amino-functionalized derivatives and evaluating their dual anticancer and antibacterial activities, supported by mechanistic and computational analyses. **Objectives:** Design and synthesize derivatives, evaluate cytotoxicity against HeLa, HepG-2, and MCF-7 (selectivity against WI-38), investigate EGFR^WT^ and EGFR^T790M^ inhibition, assess cell cycle, apoptosis, and migration effects, antibacterial efficacy against *E. coli* and *P. aeruginosa*, and perform in silico ADMET, docking, molecular dynamics, DFT, and antiviral predictions. **Methods:** Synthesized 16 derivatives; tested for cytotoxicity, EGFR inhibition, cell cycle, apoptosis, migration; assessed antibacterial activity; performed ADMET profiling, molecular docking, molecular dynamics, and DFT calculations. **Results:** Derivatives **1**, **2**, and **7** showed highest cytotoxicity (IC_50_ = 3.98–17.52 μM; WI-38 IC_50_ = 64.07–81.65 μM). Compound **1** potently inhibited EGFRWT (IC_50_ = 0.093 μM) and EGFRT790M (IC_50_ = 0.174 μM), induced G0/G1 arrest (74.86%) and apoptosis (26.37%), and reduced MCF-7 migration (69.63%). Moderate antibacterial activity observed (MIC = 50 μg/mL). ADMET indicated favorable pharmacokinetics, low CYP inhibition, negative mutagenicity, and oral toxicity class III. Molecular dynamics confirmed stable binding (EGFRWT RMSD 3 Å; EGFRT790M 3.5–4.6 Å) with persistent hydrogen bonds. In silico antiviral evaluation suggested strong binding to HCV NS5A (–9.36 kcal/mol), SARS-CoV-2 Mpro (–9.82 kcal/mol), and *E.coli* DNA gyrase (–10.25 kcal/mol). **Conclusions:** Compound **1** exhibits dual anticancer and antibacterial activity, supported by mechanistic and computational analyses, highlighting pyrido[2,3-*d*]pyrimidines as promising multi-target therapeutic scaffolds.

## 1. Introduction

One of the major challenges to worldwide health is posed by cancer, which ranks second in terms of causes of death behind heart disease. Approximately 10 million people died from cancer worldwide, while 19.3 million new cases of cancer diagnoses were reported in 2023 alone, according to data from the World Health Organization and GLOBOCAN [1,2,3]. Current anticancer medications have serious drawbacks, including high toxicity, poor selectivity, drug resistance, and low absorption, despite recent advances in early diagnosis and chemotherapy [4,5,6]. These issues highlight the ongoing need for innovative therapies that focus on particular genetic factors which are involved in the genesis of cancer.

A central focus of targeted cancer therapy is EGFR, an ErbB family receptor tyrosine kinase widely recognized for its clinical relevance. Alterations in EGFR, either through gene amplification or mutation, are frequently observed in several malignancies, most notably in non-small-cell lung carcinoma (NSCLC), breast cancer, and colorectal cancer [7,8,9,10,11,12]. The clinical effectiveness of agents designed to inhibit EGFR like gefitinib and erlotinib **I** [13], is significantly hindered by resistance-conferring mutations like L858R and T790M, which alter kinase domain conformation and reduce drug binding affinity. Therefore, research has shifted toward 2nd- and 3rd-generation inhibitors, such as, afatinib (2nd) and Osimertinib (3rd), effective against both wild-type and mutant EGFR isoforms. For completeness, it is important to acknowledge the EGFR 4th generation emergence inhibitors like BAY2927088, which were created especially to get beyond the reluctance of earlier generations, including the T790M and C797S mutations. These novel agents demonstrate improved selectivity and potency against resistant EGFR variants, highlighting the ongoing evolution of targeted therapies and the need for new scaffolds capable of dual or multi-target activity [14,15,16,17].

**Figure 1:**
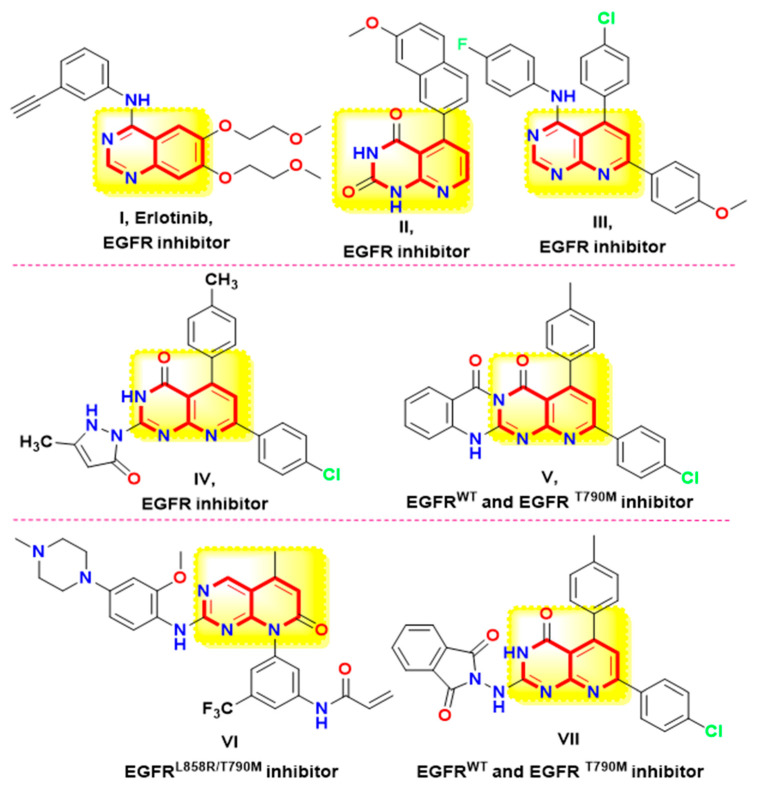
Anticancer agents consist of erlotinib as a reference **I** and other pyrido[2,3-*d*] pyrimidines **II**–**VII** that inhibit EGFR and its mutations.

Heterocyclic scaffolds, notably pyrido[2,3-*d*]pyrimidines, have attracted attention due to their structural closeness to purines and varied biological characteristics [18,19,20,21,22,23,24,25,26]. These frameworks demonstrate a variety of actions, including anticancer and kinase inhibition, as well as antibacterial, antileishmanial, antitubercular, and anti-inflammatory properties [21,27,28,29,30,31,32,33,34,35,36,37,38,39,40,41,42,43,44,45,46,47,48,49,50,51,52]. Several derivatives of pyrido[2,3-*d*]pyrimidine have shown promising EGFR inhibitory effects **II**–**IV**, as well as activity against resistant mutations **VI**–**VII** [20,53,54,55,56,57] (Figure 1), making them good candidates for developing next-generation inhibitors effective against mutant EGFR in cancer drug development [58,59,60,61].

Cancer patients are frequently prone to opportunistic bacterial and viral infections caused by chemotherapy-induced immunosuppression. This co-infection scenario, particularly when antibiotic resistance is prevalent, creates significant therapeutic problems. Designing compounds with anticancer and antibacterial properties against therapeutically important pathogens including *E. coli*, *S. aureus*, *E. faecalis*, and *P. aeruginosa* is a valuable and novel technique [62,63,64,65,66,67].

**Figure 2:**
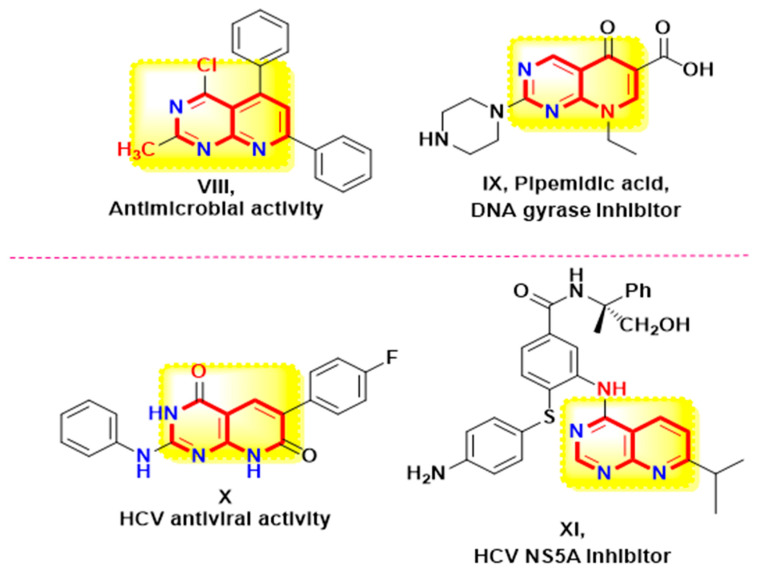
Pyrido[2,3-*d*]pyrimidine-based analogues **VIII**–**XI** bearing antimicrobial and antiviral activity.

According to Elsaedany et al. [68], 4-Chloro-5,7-diphenyl-2-methylpyrido[2,3-*d*]pyrimidine **VIII** shown good antibacterial efficacy against tested pathogens, particularly against Staphylococcus aureus, where it was twice as powerful as the reference. Pipemidic acid **IX** inhibits DNA Gyrase, making it useful for treating bacterial infections, enteritis, diarrhea, and urinary tract infections [69]. Camarasa et al. discovered that 6-(4-fluorophenyl)-2-(phenylamino)pyrido[2,3-*d*]pyrimidine-4,7-dione **X** had most potent anti-HCV (Hepatitis C virus) activity profile among all the derivatives of pyrido[2,3-*d*]pyrimidine tested [70]. Following testing of pyrido[2,3-*d*]pyrimidine-based HCV NS5A inhibitors, derivative **XI** was selected to be administered to a chimpanzee afflicted with HCV, and it demonstrated antiviral activity in vivo [71] (Figure 2).

**Figure 3:**
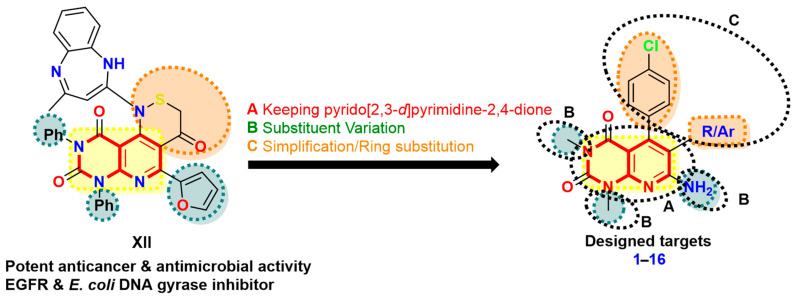
The rational design of newly synthesized 2,4-dioxopyrido[2,3-*d*]pyrimidines **1**–**16** with expected anticancer and antimicrobial activities through inhibition of EGFR and DNA gyrase, respectively.

Based on these findings and previous lead **XII** [72] (Figure 3), which has dual anticancer and antimicrobial activities, 2,4-dioxopyrido[2,3-*d*]pyrimidines series designed using different drug design strategies, such as keeping the main scaffold (2,4-dioxopyrido[2,3-*d*]pyrimidine), variation in substituent at positions 1, 3, and 7, and simplification and/or ring substitution at positions 5 and 6. These derivatives were produced by reacting the parent scaffold with different amines and assessed for cytotoxicity against the MCF-7, HCT-116, and HepG-2 cancer cell lines. Next, derivatives which were assessed as the most potent, in comparison to the EGFR wild-type and mutant isoforms (L858R and T790M). Their effects on the advancement of the MCF-7 cell cycle, apoptosis, and antibacterial activity was tested against *E. coli* (ATCC 25915), *S. aureus* (ATCC 25923), *E. faecalis* (ATCC 29212), and *P. aeruginosa* (ATCC 10145) were investigated and Finally, in silico molecular docking, ADMET, and DFT experiments validated the biological findings and provided guidance for future optimization.

To be thorough, it is necessary to mention the emergence of fourth-generation EGFR inhibitors, which have been particularly created to address resistance associated with prior generations, including the T790M and C797S mutations. These novel medicines show increased selectivity and effectiveness against resistant EGFR variants, emphasizing the continual evolution of targeted treatments and the need for new scaffolds with dual or multi-target action.

## 2. Results and Discussion

### 2.1. Chemical Part

**Scheme 1:**
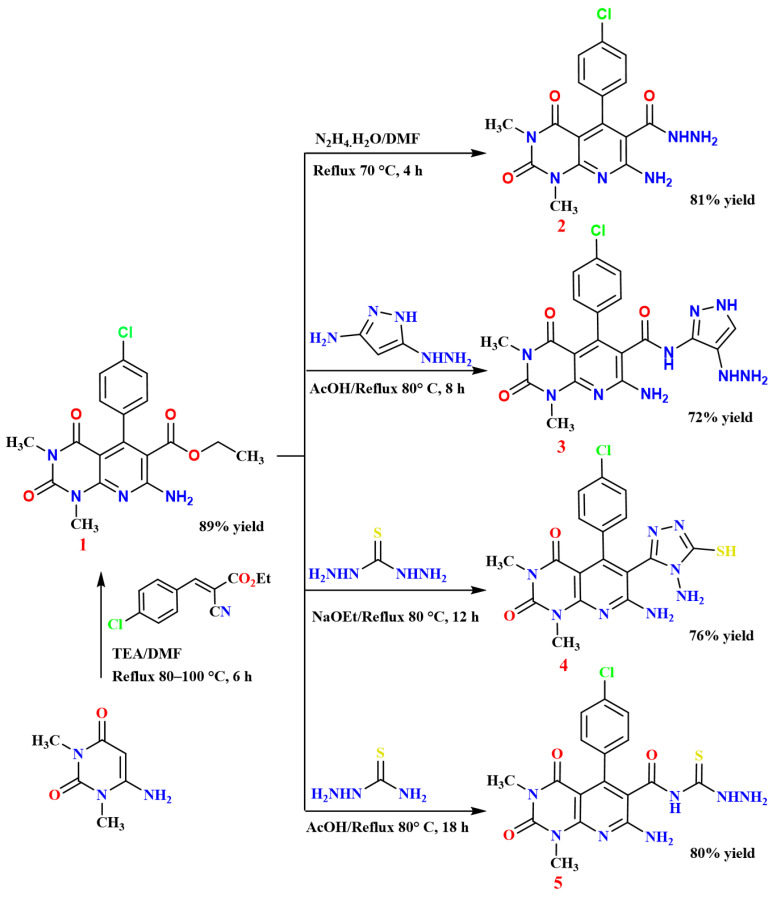
Synthesis of Pyridopyrimidine, acid hydrazide, hydrazinyl pyrazole, amino-triazole-3-thiol, and thio hydrazide derivatives **1**–**5**.

**Scheme 2:**
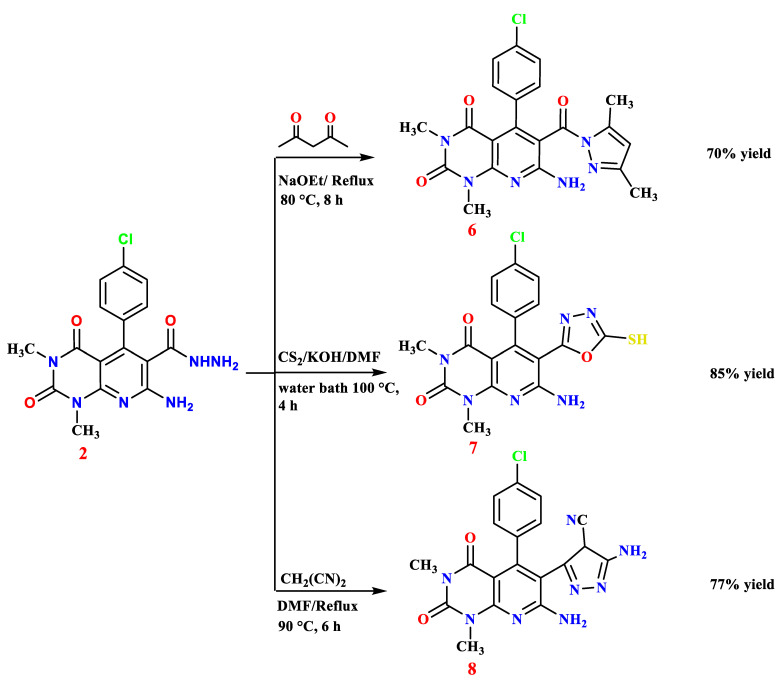
Synthesis of dimethyl-pyrazole, oxadiazole-2-thiol, and amino-pyrazole-4-carbonitrile derivatives **6**–**8**.

**Scheme 3:**
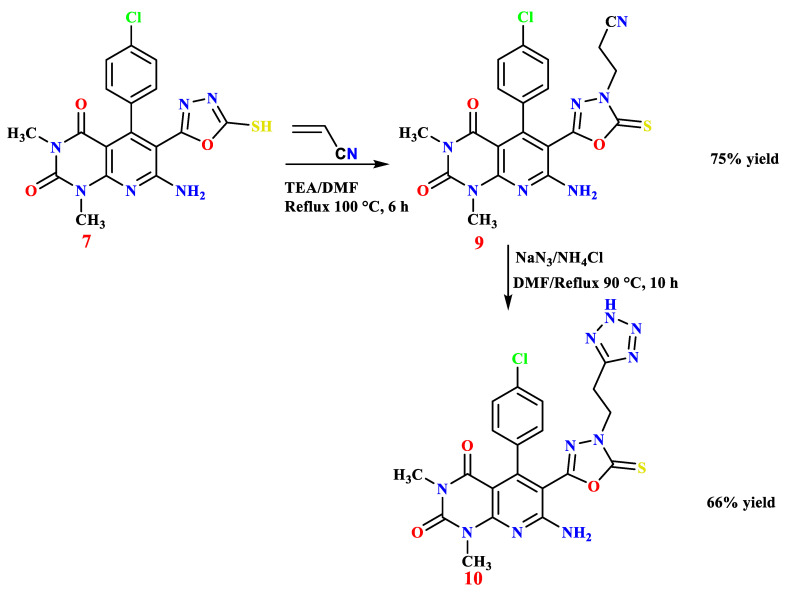
Synthesis of thioxo-oxadiazol-propanenitrile and ethyl tetrazole derivatives **9** and **10**.

**Scheme 4:**
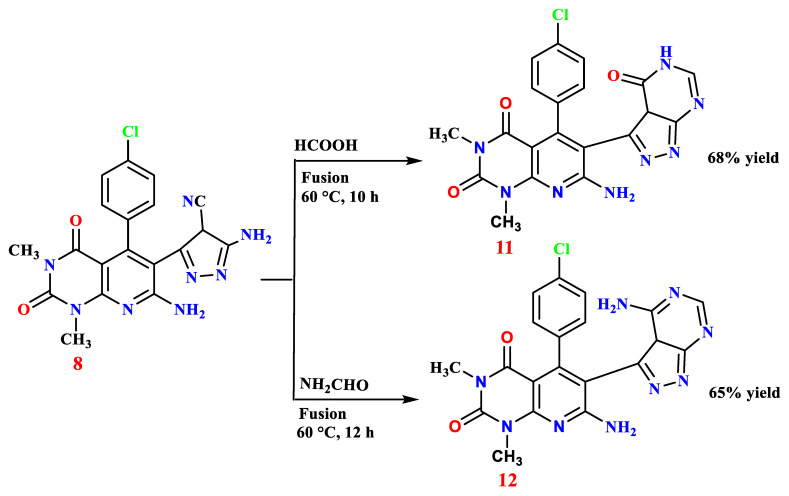
Synthesis of fused -pyrazolopyrimidin-4-one and pyrazolopyrimidin-4-amine derivatives **11** and **12**.

**Scheme 5:**
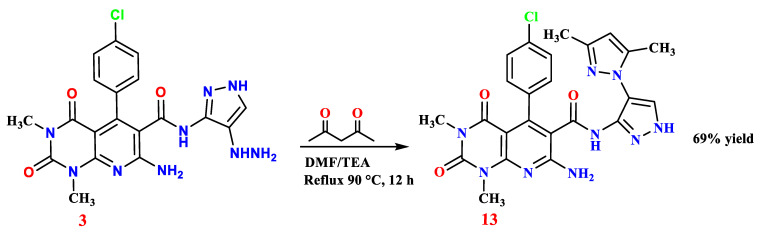
Synthesis of dimethyl pyrazole derivatives **13**.

**Scheme 6:**
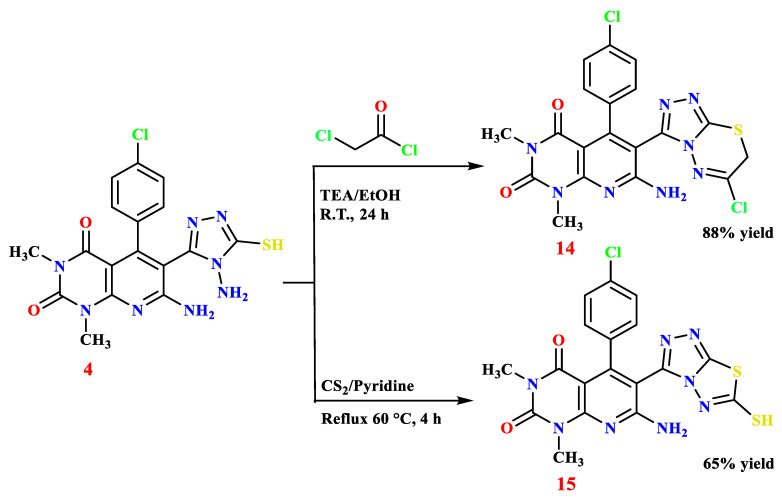
Chloro triazolothiadiazine and triazolothiadiazole-6-thiol derivatives **14** and **15** synthesis.

**Scheme 7:**
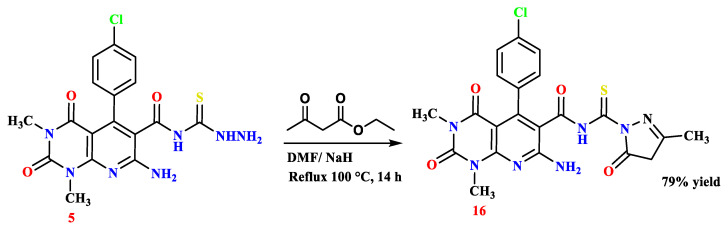
Synthesis of methyl-pyrazol-3-one derivative **16**.

Scheme 1, Scheme 2, Scheme 3, Scheme 4, Scheme 5, Scheme 6 and Scheme 7 outline the synthesis of targets **1**–**16**. Scheme 1 shows the stepwise preparation of pyridopyrimidine analogues **1**–**5**.

The key precursor, 6-amino-1,3-dimethyluracil [73], reacted with ethyl (*E*)-3-(4-chlorophenyl)-2-cyanoacrylate in anhydrous DMF/Et_3_N to afford compound **1** (ethyl amino tetrahydropyridopyrimidine-6-carboxylate). Compound **1** was subsequently treated with hydrazine hydrate, 5-hydrazinyl-1*H*-pyrazol-3-amine, thiocarbohydrazide, and thiosemicarbazide, producing derivatives **2**–**5**.

Spectral analysis confirmed the structures. Compound **1** was fully characterized (IR, ^1^H NMR, ^13^C NMR), representing its first report. Compound **2** showed ester–carbohydrazide conversion: IR (ν NH_2_ at 3182 cm^−1^; ν C=O shift to 1625 cm^−1^), ^1^H NMR (ethyl signals lost, new NH_2_/NH at 6.24 and 9.10 ppm), and ^13^C NMR (amide carbonyl 162.87 ppm; ester signals absent).

Compound **3** confirmed ester–amide conversion and pyrazole formation: IR (ν NH_2_/NH at 3348, 3202 cm^−1^; C=N at 1517 cm^−1^), ^1^H NMR (D_2_O-exchangeable NH_2_ at 4.43 ppm; pyrazole CH at 7.82 ppm), ^13^C NMR (C–NHNH_2_ at 118.58 ppm, pyrazole CH 127.67 ppm, C=N 142.98 ppm).

Compound **4** showed incorporation of pyrazole and thiol groups with ester removal: IR (ν NH_2_ at 3354, 3204 cm^−1^; ν SH at 2346 cm^−1^; C=N at 1551, 1507 cm^−1^), ^1^H NMR (NH_2_ at 5.09 ppm; SH at 13.45 ppm), ^13^C NMR (pyrazole–pyridine C at 107.35 ppm; triazole C=N at 152.04, 169.00 ppm), MS (*m*/*z* 430.63 [M^+^], base peak 186).

Compound **5** confirmed thiosemicarbazone formation: IR (ν NH_2_/NH at 3349, 3254 cm^−1^; ν C=O at 1597 cm^−1^; ν C=S at 1090 cm^−1^), ^1^H NMR (exchangeable NH_2_/NH at 3.01, 10.89, 12.06 ppm; ethyl signals absent), ^13^C NMR (C=S carbonyl 186.05 ppm; amide carbonyl 171.62 ppm).

Scheme 2 depicts the synthesis of pyrazole and oxadiazole derivatives **6**–**8** from Compound **2**. Reaction with acetylacetone in ethanolic sodium ethoxide yielded dimethyl-substituted pyrazole **6**. Mercapto-oxadiazole **7** was obtained by treating Compound **2** with carbon disulfide under basic conditions, while cyclized derivative **8** was formed via reflux with malononitrile. All structures were confirmed by spectral analysis.

Spectral data confirmed the structures of Compounds **6**–**8**. For **6**, IR showed ν CH_3_ at 2819/2736 cm^−1^, ν C=O shifted 1625 → 1740 cm^−1^, and ν C=N at 1530 cm^−1^, with NH_2_/NH bands absent. ^1^H NMR displayed CH_3_ singlets at 2.71/2.87 ppm and pyrazole CH at 6.24 ppm; ^13^C NMR showed CH_3_ at 13.04/13.97 ppm, pyrazole CH at 110.02 ppm, C–CH_3_ at 141.64 ppm, and C=N at 151.06 ppm. MS gave *m*/*z* 438.87 [M^+^], base peak 132.

Compound **7**’s mercapto-oxadiazole formation was confirmed by IR (ν C=N 1599/1588 cm^−1^, ν SH 2596 cm^−1^; NH_2_/NH/amide absent), ^1^H NMR SH singlet at 13.80 ppm, and ^13^C NMR pyridine–oxadiazole at 107.77 ppm, C=N at 166.66/169.59 ppm; amide carbonyl disappeared.

For **8**, IR showed NH_2_ at 3416/3216 cm^−1^, cyano at 2222 cm^−1^, pyrazole C=N at 1555/1517 cm^−1^, with NH and amide C=O absent. ^1^H NMR displayed pyrazole CH at 2.79 ppm and NH_2_ at 6.23 ppm (D_2_O exchangeable); ^13^C NMR showed pyrazole C at 33.50 ppm, pyridine C at 100.24 ppm, CN at 118.03 ppm, and C=N at 165.80/186.90 ppm; amide carbonyl at 166.61 ppm disappeared.

In Scheme 3, the oxadiazol propanenitrile derivative **9** was prepared by refluxing compound **7** with acrylonitrile in a basic media, followed by treatment with sodium azid and ammonium hydrochloride to produce tetrazole derivative **10**.

Spectral data confirmed the structures of Compounds **9** and **10**. For **9**, IR showed CH_2_ stretches at 2989/2873 cm^−1^, ν C≡N at 2249 cm^−1^, C=S at 1058 cm^−1^, and disappearance of ν SH (2596 cm^−1^). ^1^H NMR displayed acrylonitrile CH_2_ triplets at 3.91/4.15 ppm, with SH proton absent at 13.80 ppm. ^13^C NMR showed CH_2_ at 16.10/45.70 ppm, CN at 118.03 ppm, and C=S at 174.00 ppm, while the previous oxadiazole C=N at 169.59 ppm disappeared.

Compound **10** formation of the tetrazole ring was confirmed by IR (ν NH 3282 cm^−1^, ν C=N 1491 cm^−1^, ν N=N 1297 cm^−1^; disappearance of ν C≡N 2249 cm^−1^), ^1^H NMR (CH_2_ shifted 3.91 → 2.71 ppm; D_2_O-exchangeable NH singlet at 14.00 ppm), and ^13^C NMR (CH_2_ at 22.00/54.89 ppm, C=N at 165.94 ppm; CN at 118.03 ppm absent). MS showed *m*/*z* 512.93 [M^+^], base peak 80.

Scheme 4 depicts the planned molecular method for synthesizing the novel fused pyrazole pyrimidinone and amino pyrimidine derivatives **11** and **12**.

Compound **8** was fused with formic acid and formamide under sand bath heating to yield 5,6-dihydropyrimidin-4(3*H*)-one (**11**) and 5,6-dihydropyrimidin-4-amine (**12**).

For **11**, IR showed ν NH 3161 cm^−1^, ν CH 2870 cm^−1^, lactam C=O 1723 cm^−1^, and pyrimidinone C=N 1635 cm^−1^, with disappearance of NH_2_ and cyano bands (3416, 3216, 2222 cm^−1^). ^1^H NMR displayed pyrazole CH at 2.32 ppm, pyrimidinone CH at 8.38 ppm, and NH at 11.44 ppm (D_2_O-exchangeable). ^13^C NMR showed pyrazole CH 46.83 ppm, pyrimidinone C=N 150.98 ppm, lactam C=O 169.85 ppm, pyrazole C=N 181.01 ppm; cyano carbon at 118.03 ppm disappeared. MS gave *m*/*z* 450.84 [M^+^], base peak 319.

For **12**, IR revealed NH_2_ at 3422 cm^−1^ and pyrimidin-4-amine C=N at 1610/1594 cm^−1^; cyano band at 2222 cm^−1^ disappeared. ^1^H NMR showed pyrazole CH at 1.65 ppm, D_2_O-exchangeable NH_2_ at 8.25 ppm, and pyrimidine CH at 8.91 ppm. ^13^C NMR displayed pyrazole CH 35.86 ppm, pyrimidine C=N 162.42/163.09 ppm, pyrazole C=N 167.12/178.02 ppm; cyano carbon absent, confirming formation.

The dimethyl pyrazole derivative **13** was effectively produced by refluxing compound **3** with acetylacetone in a basic solution while employing triethylamine as a catalyst (Scheme 5).

Spectral data confirmed Compound **13** as a dimethyl-substituted pyrazole. IR showed ν C=N at 1491 cm^−1^. ^1^H NMR displayed CH_3_ singlets at 1.89/2.10 ppm and pyrazole CH at 5.04 ppm; no D_2_O-exchangeable NH_2_/NH signals were observed. ^13^C NMR showed CH_3_ at 12.32/13.50 ppm, pyrazole C–CH_3_ 90.00 ppm, CH 112.25 ppm, C=N 142.20 ppm, and quaternary C 155.35 ppm. MS gave *m*/*z* 519 [M^+^], base peak 300.

In Scheme 6, product **4** was exposed to further reactions with 2-chloroacetyl chloride and carbon disulfide, yielding the corresponding derivatives: 5-chloro-thiadiazine (compound **14**) and 1,3-thiadiazole-2-thiol (compound **15**).

Spectral data confirmed Compounds **14** and **15**. For **14**, IR showed ν C=N at 1491 cm^−1^ and disappearance of ν SH (2346 cm^−1^), ^1^H NMR displayed CH_2_ singlet at 4.92 ppm with SH absent, and ^13^C NMR showed CH_2_ at 34.16 ppm, C=N at 151.06 ppm, and imine triazole C shifted 169.00 → 162.80 ppm.

Compound **15**’s 1,3-thiadiazole-2-thiol formation was confirmed by IR (ν SH 2550 cm^−1^), ^1^H NMR (SH singlet at 13.24 ppm), ^13^C NMR (imine C 188.17 ppm), and MS *m*/*z* 572 [M^+^], base peak 284.

Finally, Scheme 7 produced 5-methyl pyrazolone derivative **16** by refluxing compound **5** with ethyl acetoacetate in a strongly basic solution containing sodium hydride as the base.

Spectral data confirmed Compound **16** as a 5-methyl pyrazolone. IR showed ν CH 2839/2816 cm^−1^, C=O 1727 cm^−1^, and C=N 1516 cm^−1^. ^1^H NMR displayed CH_3_ singlet at 1.94 ppm and CH_2_ at 3.01 ppm. ^13^C NMR showed CH_3_ 16.00 ppm, CH_2_ 45.05 ppm, C=N 159.58 ppm, C=O 165.33 ppm, and C=S shifted 186.05 → 182.25 ppm. MS gave *m*/*z* 499 [M^+^], base peak 245.

### 2.2. Biological Evaluation

#### 2.2.1. Antiproliferative In Vitro Potency

**Figure 4:**
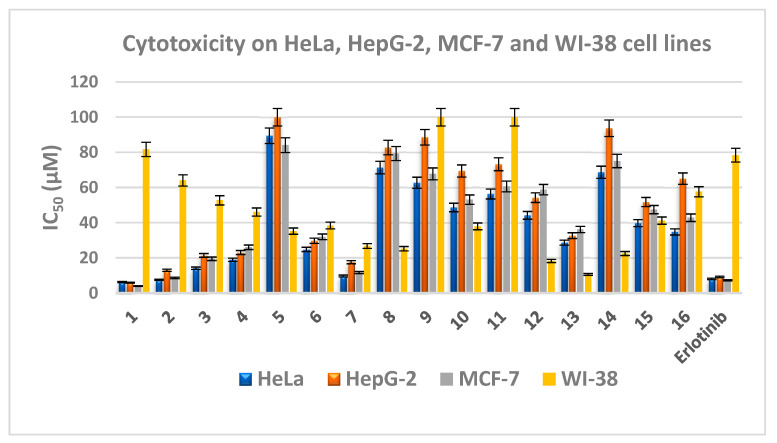
The cytotoxic effects of 1,3-dimethyl-2,4-dioxopyrido[2,3-*d*]pyrimidines **1**–**16** on WI-38 against HeLa, HepG-2, and MCF-7 cell lines that was tested at multiple doses employing the MTT assay, represented by IC_50_ values.

Cytotoxicity testing of 1,3-dimethyl-2,4-dioxopyrido[2,3-*d*]pyrimidine derivatives (**1**–**16**) on HeLa, HepG-2, and MCF-7 cells via MTT assay [74,75,76] showed variable micromolar IC_50_ values, comparable to erlotinib. (Figure 4, Table S2).

Compounds **1**, **2**, and **7** were the most active in the series, their IC_50_ values varying from 3.98 to 17.52 µM across all examined cell lines. Compound **1** inhibited MCF-7 cells the greatest (IC_50_ = 3.98 ± 0.2 µM), outperforming erlotinib (IC_50_ = 7.26 ± 0.3 µM) in this line. It also had considerable activity against HeLa and HepG-2 cells. Furthermore, compound **7** showed significant cytotoxicity, especially against HeLa (IC_50_ = 9.72 ± 0.9 µM). In contrast, derivatives **5**, **8**, **9**, **10**, **11**, and **14** showed modest action, with IC_50_ values >50 µM in most cases, indicating lower affinity for the molecular target(s) or poor cell permeability.

In addition, all derivatives were tested in vitro towards normal lung fibroblast WI-38 cell lines to determine their safety. The ideal IC50 values for potential derivatives **1** and **2** are 81.65 ± 4.1 and 64.07 ± 3.5 µM, respectively, while erlotinib has an IC50 of 78.32 ± 3.9 µM. Consequently, they may be classified as anticancer drugs with acceptable safety. A chemical’s selectivity and capacity to target cancer cells without endangering healthy cells are gauged by its SI value (WI-38); the greater the value, the safer the substance. The selectivity index (SI) toward tumor cells was measured as the IC_50_ value in WI-38 fibroblasts relative to that in malignant cell lines.

##### Best Compounds Based on SI (Selectivity)

- Compound 1 achieved the highest SI values, making it the most selective among all compounds: 20.51 on MCF-7 cells, 13.83 on HepG-2 cells, and 12.98 on HeLa cells.
- Compound 2 also showed good selectivity, but it was lower than both Compound 1 and Erlotinib. Based on this analysis, Future studies should concentrate on comprehending Compound 1’s mode of action and how it achieves this exceptional efficacy and selectivity.

##### Structure-Activity Relationship Study

The cytotoxic evaluation of 1,3-dimethyl-2,4-dioxopyrido[2,3-*d*]pyrimidine derivatives towards cell lines (HeLa, HepG-2, and MCF-7) demonstrated a clear influence of C-6 substitution type, size, and electronic character on anticancer activity. Compound **1**, ethyl 7-amino-5-(4-chlorophenyl)-1,3-dimethyl-2,4-dioxo-1,2,3,4-tetrahydropyrido[2,3-*d*]pyrimidine-6-carboxylate, containing a C-6 ethyl ester, displayed the highest efficacy (IC_50_: 3.98–6.29 µM), exceeding erlotinib in MCF-7 cells. The ethyl ester’s modest bulk and lipophilicity are anticipated to increase hydrophobic interactions in the binding pocket while retaining high cell permeability.

Replacing the ester with the more polar 6-carbohydrazide in **2** decreased activity (IC_50_: 7.54–12.94 µM). The enhanced polarity and hydrogen-bonding ability may boost solubility while disrupting hydrophobic fit. Adding a bulkier *N*-(4-hydrazinyl-1*H*-pyrazol-3-yl)-6-carboxamide in **3** or 6-(4-amino-5-mercapto-4*H*-1,2,4-triazol-3-yl) in **4** reduced potency (14–26 µM), perhaps due to steric interference with optimum binding and altered electron distribution at the C-6 region.

The *N*-(hydrazinecarbonothioyl)-6-carboxamide in **5** resulted in a significant loss of activity (IC_50_ > 84 µM), probably due to extra steric bulk and high electron density around the thioamide, which may repel electron-rich protein residues or hamper structural fit. The 6-(3,5-dimethyl-1*H*-pyrazole-1-carbonyl) substitution in **6** maintained considerable efficacy (24–32 µM), with the planar heterocycle helping π-π interactions, but was lowered compared to the ester due to higher steric hindrance from the dimethyl groups.

In contrast, 6-(5-mercapto-1,3,4-oxadiazol-2-yl) in **7** exhibited significantly higher potency (IC_50_: 9.72–17.52 µM), comparable to compounds **1** and **2**. The oxadiazole ring has electron-drawing properties and a compact shape, promoting strong interactions with little steric penalty. Adding 3-amino-4*H*-pyrazole-4-carbonitrile in **8** or 2-thioxo-1,3,4-oxadiazol-3(2*H*)-yl propanenitrile in **9** significantly decreased potency (62–88 µM). The nitrile groups are significantly electron-withdrawing, potentially disrupting crucial hydrogen-bond donor-acceptor balances, and the bulky appendages are expected to produce steric conflict. The tetrazolyl-oxadiazole-thione **10** and fused pyrazolo[3,4-*d*]pyrimidine derivatives **11**, **12** had low activity (44–73 µM).

These rigid, bulky complexes are anticipated to limit conformational flexibility in the binding site and modify electron distribution in a way that lowers affinity. The *N*-(3,5-dimethyl-1′*H*-[1,4′-bipyrazol]-3′-yl)-6-carboxamide **13** showed low-moderate potency (28–36 µM), whereas 6-(6-chloro-7*H*-[1,2,4]triazolo [3,4-*b*][1,3,4]thiadiazin-3-yl) **14** was among the weakest (69–94 µM). In the latter, bulk and chlorine substitutions may cause steric and electrical mismatches with the binding site. The efficacy of 6-(6-mercapto-[1,2,4]triazolo [3,4-*b*][1,3,4]thiadiazol-3-yl) **15** increased slightly over **14** (40–52 µM) due to possible extra thiol-mediated interactions, but remained low. *N*-(3-methyl-5-oxo-4,5-dihydro-1*H*-pyrazole-1-carbonothioyl)-6-carboxamide **16** had modest activity (35–65 µM), possibly due to steric and electron-rich sulfur effects that disrupted ideal orientation.

**Figure 5:**
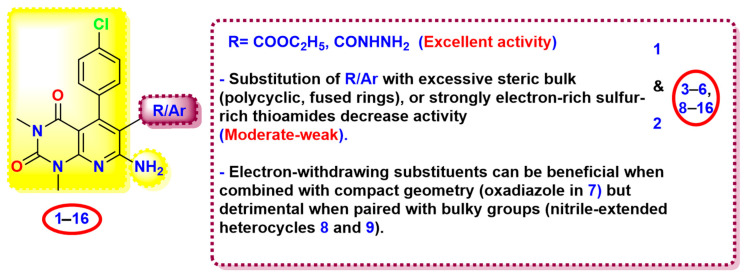
SAR graphic of 1,3-dimethyl-2,4-dioxopyrido[2,3-*d*]pyrimidines **1**–**16** that were identified as possible cytotoxic agents targeting the human malignant cells HeLa, HepG-2, and MCF-7. Ar = aromatic group; R = aliphatic group.

In conclusion, the best activity requires moderate-sized, slightly lipophilic, and electron-balanced groups (e.g., ethyl ester in **1**, carbohydrazide in **2,** and mercapto-oxadiazole in **7**). Loss of activity is associated with high polarity (hydrazide), excessive steric bulk (polycyclic, fused rings), or strongly electron-rich sulfur-rich thioamides. Electron-withdrawing substituents can be beneficial when combined with compact geometry (oxadiazole in **7**) but detrimental when paired with bulky groups (nitrile-extended heterocycles **8** and **9**). Finally, planarity and reduced steric hindrance at C-6 appear essential for maintaining strong cytotoxic activity across cancer cell lines (Figure 5).

#### 2.2.2. In Vitro Inhibitory Activity Evaluation Against Wild EGFR, Mutant EGFR (L858R), and EGFR (T790M)

The synthesized 1,3-dimethyl uracil derivatives **1** and **2** inhibitory potency was tested in vitro against wild-type EGFR, the sensitizing L858R mutant, and the resistance-associated T790M mutant, and compared to the clinically authorized EGFR-TKI erlotinib [77,78] (Table 1).

The IC_50_ of compound **1** was 0.093 ± 0.003 μM towards wild-type EGFR, comparable to erlotinib (0.051 ± 0.002 μM) and superior to compound **2** (0.212 ± 0.007 μM). This high activity indicates that the planar heteroaromatic pyridopyrimidine scaffold effectively engages the ATP-binding pocket, establishing critical hinge area interactions similar to those found in erlotinib.

For the L858R mutant, a clinically important activating mutation that boosts EGFR kinase activity, both drugs retained significant activity although at a lower potency than wild-type EGFR. Compound **1** (1.157 ± 0.036 μM) had a 12-fold lower potency than wild-type EGFR, while compound **2** (1.603 ± 0.050 μM) showed a 7.5-fold drop. Erlotinib (0.239 ± 0.007 μM) resulted in a smaller ~4.7-fold reduction. This lower efficacy in the L858R version could be attributed to small conformational changes within the kinase domain that affect inhibitor accommodation, a tendency shared by other ATP-competitive TKIs.

Compound **1** exhibited substantial activity (0.174 ± 0.006 μM) against the T790M gatekeeper mutant, which causes resistance to first-generation EGFR inhibitors. It was only about 2-fold less powerful than against wild-type EGFR. In comparison, erlotinib (0.098 ± 0.003 μM) exhibited a comparable potency drop, while compound **2** (0.854 ± 0.027 μM) showed a more significant activity loss (~4-fold reduction). The significant T790M activity of compound **1** indicates that the scaffold can partially overcome the steric hindrance created by the methionine substitution, possibly because to favorable hydrophobic and hydrogen bonding interactions within the changed ATP pocket.

#### 2.2.3. 1,3-Dimethyl-2,4-dioxopyrido[2,3-*d*]pyrimidine **1** Cell Cycle Arrest and Apoptosis

Cell cycle and apoptosis assays using flow cytometry and annexin-V staining [79,80] were conducted to explore the mechanism behind the cytotoxic effect of compound **1**, the most active derivative of the tested 1,3-dimethyl-2,4-dioxopyrido [2,3-*d*]pyrimidine series.

**Figure 6:**
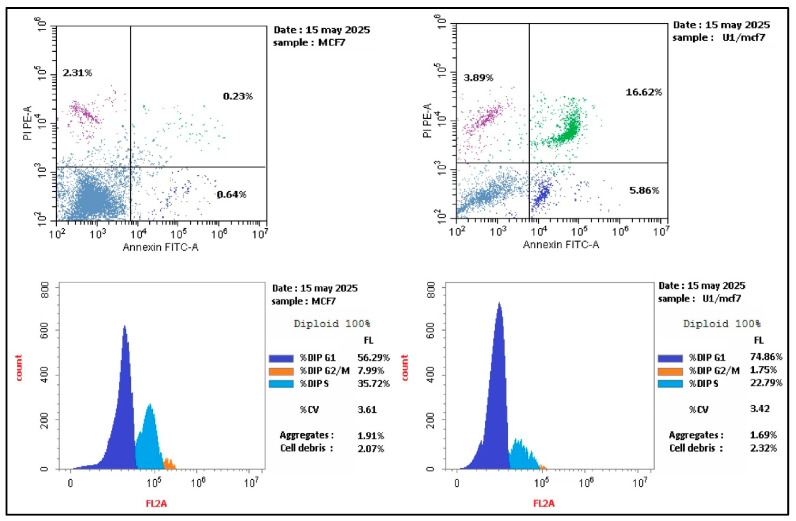
Effect of 1,3-dimethyl-2,4-dioxopyrido[2,3-*d*]pyrimidine derivative (Compound **1**) on the percentage of MCF-7 cells showing V-FITC-positive annexin staining, along with cell cycle analysis.

**Figure 7:**
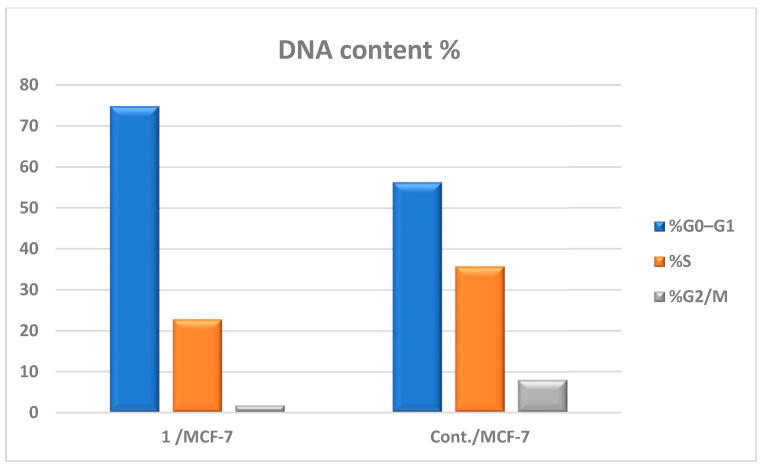
Cell cycle analysis of Compound **1** (1,3-dimethyl-2,4-dioxopyrido[2,3-*d*]pyrimidine derivative).

Flow cytometric analysis after 48 h of treatment with compound **1** (Table S4, Figure 6 and Figure 7) showed that cells of MCF-7 were accumulated significantly in the G0/G1 phase (74.86%) compared to untreated controls (56.29%). Alongside this, the S phase population significantly decreased (22.79% vs. 35.72% in control), and a sharp decline in the G2/M phase fraction (1.75% vs. 7.99% in control). These results suggest that compound **1** triggers G1 phase cell cycle arrest, effectively halting cell cycle progression before DNA synthesis.

**Figure 8:**
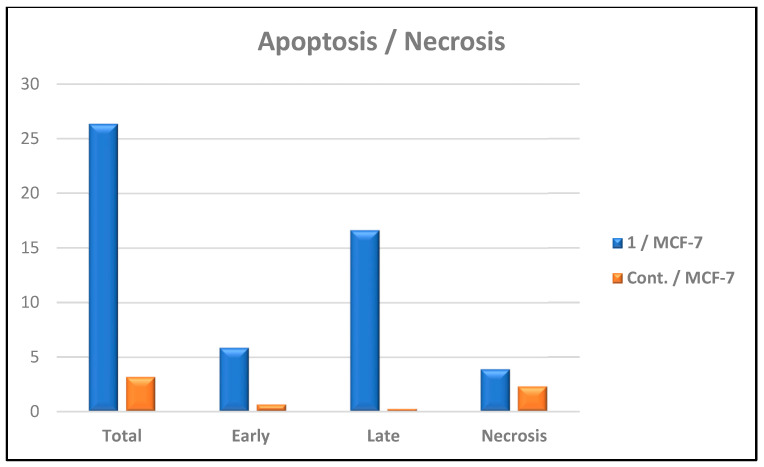
1,3-dimethyl-2,4-dioxopyrido[2,3-*d*]pyrimidine **1**’s impact upon apoptotic activity.

Apoptosis analysis (Table S5, Figure 6 and Figure 8) demonstrated a marked increase in total apoptotic cells upon exposure to compound **1** (26.37%) compared to control cells (3.18%). The apoptotic population comprised both early apoptosis (5.86%) and a predominant late apoptosis fraction (16.62%), with a modest increase in necrosis (3.89% vs. 2.31% in control). The dominance of late apoptosis suggests that compound **1** not only triggers apoptotic signaling but also drives cells to complete programmed cell death within the 48 h treatment period. The significant induction of apoptosis, together with G0/G1 arrest, points to a dual mechanism whereby compound **1** suppresses proliferation and promotes elimination of cancer cells through apoptotic pathways.

#### 2.2.4. Wound Healing Assay

**Figure 9:**
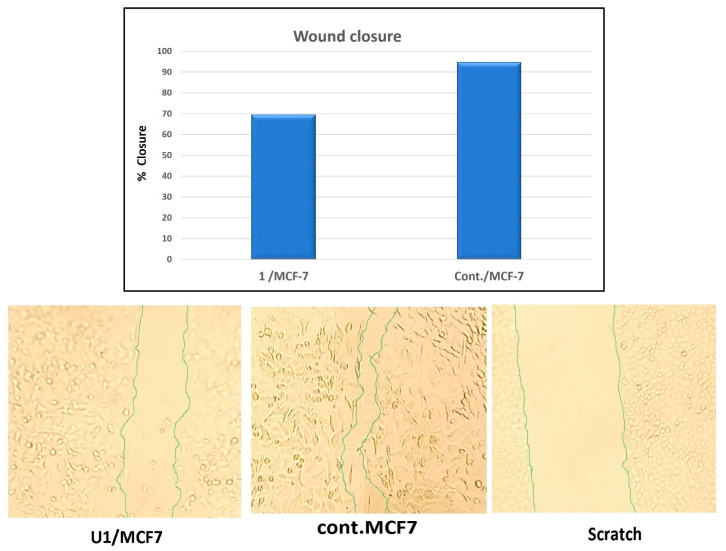
Wound healing assay for MCF-7 cells treated with 1,3-dimethyl-2,4-dioxopyrido[2,3-*d*]pyrimidine **1** compared with control untreated cells.

The preformation of wound healing (scratch) assay to assess the migration ability of MCF-7 cells following treatment with 1,3-dimethyl-2,4-dioxopyrido[2,3-*d*]pyrimidine **1**, compared to untreated controls. After 72 h, the control group exhibited 94.82 ± 3.50% wound closure, indicating near-complete gap filling, while compound **1** treatment achieved 69.63 ± 2.57% closure. This reduction was supported by shorter migration lengths (0.31–0.32 mm vs. 0.42–0.43 mm in controls) and smaller migrated areas (0.564 mm^2^ vs. 0.768 mm^2^). The marked decrease in closure percentage suggests that derivative **1** impairs cellular migration in MCF-7 cells (Figure 9, Table S6).

Overall, the 1,3-dimethyl-2,4-dioxopyrido[2,3-*d*]pyrimidine **1**’s ability to limit wound closure suggests potential as an anti-metastatic agent in breast cancer therapy.

#### 2.2.5. In Vitro Antibacterial Activity

**Figure 10:**
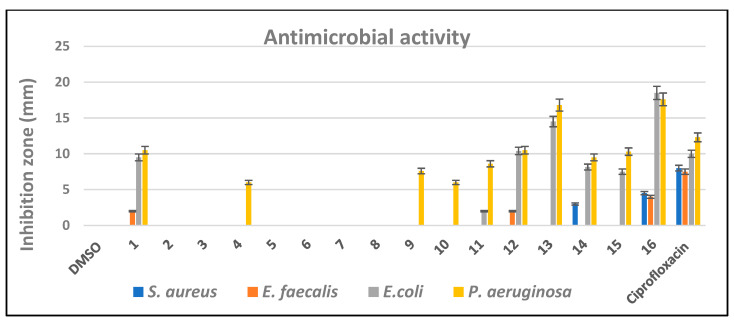
The antibacterial activity of samples towards *S. aureus* ATCC25923, *E. faecalis* ATCC29212, *P. aeruginosa* ATCC10145, and *E. coli* ATCC25915, as determined by the well diffusion method.

Antibacterial activity evaluation was performed using four bacterial strain types: *Staphylococcus aureus* (ATCC25923), *Escherichia coli* (ATCC25915), *Enterococcus faecalis* (ATCC29212)*,* and *Pseudomonas aeruginosa* (ATCC10145) by the well diffusion method [81,82,83,84] (Table S7, Figure 10).

Table 2 presents the values of MIC (µg/mL) for compounds **1**, **12**, **13**, and **16** against four bacterial strains: *E. coli* ATCC25915, *S. aureus* ATCC25923, *E. faecalis* ATCC29212, and *P. aeruginosa* ATCC10145.

Compound **1** has moderate activity, with MICs of 50 µg/mL against *E. coli* and *P. aeruginosa* and 100 µg/mL against *S. aureus* and *E. faecalis*, demonstrating balanced efficacy across strains. Compound **12** has similar action against *E. coli* and *P. aeruginosa* (50 µg/mL), but less effective against *S. aureus* (>100 µg/mL) and *E. faecalis* (100 µg/mL), indicating selective potency.

Compound **13** consistently inhibits *E. coli* at 50 µg/mL but shows reduced effectiveness against *S. aureus* (>100 µg/mL), E. faecalis (>100 µg/mL), and *P. aeruginosa* (100 µg/mL), hinting at lower activity against Gram-positive bacteria. Similarly, Compound **16** matches the 50 µg/mL MIC against *E. coli* and *P. aeruginosa*, with moderate efficacy at 100 µg/mL against *S. aureus* and *E. faecalis*. Overall, the compounds demonstrate the lowest MICs against *E. coli* and *P. aeruginosa*, indicating better activity against these Gram-negative bacteria, while *S. aureus* appears least susceptible with MICs ≥ 100 µg/mL [85].

Although the antibacterial activity was moderate compared to specialized standard antibiotics ciprofloxacin, the demonstrated effect against resilient Gram-negative pathogens highlights the scaffold pyrido[2,3-*d*]pyrimidine potential for further development as a multi-target therapeutic agent.

### 2.3. Computational Studies

#### 2.3.1. ADMET In Silico Prediction

**Figure 11:**
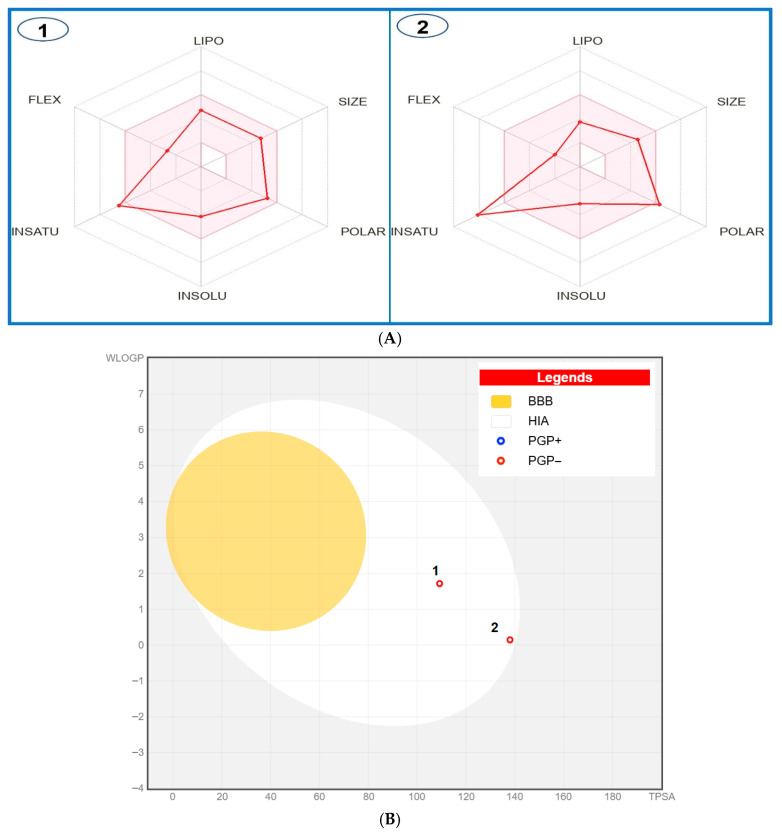
(**A**) Bioavailability radar plots of compounds **1** and **2** showing predicted (red) and optimal (pink) values. (**B**) Boiled egg diagram illustrating gastrointestinal absorption, blood–brain barrier penetration, and P-glycoprotein substrate status (GP− = non-substrate, GP+ = substrate).

ADMET computational profiling by free online resources SwissADME and AdmetSAR 1.0 [86,87,88] demonstrated that 1,3-dimethyl-2,4-dioxopyrido[2,3-*d*]pyrimidines **1** and **2** satisfy Lipinski’s Rule of Five and Veber’s criteria, with molecular weights < 500 Da, optimal TPSA, and MLogP values, supporting oral drug-likeness (Table 3, Figure 11A). Both analogues showed high predicted human intestinal absorption and were non-substrates of P-glycoprotein, reducing the likelihood of efflux-related bioavailability loss. The absence of predicted blood–brain barrier permeability suggests suitability for non-CNS indications (Figure 11B).

Distribution projections revealed organelle-specific localization, with compound **1** preferring lysosomes and compound **2** targeting mitochondria, which might be used for targeted therapeutic action. Metabolic study revealed that both are non-inhibitors of the majority of CYPs, implying a low frequency of drug–drug interactions and little effect on other active substances. Toxicity predictions revealed weak hERG I inhibition in both compounds, necessitating cardiotoxicity testing, although Ames and carcinogenicity tests were negative. Both have acute oral toxicity class III (771.1 and 728.4 mg·kg^−1^, respectively) and are not easily biodegradable, creating environmental persistence concerns (Table S8). Overall, the ADMET profile encourages further research into both drugs as orally active options with tolerable hazards.

#### 2.3.2. Docking Simulation

To justify the in vitro cytotoxic and enzyme inhibitory actions of Compounds **1** and **2**, the most potent synthetic 1,3-dimethyl-2,4-dioxopyrido[2,3-*d*]pyrimidine derivatives, against important cancer-related targets, EGFR^WT^ and mutant EGFR^T790M^, molecular docking studies were conducted. Docking simulations were carried out with MOE-Dock (version 2024.0601) [89,90,91,92]. The crystal structures of EGFR^WT^ and EGFR^T790M^, complexed with their reference ligands erlotinib and WZ4002 (PDB IDs: 1M17 and 3IKA) [78,93], were retrieved from the Protein Data Bank. Validation of the docking protocol using these native ligands resulted in low RMSD values (0.91 and 1.27 Å) and favorable binding energies (−11.14 and −10.31 kcal/mol), confirming the reliability of the procedure.

**Figure 12:**
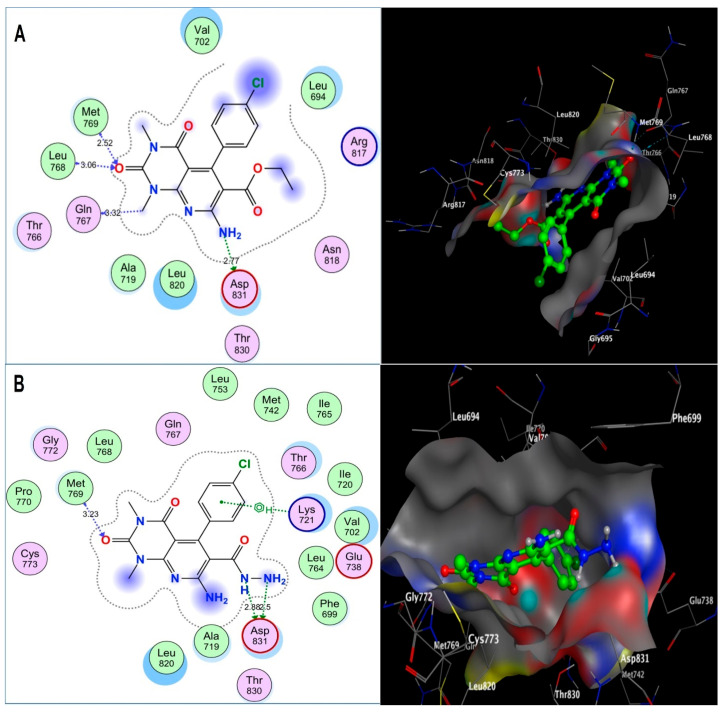
Panels (**A**) and (**B**) present the 2D and 3D binding orientations of the active 1,3-dimethyl-2,4-dioxopyrido[2,3-*d*]pyrimidine derivatives **1** and **2** inside the binding pocket of wild-type EGFR (PDB ID: 1M17), respectively.

Figure 12 shows that the derivatives **1** and **2** were positioned correctly within EGFR showing favorable binding energies of −10.93 and −10.68 kcal/mol, respectively. The oxygen of dioxopyrido[2,3-*d*]pyrimidine scaffold at p-2, afforded H-bond acceptor with the Met769 backbone in both derivatives, resembling the native ligand, and additional one with Leu768 in 1. Also, the methyl group at p-1 in compound 1 displayed an H-bond donor with Gln767 backbone (distance: 3.32 Å). The sidechain of Asp831 accepted one hydrogen bond from the amino group at p-7 in 1 and two from the hydrazide fragment at p-6 in 2 (distances: 2.77, 2.50, and 2.88 Å, respectively). Also, the centroid of 4-chlorophenyl in 1 applied arene-H interaction with Lys721.

**Figure 13:**
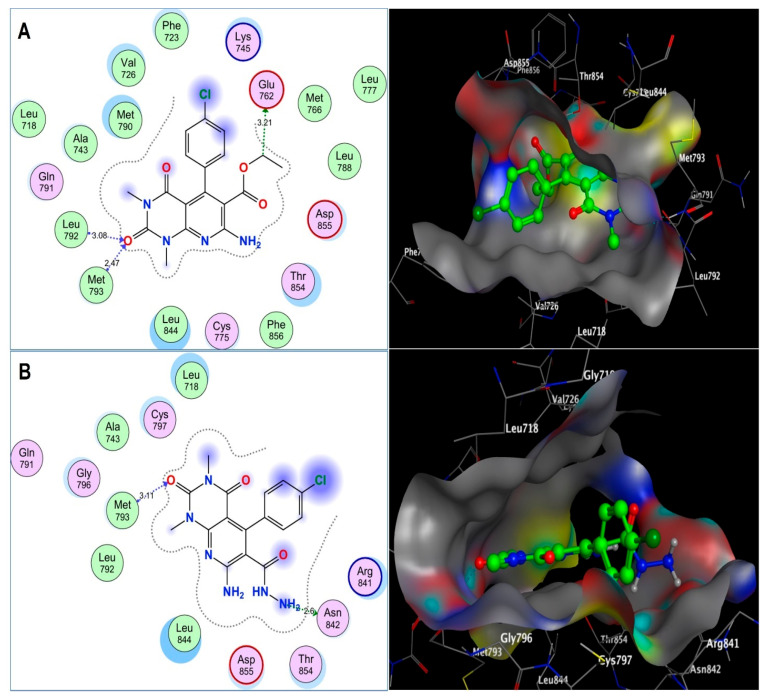
Diagrams (**A**,**B**) illustrate the 2D and 3D conformations of the active 1,3-dimethyl-2,4-dioxopyrido[2,3-*d*]pyrimidine derivatives **1** and **2** within the EGFR^T790M^ active site (PDB code: 3IKA), respectively.

By inspection of the EGFR^T790M^ receptor in Figure 13, the analogues **1** and **2** gave comparable energy scores of –9.91 and –9.22 kcal/mol, respectively. Also, H-bond acceptors were detected between the oxygen of dioxopyrido[2,3-*d*]pyrimidine scaffold at p-2 and the Met793 backbone in both derivatives, and another one with Leu792 in **1** (distances: 2.47, 3.11, and 3.08 Å, respectively). At p-6 of dioxopyrido[2,3-d]pyrimidine scaffold, an H-bond donor was demonstrated between the carboxylate in **1** and another one between hydrazide in **2** and Asn842 (distances: 3.21 and 2.60 Å, respectively).

**Figure 14:**
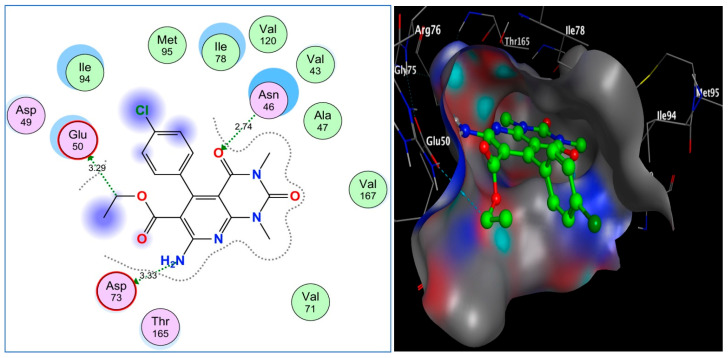
Two and three-dimensional views of promising 1,3-dimethyl-2,4-dioxopyrid [2,3-*d*]pyrimidines **1** within the *E. coli* DNA gyrase active site (PDB code: 1AJ6).

To confirm the mechanism of the predicted antimicrobial and antiviral potency of our target 1,3-dimethyl-2,4-dioxopyrido[2,3-*d*]pyrimidines **1**, a docking study was applied using the receptors of *E. coli* DNA gyrase and HCV NS5A (PDB codes: 1AJ6 & 3FQM, respectively) [94,95]. After validation of both receptors, giving tiny RMSD values of 0.78 and 1.38 Å and energy scores of −10.58 and −9.82 kcal/mol, respectively. Docking of compound **1** with both active sites resulted in promising energy scores of −10.25 and −9.36 kcal/mol, respectively, indicating perfect binding. Three H-bonds were created between the carbonyl oxygen at p-4, carboxylate at p-6, and the amino group at p-7 with the key amino acids Asp46, Asp73, and Glu50 sidechains (distances: 2.74, 3.33, and 3.29 Å, respectively) (Figure 14).

**Figure 15:**
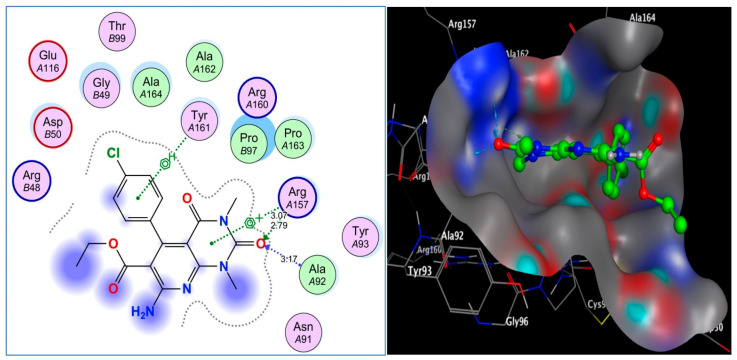
Two and three-dimensional views of promising 1,3-dimethyl-2,4-dioxopyrido[2,3-*d*]pyrimidines **1** within the HCV NS5A active site (PDB code: 3FQM).

Regarding HCV NS5A ATP-pocket, the carbonyl oxygen of dioxopyrido[2,3-*d*]pyrimidine scaffold at p-2 in 1 exhibited three H-bond acceptors, one with Ala92 backbone and two with Arg157 sidechain (distances: 3.17, 2.79, and 3.07 Å, respectively) (Figure 15). Furthermore, the centroids of pyrimidine and 4-chlorophenyl revealed arene-H interactions with Arg157 and Tyr161, respectively.

#### 2.3.3. Molecular Dynamics Simulations

**Figure 16:**
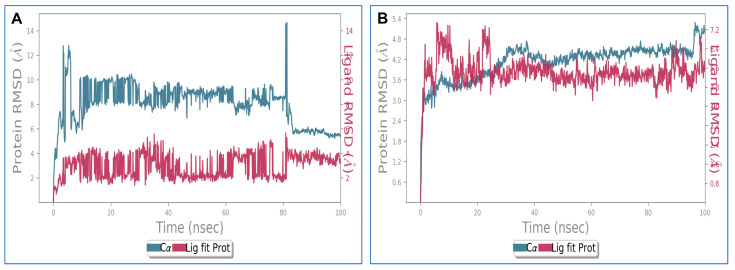
RMSD assessments for the MD simulations are shown in (**A**) and for 1,3-dimethyl-2,4-dioxopyrido[2,3-*d*]pyrimidine **1** with EGFR^WT^, and mutant EGFR^T790M^ in (**B**) respectively.

**Figure 17:**
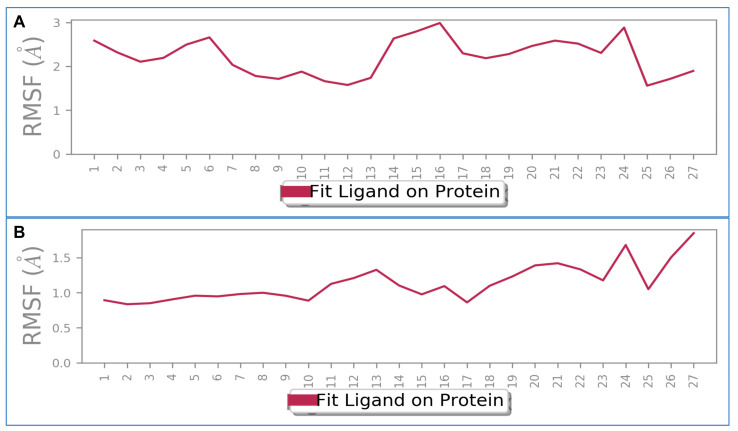
Molecular dynamics trajectory graphs (**A**,**B**) for 1,3-dimethyl-2,4-dioxopyrido[2,3-*d*]pyrimidine **1** with EGFR^WT^, and mutant EGFR^T790M^, respectively.

**Figure 18:**
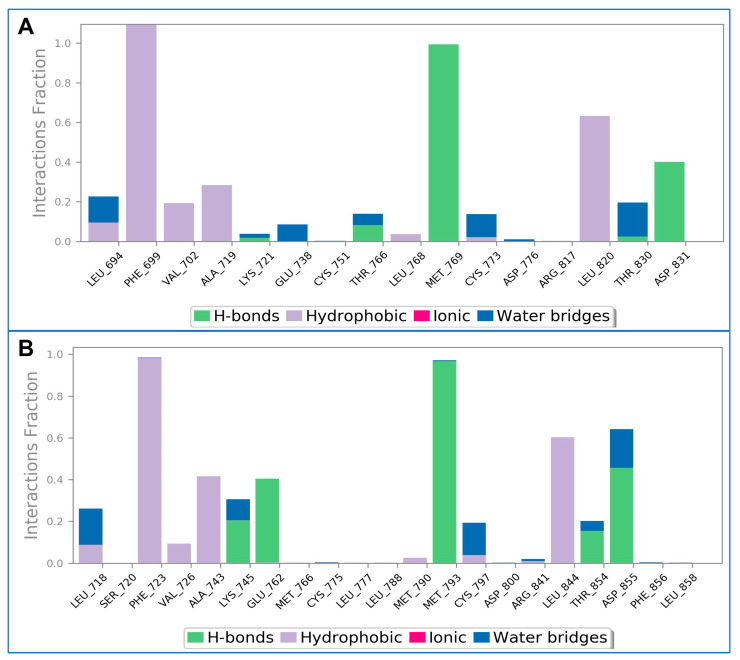
Trajectories (**A**,**B**) illustrate the interaction patterns of compound **1** (1,3-dimethyl-2,4-dioxopyrido[2,3-*d*]pyrimidine) with EGFR^WT^ and the ^T790M^ mutant, respectively.

**Figure 19:**
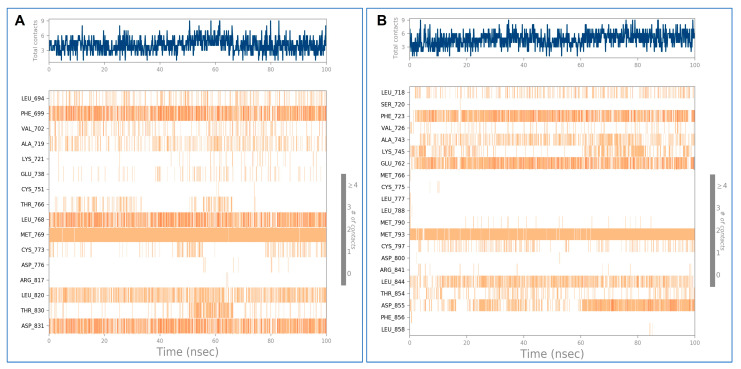
Timelines (**A**,**B**) illustrate the interaction and contact profiles of compound **1** (1,3-dimethyl-2,4-dioxopyrido[2,3-*d*]pyrimidine) with EGFR^WT^ and the ^T790M^ mutant, respectively.

Molecular dynamics simulations were subsequently applied to confirm the docking outcomes of compound **1** (1,3-dimethyl-2,4-dioxopyrido[2,3-*d*]pyrimidine) within the binding domains of both EGFR^WT^ and the T790M mutant [77,96]. The results, illustrated in Figure 16, Figure 17, Figure 18 and Figure 19, summarize the dynamic trajectories, including protein–ligand interactions, RMSD, and RMSF profiles.

Both the free EGFR^WT^ and mutant EGFR^T790M^ enzymes were found to be highly dynamic by RMSD estimates. The unbound protein of EGFR^WT^ achieved an average RMSD value of 9 Å in Figure 16A, which decreased upon binding with **1** (3 Å). Also, the plot of RMSD of **1** within EGFR^T790M^ indicated that the complex reached stability at 30 ns within the range of 3.5–4.6 Å, throughout the simulation (Figure 16B), suggesting that compound **1** was highly successful in reducing the dynamic behavior of EGFR^WT^, and mutant EGFR^T790M^.

With ranges of 1.8–3 Å and 0.9–2.0 Å for **1** within EGFR^WT^, and mutant EGFR^T790M^, respectively, the RMSF plots of protein-compound **1** complexes (Figure 17A,B) reflected a notable reduction. This suggests that derivative **1** remains tightly accommodated in the binding cavities of both EGFR^WT^ and the ^T790M^ mutant, thereby contributing to the stability of the formed complexes.

Derivative **1** continuously maintained a hydrogen connection with Met769 and Asp831 for 100% and 40% of the simulation time within EGFR^WT^, respectively (Figure 18A), demonstrating strong binding. However, within EGFR^T790M^, H-bonding with the residues Glu762, Met793, and Asp855 was highly observed for 40%, 98% and 43% of the simulation time, respectively (Figure 18B). Additionally, an intense orange color interacting with residues Phe699, Leu768, Met769, Leu820, and Asp831 within EGFR^WT^ (Figure 19A) and with Phe723, Glu762, Met793, Leu844, and Asp855 within EGFR^T790M^ revealed a remarkable situation of interactions for the screened derivative 1 with EGFR^WT^, and mutant EGFR^T790M^ residues over 100ns (Figure 19A,B).

#### 2.3.4. Quantum Chemical Calculation

##### Computational Profiling and Global Reactivity Descriptors

A series of sixteen compounds was evaluated using quantum chemical descriptors, including HOMO, LUMO, and the energy gap (ΔE), as well as conceptual DFT parameters: electronegativity (χ), ionization potential (I), chemical hardness (η), softness (S), electrophilicity index (ω), and charge transfer indices (ΔN and ΔN_max). Significant variations in the HOMO–LUMO gap were observed across the series. Compound **2** exhibited the narrowest gap (ΔE = 0.00478 eV), corresponding to extremely high softness (S = 418.41) and electrophilicity (ω = 12.89). In contrast, compound 1 had the widest gap (ΔE = 0.1064 eV) with lower softness (S = 18.80) and reduced electrophilicity (ω ≈ 0.92). The detailed quantum chemical values are summarized in Table 4, and the DFT-optimized structures along with HOMO/LUMO diagrams for selected compounds are provided in the Supplementary Information (Table S9). These results highlight the diverse electronic behavior of the compounds, suggesting potential structure–activity relationships.

##### Frontier Molecular Orbitals and Hardness/Softness

**Figure 20:**
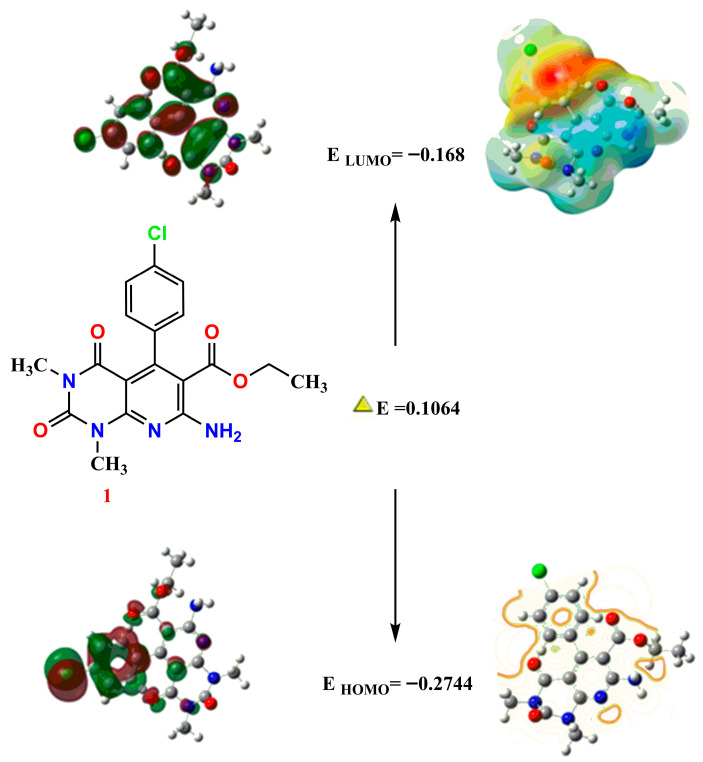

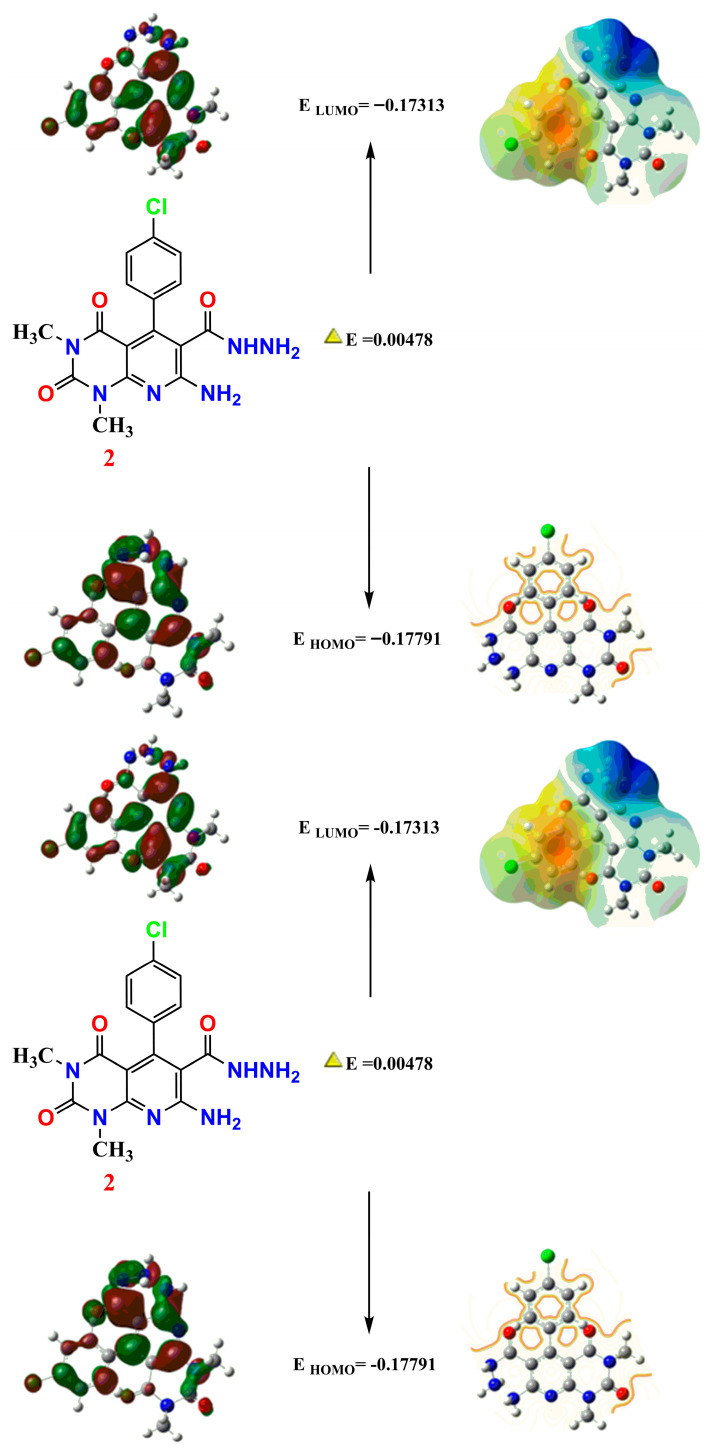
DFT-calculated geometries and frontier orbital (HOMO/LUMO) maps of compounds **1** and **2**.

Compound **1** has a very negative HOMO value of −0.2744, indicating a reduced tendency to donate electrons. Compounds **1**, **7**, and **9** have greater negative LUMO values of −0.16, indicating a higher ability to take electrons. According to the HSAB principle, molecules with smaller ΔE values are softer, more chemically reactive, and more prone to engage in polar interactions with biological targets. However, excessive softness, as observed in compound **2**, can result in uncontrolled reactivity and poor selectivity, emphasizing the importance of balanced electrical characteristics. geometries along with the HOMO and LUMO orbitals for the two top-ranked compounds (**1** and **2**) in Figure 20.

##### Electrostatic Potential (ESP) Mapping and Areal Distribution

Molecular electrostatic potential (ESP) surface analysis provided insights into the distribution of electron density. Positive (blue/cyan), negative (red/orange/yellow), and neutral (green) surface areas were quantified. ESP areas were extracted from color-thresholded MEP images using Python (OpenCV 3.13.7), and the resulting percentages were applied in the calculation of area balance and incorporated into the ESP Score.

Representative examples include compound **5** (Positive 26.86%, Negative 8.80%, area balance ≈ +18.06) and compound **12** (Positive 24.89%, Negative 6.00%, area balance ≈ +18.89), both showing strongly positive ESP distributions. Conversely, compound **2** displayed a predominantly negative ESP distribution (Positive 9.85%, Negative 14.82%, balance ≈ −4.97). These findings indicate that both global descriptors and localized ESP features are crucial in predicting bioactivity, as extended positive regions may enhance interactions with nucleophilic residues at biological target sites (see Supplementary Information, Tables S10 and S11).

##### Composite Bioactivity Score and Compound Ranking

A composite bioactivity score was calculated as: Composite Bioactivity Score = 0.30 × n(Softness) + 0.20 × n(ω) + 0.20 × n(inv ΔE) + 0.15 × ESP Score, where the ESP Score was derived from normalized ESP maxima, minima, contrast, and area balance.

Based on this model, the top-ranked compounds were:

- Compound **5** (1.0834): Balanced softness (S ≈ 34.30), moderate electrophilicity (ω ≈ 1.35), favorable ESP profile.
- Compound **12** (1.0668): Despite a higher ΔE (0.0776 eV), advantageous ESP distribution enhanced its ranking.
- Compound **2** (0.8306): Extremely low ΔE and high softness/electrophilicity, but negative ESP bias reduced its score.
- Compounds **4** (0.6380) and **1** (0.6363): Moderate predicted activity.

Intermediate activity was predicted for compounds **16** (0.3367), **13** (0.3264), **11** (0.2907), **15** (0.2756), **9** (0.2611), and **8** (0.2447), while compounds **7** (0.1758) and **10** (0.1695) were least favorable (Table 5).

##### Interplay Between Global and Local Descriptors

While softness and electrophilicity strongly influence predicted activity, ESP surface profiles were decisive in differentiating top-ranked compounds. Compounds **5** and **12** benefited from extended positive ESP regions and favorable area balances, compensating for moderate ΔE values. Conversely, compound **2**, despite remarkable global reactivity, was penalized by its negatively skewed ESP distribution.

##### Charge Transfer Capacity

High ΔN max values, as observed for compound **2**, indicate enhanced electron-accepting potential, consistent with its high ω and S values. However, this alone was insufficient for top ranking without complementary ESP features, highlighting the importance of integrating both global and local descriptors for reliable activity prediction.

##### Implications for SAR and Lead Prioritization

Compounds **5**, **12**, and **2** were identified as the top candidates for subsequent biological studies. Structural modifications that enhance positive ESP regions while maintaining moderate ΔE values are likely to improve activity. Additionally, avoiding excessive softness while preserving favorable ESP contrast and balance is recommended to ensure stability and selectivity.

##### Limitations and Outlook

The scoring system relies on internal min–max normalization, and results may vary with other chemical datasets. ESP features were derived from static conformations, which may change in different phases or conformers. Nevertheless, the agreement between compound rankings, conceptual DFT principles, and ESP distributions supports the model’s robustness. Experimental validation via docking, molecular dynamics, and ADMET studies is essential to confirm predictions.

##### Target Compound Selection (Compound **1**)

Although compounds **5** and **12** scored highest, compound **1** was selected as the target molecule due to its balanced electronic properties and superior stability. With the widest HOMO–LUMO gap (ΔE = 0.1064 eV), moderate softness (S ≈ 18.80), and relatively low electrophilicity (ω ≈ 0.92), compound **1** is expected to interact selectively with biological targets, combining synthetic feasibility, electronic stability, and acceptable reactivity. Therefore, it was advanced as the lead for subsequent experimental validation and biological assays.

## 3. Materials and Methods

### 3.1. Chemistry

The instruments employed included those for determining melting points and recording IR, ^1^H NMR, ^13^C NMR, and mass spectra are described in detail in the supplemental material file.

Using the previously published method, 6-amino-1,3-dimethylpyrimidine-2,4(1*H*,3*H*)-dione, the starting chemical, was produced [73].

#### 3.1.1. Synthesis of Ethyl 7-amino-5-(4-chlorophenyl)-1,3-dimethyl-2,4-dioxo-1,2,3,4-tetrahydropyrido[2,3-d]pyrimidine-6-carboxylate (**1**)

A mixture of 6-amino-1,3-dimethylpyrimidine-2,4(1*H*,3*H*)-dione (10 mmol, 1.55 g) and ethyl (*E*)-3-(4-chlorophenyl)-2-cyanoacrylate (10 mmol, 2.35 g) in 10 mL of anhydrous DMF with a catalytic quantity of triethylamine present. For 6h, the reaction was refluxed at 80–100 °C. Using eluent ethyl acetate: hexane (1:1), TLC was performed to track the progress of the reaction. Upon completion, the reaction mixture was cooled to room temperature and then poured into 50 mL of chilled water, resulting in precipitation. Compound **1** was obtained as a brown powder by vacuum filtering the resultant solid, extensively washed by cold ethanol, with the final step involving recrystallization from ethanol.

Yield 89%; m.p. 229–231 °C; IR (*ν_max_*/cm^−1^): 3410, 3332 (NH_2_), 3046 (CH-arom.), 2954, 2923, 2853 (CH-aliph.), 1735 (C=O ester), 1692, 1650 (2C=O amide), 1594 (C=N), 1565, 1528 (C=C arom.); ^1^H-NMR (DMSO-*d*_6_, 400 MHz): δ 1.71 (t, *J* = 7.2 Hz, 3H, CH_3_ ester), 3.07, 3.12 (s, 6H, 2CH_3_), 4.05 (q, *J* = 7.1 Hz, 2H, CH_2_), 7.30–7.89 (m, 4H, Ar-H), 8.48 (s, 2H, NH_2_, D_2_O exchangeable) ppm.; ^13^C-NMR (DMSO-*d*_6_, 100 MHz); *δ* 14.92 (1C, CH_3_ ester), 23.06, 26.03 (2C,2CH_3_), 57.80 (1C, CH_2_), 96.09 (1C, C pyridine-C=O ester), 102.97 (1C, C pyridine-C=O amide), 128.74, 129.37, 129.92, 130.48, 130.84, 131.39 (6C, C-Ar), 152.57 (1C, C pyridine-Ph-Cl), 155.00, 158.47 (2C, C=O uracil), 155.90 (1C, C=N pyridine), 161.97 (1C, C pyridine-N-CH_3_), 166.23(1C, C=O ester) ppm. Anal. Calcd for C_18_H_17_ClN_4_O_4_ (388.81): C, 55.61; H, 4.41; Cl, 9.12; N, 14.41; O, 16.46%. Found: C, 55.65; H, 4.40; Cl, 9.12; N, 14.43; O, 16.65 %.

#### 3.1.2. 7-Amino-5-(4-chlorophenyl)-1,3-dimethyl-2,4-dioxo-1,2,3,4-tetrahydropyrido[2,3-*d*]pyrimidine 6-carbohydrazide (**2**)

The solution was made by dissolving **1** (3.88 g, 10 mmol) in 10 mL of DMF while maintaining stirring at ambient temperature for 30 min. Hydrazine hydrate (1.25 mL, 25 mmol) was added gradually while stirring constantly following pre-equilibration. After that, the mixture was refluxed for 4 h at 70 °C. Using ethyl acetate: hexane (1:1) as the eluent, TLC verified the end of the reaction process. The resultant precipitate was recovered by vacuum filtering and properly cleaned with cold ethanol after cooling to RT. Compound **2**, a pale yellow powder, was obtained by further purifying the crude product by recrystallizing it from methanol.

Yield 81%; m.p. 200–202 °C; IR (*ν_max_*/cm^−1^): 3475, 3373 (NH_2_-pyridne), 3400, 3256 (NH_2_-NH), 3182 (NH), 3046 (ν CH-arom.), 2989, 2945 (ν CH-Aliph.), 1690, 1651 (2C=O amide), 1625 (C=O amide-NH), 1590 (C=N), 1561, 1543 (C=C arom.); ^1^H-NMR (DMSO-*d*_6_, 400 MHz,); δ 3.07, 3.12 (s, 6H, 2CH_3_), 6.24 (s, 2H, NH_2_-NH, D_2_O exchangeable), 7.31–8.27 (m, 4H, Ar-H), 8.47 (s, 2H, NH_2_-pyridine, D_2_O exchangeable), 9.10 (s, 1H, NH, D_2_O exchangeable) ppm; ^13^C-NMR (DMSO-*d*_6_, 100 MHz); δ 23.05, 26.06 (2C, 2CH_3_), 102.14 (2C, 2C pyridine-C=O), 127.60, 128.42, 128.71, 129.18, 129.64, 131.37 (6C, C-Ar), 152.89 (1C, C pyridine-Ph-Cl), 154.46, 158.46 (2C, C=O uracil), 156.89 (1C, C=N pyridine), 162.14 (1C, C pyridine-N-CH_3_), 166.61 (1C, C=O-NH amide) ppm. Anal. Calcd for C_16_H_15_ClN_6_O_3_ (374.79): C, 51.28; H, 4.03; Cl, 9.46; N, 22.42; O, 12.81%. Found: C, 51.29; H, 4.01; Cl, 9.48; N, 22.42; O, 12.81%.

#### 3.1.3. 7-Amino-5-(4-chlorophenyl)-*N*-(4-hydrazinyl-1*H*-pyrazol-3-yl)-1,3-dimethyl-2,4-dioxo-1,2,3,4-tetrahydropyrido[2,3-*d*]pyrimidine-6-carboxamide (**3**)

A weighed portion of compound **1** (3.88 g, 10 mmol) was first dissolved in 10 mL of *GL* AcOH and stirred at ambient temperature for 30 min. Once equilibrium was established, 3-amino-5-hydrazinyl pyrazole (1.13 g, 10 mmol) was introduced gradually with continuous stirring. The reaction mixture was then subjected to reflux at 80 °C for eight hours. Progress of the reaction was monitored by TLC using ethyl acetate/hexane (1:1) as the mobile phase. After completion, the mixture was cooled to room temperature, and the formed solid was separated by vacuum filtration and washed thoroughly with cold ethanol. The crude material was recrystallized from ethyl acetate to yield compound **3** as a brown crystalline powder.

Yield 72%; m.p. 208–210 °C; IR (*ν_max_*/cm^−1^): 3348, 3202 (2NH_2_, 3NH), 3050 (CH-arom.), 2945 (CH-Alipha.), 1710, 1658 (2C=O amide), 1625 (C=O amide-NH), 1560 (C=N pyridine), 1517 (C=N pyrazole), 1492 (C=C arom.); ^1^H-NMR (DMSO-*d*_6_, 400 MHz,); *δ* 3.04, 3.19 (s, 6H, 2CH_3_), 4.43 (s, 2H, NH_2_-NH, D_2_O exchangeable), 7.32–7.46 (m, 4H, Ar-H), 7.82 (s, 1H, CH pyrazole), 8.40 (s, 2H, NH_2_-pyridine, D_2_O exchangeable), 9.69 (s, 1H, NH-NH_2_, D_2_O exchangeable), 11.30 (s, 1H, NH-C=O, D_2_O exchangeable), 12.27 (s, 1H, NH pyrazole, D_2_O exchangeable) ppm; ^13^C-NMR (DMSO-*d*_6_, 100 MHz) *δ* = 23.03, 26.52 (2C, 2CH_3_), 102.00 (2C, 2C pyridine-C=O), 118.58 (1C, C pyrazole-NHNH_2_), 127.67 (1C, CH pyrazole), 128.42, 128.77, 128.94, , 129.51, 130.42, 131.18 (6C, C-Ar), 142.98 (1C, C=N pyrazole), 151.13 (1C, C pyridine-Ph-Cl), 154.20, 158.39 (2C, 2C=O uracil), 156.55 (1C, C pyridine-N-CH_3_), 158.99 (1C, C=N pyridine), 169.77 (1C, C=O amide-pyridine) ppm. Anal. Calcd for C_19_H_18_ClN_9_O_3_ (455.86): C, 50.06; H, 3.98; Cl, 7.78; N, 27.65; O, 10.53%. Found C, 50.08; H, 4.00; Cl, 7.79; N, 27.63; O, 10.53%.

#### 3.1.4. 7-Amino-6-(4-amino-5-mercapto-4*H*-1,2,4-triazol-3-yl)-5-(4-chlorophenyl)-1,3-dimethylpyrido[2,3-*d*]pyrimidine-2,4(1*H*,3*H*)-dione (**4**)

To form the mercapto triazole ring, dissolving of Compound **1** (3.88 g, 10 mmol) in 10 mL of NaOEt. Then, with continual stirring, hydrazine carbo thiohydrazide (1.06 g, 10 mmol) was added gradually. The mixture was then refluxed for 12 h at 80 °C. TLC confirmed that everything was going according to plan with the reaction. After cooling the precipitate to room temperature, it was collected, and cold methanol was used to thoroughly clean the solid. The crude product from dioxane was recrystallized to increase purity, and compound **4** was produced as a brownish-yellow powder.

Yield 76%; m.p. 296–298 °C; IR (*ν_max_*/cm^−1^): 3354, 3204 (2NH_2_), 3064 (CH-arom.), 2928 (CH-Aliph.), 2346 (SH), 1716, 1656 (2 C=O), 1597 (C=N pyridine), 1551, 1507 (2C=N pyrazole), 1491 (C=C arom.); ^1^H-NMR (DMSO-*d*_6_, 400 MHz,); *δ* 3.06, 3.11 (s, 6H, 2CH_3_), 5.09 (s, 2H, NH_2_-pyrazole, D_2_O exchangeable), 7.25–7.57 (m, 4H, Ar-H), 7.93 (s, 2H, NH_2_-pyridine, D_2_O exchangeable), 13.45 (s, 1H, SH) ppm; ^13^C-NMR (DMSO-*d*_6_, 100 MHz); *δ* 23.09, 26.34 (2C, 2CH_3_), 102.24 (1C, C pyridine-C=O), 107.35 (1C, C pyridine-triazole), 127.54, 128.29, 128.95, 129.34, 130.42, 131.16 (6C, C-Ar), 152.04 (1C, C=N triazole-pyridine), 152.67 (1C, C pyridine-Ph-Cl), 154.81 (1C, C pyridine-N-CH_3_), 154.94, 158.84 (2C, 2C=O uracil), 155.82 (1C, C=N pyridine), 169.00 (1C, C=N triazole-SH) ppm; MS: *m*/*z* = 430.63 [M^+^], 186 (100%). Anal. Calcd for C_17_H_15_ClN_8_O_2_S (430.87): C, 47.39; H, 3.51; Cl, 8.23; N, 26.01; O, 7.43; S, 7.44%. Found C, 47.42; H, 3.59; Cl, 8.25; N, 26.00; O, 7.43; S, 7.44%.

#### 3.1.5. 7-Amino-5-(4-chlorophenyl)-*N*-(hydrazinecarbonothioyl)-1,3-dimethyl-2,4-dioxo-1,2,3,4-tetrahydropyrido[2,3-*d*]pyrimidine-6-carboxamide (**5**)

After dissolving Compound **1** (3.88 g, 10 mmol) in 10 mL of *Gl*. AcOH, the mixture was agitated at R.T. for 30 min. Thiosemicarbazide (0.92 g, 10 mmol) was added gradually while being continuously agitated once equilibrium was reached. The mixture was refluxed at 80 °C for 18 h, and progress was monitored by TLC (EtOAc/hexane, 1:1). After completion, the precipitate was filtered, washed with cold ethanol, and recrystallized from ethanol to give compound **5** as a dark yellow powder.

Yield 80%; m.p. ˃ 300 °C; IR (*ν_max_*/cm^−1^): 3441, 3281 (NH_2_-pyridine), 3349, 3254 (NH_2_-NH), 3201, 3127 (2NH), 3046 (CH- arom.), 2931 (CH-Aliph.), 1716, 1656 (2C=O uracil), 1597 (C=O amide-NH), 1560 (C=N), 1516, 1491 (C=C arom.), 1090 (C=S); ^1^H-NMR (DMSO-*d*_6_, 400 MHz,); *δ* 3.01 (s, 2H, NH_2_-NH, D_2_O exchangeable), 3.08, 3.10 (s, 6H, 2CH_3_), 7.30–7.91 (m, 4H, Ar-H), 7.93 (s, 2H, NH_2_-pyridine, D_2_O exchangeable), 10.89 (s, 1H, NH-NH_2_, D_2_O exchangeable), 12.06 (s, 1H, NH-CO, D_2_O exchangeable) ppm; ^13^C-NMR (DMSO-*d*_6_, 100 MHz); *δ* 23.07, 26.37 (2C,2CH_3_), 105.24 (2C, 2C pyridine-C=O amide), 127.56, 129.36, 137.85 (6C, C-Ar), 151.40 (1C, C pyridine-Ph-Cl), 154.63, 158.94 (2C, C=O uracil), 157.28 (1C, C=N pyridine), 161.97 (1C, C pyridine-N-CH_3_), 171.62 (1C, C=O amide), 186.05 (1C, C=S) ppm. Anal. Calcd for C_17_H_16_ClN_7_O_3_S (433.87): C, 47.06; H, 3.72; Cl, 8.17; N, 22.60; O, 11.06; S, 7.39%. Found C, 47.16; H, 3.02; Cl, 8.17; N, 22.61; O, 11.04; S, 7.39%.

#### 3.1.6. 7-Amino-5-(4-chlorophenyl)-6-(3,5-dimethyl-1*H*-pyrazole-1-carbonyl)-1,3-dimethylpyrido[2,3-*d*]pyrimidine-2,4(1*H*,3*H*)-dione (**6**)

Compound **2** (3.75 g, 10 mmol) and acetylacetone (1.02 mL, 10 mmol) were dissolved in 10 mL of sodium ethoxide solution and refluxed at 80 °C for 8 h. Reaction progress was checked by TLC. After cooling, the mixture was poured into 50 mL of ice-cold water, and the precipitate was collected by filtration, washed with cold methanol, and recrystallized from ethanol to yield compound **6** as orange crystals.

Yield 70%; m.p. 239–241 °C; IR (*ν_max_*/cm^−1^): 3384, 3304 (NH_2_), 3073 (CH- arom.), 2983, 2934, 2819, 2736 (CH-Alipha.), 1740 (C=O-pyrazole), 1688, 1650 (2C=O amide), 1591 (C=N pyridine), 1530 (C=N pyrazole), 1500 cm^−1^ (ν C=C arom.); ^1^H-NMR (DMSO-*d*_6_, 400 MHz,); *δ* 2.71, 2.87 (s, 6H, 2CH_3_ pyrazole), 3.08, 3.12 (s, 6H, 2CH_3_ uracil), 6.24 (s, 1H, CH pyrazole), 7.31–8.41 (m, 4H, Ar-H), 8.49 (s, 2H, NH_2_, D_2_O exchangeable) ppm; ^13^C-NMR (DMSO-*d*_6_, 100 MHz); *δ* 13.04, 13.97 (2C, 2CH_3_ pyrazole), 23.19, 26.67 (2C, 2CH_3_), 102.00, 104.11 (2C, 2C pyridine-C=O), 110.02 (1C, CH pyrazole), 127.27, 127.61, 128.02, 128.51, 129.49, 131.06 (6C, C-Ar), 141.64 (1C, C pyrazole-CH_3_), 151.06 (1C, C=N pyrazole), 154.93 (1C, C pyridine-Ph-Cl), 154.93, 158.83 (2C, C=O uracil), 161.79 (1C, C=N pyridine), 162.48 (1C, C pyridine-N-CH_3_), 165.81 (1C, C=O amide) ppm; MS: *m*/*z* = 438.87 [M^+^], 132 (100%). Anal. Calcd for C_21_H_19_ClN_6_O_3_ (430.87): C, 57.47; H, 4.36; Cl, 8.08; N, 19.15; O, 10.94%. Found C, 57.49; H, 4.41; Cl, 8.08; N, 19.13; O, 10.97%.

#### 3.1.7. 7-Amino-5-(4-chlorophenyl)-6-(5-mercapto-1,3,4-oxadiazol-2-yl)-1,3-dimethylpyrido[2,3-*d*]pyrimidine-2,4(1*H*,3*H*)-dione (**7**)

By dissolving compound **2** (3.745 g, 10 mmol) in 8 mL of DMF, the mercapto-oxadiazole moiety formed. After adding carbon disulfide (0.6 g, 10 mmol), the reaction was conducted and refluxed with potassium hydroxide (KOH) acting as a catalyst for 4 h at 100 °C in a water bath. (TLC) was used to validate the reaction’s development. To induce precipitation, 50 mL of crushed ice was added to the reaction mixture after cooling to R.T. The resulting solid was recovered by vacuum filtering and treated with cold ethanol to eliminate residual impurities. Pure chemical **7** was created as a pale yellow crystalline powder by recrystallizing ethanol.

Yield (85%); m.p. 256–258 °C; IR (*ν_max_*/cm^−1^): 3411, 3347 (NH_2_), 3042 (CH-arom.), 2967, 2923 (CH-Aliph.), 2596 (SH), 1686, 1654 (2C=O amide), 1599, 1588 (2C=N oxadiazole), 1553 (C=N pyridine), 1465 (C=C arom.); ^1^H-NMR (DMSO-*d*_6_, 400 MHz,); *δ* 3.07, 3.10 (s, 6H, 2CH_3_), 7.30–8.18 (m, 4H, Ar-H), 8.50 (s, 2H, NH_2_, D_2_O exchangeable), 13.80 (s, 1H, SH) ppm; ^13^C-NMR (DMSO-*d*_6_, 100 MHz); *δ* 23.00, 26.00 (2C, 2CH_3_), 102.94 (1C, C pyridine-C=O), 107.77 (1C, C pyridine-oxadiazole), 127.60, 128.71, 129.32, 129.64, 129.92, 131.37 (6C, C-Ar), 152.00 (1C, C pyridine-Ph-Cl), 154.43, 158.26 (2C, 2C=O uracil), 154.62 (1C, C=N pyridine), 162.22 (1C, C pyridine-N-CH_3_), 166.66 (1C, C=N oxadiazole-pyridine), 169.59 (1C, C=N triazole-SH) ppm. Anal. Calcd for C_17_H_13_ClN_6_O_3_S (416.84): C, 48.98; H, 3.14; Cl, 8.50; N, 20.16; O, 11.51; S, 7.69%. Found C, 49.01; H, 3.18; Cl, 8.52; N, 20.18; O, 11.51; S, 7.69%.

#### 3.1.8. 3-Amino-5-[7-amino-5-(4-chlorophenyl)-1,3-dimethyl-2,4-dioxo-1,2,3,4-tetrahydropyrido[2,3-*d*]pyrimidin-6-yl]-4*H*-pyrazole-4-carbonitrile (**8**)

Compound **2** (3.75 g, 10 mmol) and malononitrile (0.58 mL, 10 mmol) were diluted in 10 mL of DMF and added. TLC was used to track the reaction’s progress over six hours of reflux at 90 °C. After finishing, the mixture was let to cool to room temperature, which caused a precipitate to form. Vacuum filtration was used to gather the solid product, and cold methanol was used to wash it. By recrystallizing an ethanol/water combination, pure chemical **8** was ultimately produced as an off-white powder.

Yield 77%; m.p. ˃ 300 °C; IR (*ν_max_*/cm^−1^): 3416, 3216 (2NH_2_), 3061 (CH-arom.), 2924, 2861 (CH-Alipha.), 2222 (CN), 1694, 1644 (2 C=O amide), 1597 (C=N pyridine), 1555, 1517 (2C=N pyrazole), 1504 (C=C arom.); ^1^H-NMR (DMSO-*d*_6_, 400 MHz,); *δ* 2.79 (s, 1H, CH pyrazole), 3.08, 3.12 (s, 6H, 2CH_3_), 6.23 (s, 2H, NH_2_-pyrazole, D_2_O exchangeable), 7.29–8.40 (m, 4H, Ar-H), 8.49 (s, 2H, NH_2_-pyridine, D_2_O exchangeable) ppm;^13^C-NMR (DMSO-*d*_6_, 100 MHz); δ 23.06, 26.26 (2C, 2CH_3_), 33.50 (1C, CH pyrazole), 100.24 (1C, C pyridine-pyrazole), 103.39 (1C, C pyridine-C=O), 118.03 (1C, CN), 128.62,128.95, 129.45, 129.57, 130.48, 131.03 (6C, C-Ar), 152.69 (1C, C pyridine-Ph-Cl), 154.21, 158.00 (2C, C=O uracil), 157.17 (1C, C=N pyridine), 162.00 (1C, C pyridine-N-CH_3_), 165.80 (1C, C=N pyrazole-pyridine), 168.90 (1C, C=N pyrazole-NH_2_) ppm. Anal. Calcd for C_19_H_15_ClN_8_O_2_ (422.83): C, 53.97; H, 3.58; Cl, 8.38; N, 26.50; O, 7.57%. Found C, 53.99; H, 3.62; Cl, 8.41; N, 26.50; O, 7.59%.

#### 3.1.9. 3-{5-[7-Amino-5-(4-chlorophenyl)-1,3-dimethyl-2,4-dioxo-1,2,3,4-tetrahydropyrido [2,3-*d*]pyrimidin-6-yl]-2-thioxo-1,3,4-oxadiazol-3(2*H*)-yl}propanenitrile (**9**)

Using triethylamine as a catalytic base, compound **7** (4.16 g, 10 mmol) and acrylonitrile (0.65 mL, 10 mmol) were mixed in 10 mL of DMF. (TLC) was used to track the mixture’s progress over a 6-hours reflux at 100 °C. Once finished, the reaction mixture was cooled to ambient temperature and subsequently added to 50 mL of ice-cold water, which caused a precipitate to form. To separate out any remaining contaminants, the crude product was collected via suction filtration and purified by washing with cold ethanol. Following recrystallization from DMF, pure compound **9** was produced as a brown powder.

Yield 75%; m.p. 229–231 °C; IR (*ν_max_*/cm^−1^): 3416, 3325 (NH_2_), 3028 (CH-arom.), 2989, 2945, 2920, 2873 (CH-Aliph.), 2249 (CN), 1686, 1666 (2C=O amide), 1619 (C=N oxadizole), 1599 (C=N pyridine), 1522 (C=C arom.), 1058 (C=S); ^1^H-NMR (DMSO-*d*_6_, 400 MHz): δ 3.09, 3.17 (s, 6H, 2CH_3_), 3.91 (t, *J* = 7.2 Hz, 2H, CH_2_-CN), 4.15 (t, *J* = 7.2 Hz, 2H, CH_2_), 7.28–7.63 (m, 4H, Ar–H), 8.51 (s, 2H, NH_2_, D_2_O exchangeable) ppm. ^13^C-NMR (DMSO-*d*_6_, 100 MHz); *δ* 16.10 (1C, CH_2_-CN), 23.02, 26.04 (2C, 2CH_3_), 45.70 (1C, CH_2_-oxadiazole), 100.04 (1C, C pyridine- oxadiazole), 102.24 (1C, C pyridine-C=O), 118.03 (1C, CN), 128.62, 128.8, 128.95, 129.57, 130.48, 131.03 (6C, C-Ar), 153.03 (1C, C pyridine-Ph-Cl), 154.90, 158.47 (2C, 2C=O uracil), 158.96 (1C, C=N pyridine), 159.26 (1C, C=N oxadiazole), 160.08 (1C, C pyridine-N-CH_3_), 174.00 (1C, C=S) ppm. Anal. Calcd for C_20_H_16_ClN7O_3_S (469.90): C, 51.12; H, 3.43; Cl, 7.54; N, 20.87; O, 10.21; S, 6.82%. Found C, 51.152; H, 3.48; Cl, 7.58; N, 20.89; O, 10.21; S, 6.82%.

#### 3.1.10. 6-{4-[2-(2*H*-Tetrazol-5-yl)ethyl]-5-thioxo-4,5-dihydro-1,3,4-oxadiazol-2-yl}-7-amino-5-(4-chlorophenyl)-1,3-dimethylpyrido[2,3-*d*]pyrimidine-2,4(1*H*,3*H*)-dione (**10**)

To carry out the transformation (Tetrazole synthesis), compound **9** (4.16 g, 10 mmol) was stirred with sodium azide (0.65 g, 10 mmol) in 10 mL of DMF. A catalyst, ammonium chloride, was used to accelerate the reaction. TLC analysis was used to track the reaction’s progress during the mixture’s 10-hours reflux at 90 °C. Following completion, Once the reaction liquid had cooled to room temperature, 50 milliliters of crushed ice were added to cause precipitation. Compound **10** was obtained as a bright dark brown powder by vacuum filtering the solid, thoroughly cleaning it with cold ethanol to eliminate any leftover impurities, and then refining it via recrystallization from methanol.

Yield 66%; m.p. ˃ 300 °C; IR (*ν_max_*/cm^−1^): 3444, 3372 (NH_2_), 3282 (NH), 3072 (CH- arom.), 2961, 2832 (CH-Aliph.), 1683, 1636 (2C=O amide), 1603 (C=N oxadiazole), 1539 (C=N pyridine), 1491 (C=N tetrazole), 1466 (C=C arom.), 1297 (N=N), 1092 (C=S); ^1^H-NMR (DMSO-*d*_6_, 400 MHz), *δ* 3.07, 3.18 (s, 6H, 2CH_3_), 2.71, 4.16 (t, *J* = 7.0 Hz, 4H, 2CH_2_), 7.29–7.93 (m, 4H, Ar-H), 8.49 (s, 2H, NH_2_, D_2_O exchangeable), 14.00 (s, 1H, NH, D_2_O exchangeable_)_ ppm; ^13^C-NMR (DMSO-d_6_, 100 MHz) δ = 22.00 (1C, CH_2_-tetrazole), 23.06, 26.03 (2C, 2CH_3_), 54.89 (1C, CH_2_-N), 100.03 (1C, C pyridine-oxadiazole), 102.06 (1C, C pyridine-CO), 126.73, 127.84, 128.03, 129.78, 130.66, 131.00 (6C, C-Ar), 153.26 (1C, C pyridine-Ph-Cl), 154.46, 158.03 (2C, 2C=O uracil), 158.85 (1C, C=N pyridine), 159.44 (1C, C=N oxadiazole), 161.94 (1C, C uracil-NCH3), 165.94 (1C, C=N tetrazole), 174.12 (1C, C=S) ppm; MS: *m*/*z* = 512.93 [M^+^], 80 (100%). Anal. Calcd for C_20_H_17_ClN_10_O_3_S (512.93): C, 46.83; H, 3.34; Cl, 6.91; N, 27.31; O, 9.36; S, 6.25%. Found C, 46.83; H, 3.348; Cl, 6.961; N, 27.30; O, 9.36; S, 6.258%.

#### 3.1.11. 7-Amino-5-(4-chlorophenyl)-1,3-dimethyl-6-(4-oxo-3a,5-dihydro-4*H*-pyrazolo [3,4-*d*]pyrimidin-3-yl)pyrido[2,3-*d*]pyrimidine-2,4(1*H*,3*H*)-dione (**11**)

Compound **8** (4.22 g, 10 mmol) and formic acid (0.4 mL, 10 mmol) was heated in a sand bath at 60 °C for 10 h. Following completion, the mixture was allowed to cool to ambient temperature before being added to 50 mL of crushed ice which caused the crude product to precipitate. The precipitate was gathered by vacuum filtering and meticulously washed with cold ethanol to remove impurities. After being purified from chloroform by recrystallization, compound **11** was obtained as a black powder.

Yield 68%; m.p. ˃ 300 °C; IR (*ν_max_*/cm^−1^): 3424, 3363 (NH_2_), 3161 (NH), 3060 (CH-arom.), 2974, 2923, 2870 (CH-Aliph.), 1723 (C=O lactam), 1683, 1666 (2C=O amide), 1635 (C=N pyrimidinone), 1585 (C=N pyridine), 1550, 1514 (2C=N pyrazole), 1483 (C=C arom.); ^1^H-NMR (DMSO-*d*_6_, 400 MHz,); *δ* 2.32 (s, 1H, CH pyrazole), 3.06, 3.14 (s, 6H, 2CH_3_), 7.20–8.26 (m, 4H, Ar-H), 8.38 (s, 1H, CH pyrimidinone), 8.44 (s, 2H, NH_2_, D_2_O exchangeable), 11.44 (s, 1H, NH, D_2_O exchangeable) ppm; ^13^C-NMR (DMSO-*d*_6_, 100 MHz); δ 23.06, 26.13 (2C, 2CH_3_), 46.83 (1C, CH pyrazole), 100.42 (1C, C pyridine-pyrazole), 103.09 (1C, C pyridine-C=O), 128.42, 128.58, 128.71, 129.44, 130.98, 134.13 (6C, C-Ar), 150.98 (1C, C=N pyrimidinone), 151.63 (1C, C pyridine-Ph-Cl), 154.82, 158.17 (2C, C=O uracil), 157.10 (1C, C=N pyridine), 162.81 (1C, C pyridine-N-CH_3_), 165.76 (1C, C=N pyrazole-pyridine), 169.85 (1C, C=O pyrimidinone), 181.01 (1C, C=N pyrazole- pyrimidinone) ppm; MS: *m*/*z* = 450 [M^+^], 319 (100%). Anal. Calcd for C_20_H_15_ClN_8_O_3_ (450.84): C, 53.28; H, 3.35; Cl, 7.86; N, 24.85; O, 10.65%. Found C, 53.30; H, 3.37; Cl, 7.89; N, 24.85; O, 10.66%.

#### 3.1.12. 7-Amino-6-(4-amino-3a*H*-pyrazolo [3,4-*d*]pyrimidin-3-yl)-5-(4-chlorophenyl)-1,3-dimethylpyrido[2,3-*d*]pyrimidine-2,4(1*H*,3*H*)-dione (**12**)

Compound **8** (4.22 g, 10 mmol) and formamide (0.45 mL, 10 mmol) were fused for 12 h at 60 °C in a sand bath. After completing, the mixture was cooled to room temperature, which resulted in the appearance of a precipitate. The resulting solid was recovered by vacuum filtering and purifying by cold ethanol. A black powder representing pure component **12** was obtained when it was recrystallized from ethanol.

Yield 65%; m.p. 198–200 °C; IR (*ν _max_*/cm^−1^): 3422 (2NH_2_), 3067 (CH-arom.), 2964, 2919, 2821 (CH-Aliph.), 1702, 1646 (2C=O amide), 1610, 1594 (2C=N-dihydropyrimidin-4-amine), 1522 (C=N pyridine), 1517, 1492 (2C=N pyrazole), 1415 (C=C arom.); ^1^H-NMR (DMSO-*d*_6_, 400 MHz,); *δ* 1.65 (s, 1H, CH pyrazole), 3.04, 3.12 (s, 6H, 2CH_3_), 7.14–8.14 (m, 4H, Ar-H), 8.25, 8.50 (s, 4H, 2NH_2_, D_2_O exchangeable), 8.91 (s, 1H, CH pyrimidine) ppm; ^13^C-NMR (DMSO-*d*_6_, 100 MHz) *δ* = 23.06, 26.23 (2C, 2CH_3_), 35.86 (1C, CH pyrazole), 100.10 (1C, C pyridine-pyrazole), 102.50 (1C, 1C pyridine-C=O), 127.22, 127.69, 128.47, 128.81, 130.99, 131.46 (6C, C-Ar), 152.07 (1C, C pyridine-Ph-Cl), 154.22, 158.42 (2C, C=O uracil), 156.62 (1C, C=N pyridine), 162.98 (1C, C pyridine-N-CH_3_), 162.42 (1C, C=N pyrimidine-NH_2_), 163.09 (1C, C=N pyrimidine), 167.12 (1C, C=N pyrazole-pyridine), 178.02 (1C, C=N pyrazole) ppm. Anal. Calcd for C_20_H_16_ClN_9_O_2_ (449.86): C, 53.40; H, 3.59; Cl, 7.88; N, 28.02; O, 7.11%. Found C, 53.42; H, 4.04; Cl, 7.90; N, 28.05; O, 7.11%.

#### 3.1.13. 7-Amino-5-(4-chlorophenyl)-*N*-(3,5-dimethyl-1′*H*-[1,4′-bipyrazol]-3′-yl)-1,3-dimethyl-2,4-dioxo-1,2,3,4-tetrahydropyrido[2,3-*d*]pyrimidine-6-carboxamide (**13**)

To synthesize compound **13**, a 50 mL rounded flask was filled with 10 mL of DMF, and compound 3 (4.55 g, 10 mmol) and acetylacetone (1.02 mL, 10 mmol) were dissolved in it. After adding 5 drops of triethylamine as a catalytic base, the mixture was refluxed for twelve hours at 90 °C. After finishing, the mixture was let to cool to R.T, which caused a precipitate to form. The separated solid was obtained by suction filtration and carefully washed with cold methanol to ensure purity. Pure compound 13 was obtained as an orange powder through recrystallization from ethanol.

Yield 69%; m.p. 268–270 °C; IR (*ν_max_*/cm^−1^): 3377, 3322 (NH_2_), 3275, 3250 (2NH), 3063 (CH-arom.), 2980, 2922, 2853 (CH-Aliph.), 1700, 1650 (2C=O uracil), 1596 (C=O amide), 1572, 1520, 1491 (3C=N), 1427 (C=C arom.); ^1^H-NMR (DMSO-*d*_6_, 400 MHz,); *δ* 1.89, 2.10 (s, 6H, 2CH_3_ pyrazole), 3.02, 3.16 (s, 6H, 2CH_3_), 5.04 (s, 1H, CH methyl pyrazole), 7.24–7.46 (m, 4H, Ar-H), 7.83 (s, 1H, CH pyrazole), 8.41 (s, 2H, NH_2_, D_2_O exchangeable), 11.00 (s, 1H, NH-C=O, D_2_O exchangeable), 12.22 (s, 1H, NH pyrazole, D_2_O exchangeable) ppm; ^13^C-NMR (DMSO-*d*_6_, 100 MHz); *δ* 12.32, 13.50 (2C, 2CH_3_ pyrazole), 23.16, 26.29 (2C, 2CH_3_), 90.00 (1C, C pyrazole-di methyl pyrazole), 102.22 (2C, 2C pyridine-C=O), 112.25 (1C, CH di methyl pyrazole), 127.52, 127.72, 128.4, 129.58, 130.41, 131.76 (6C, C-Ar), 138.60 (1C, CH pyrazole), 142.20 (1C, C=N methyl pyrazole), 155.35 (1C, 1C di methyl pyrazole), 154.38, 158.26 (2C, 2C=O uracil), 156.03 (1C, C=N pyrazole), 156.76 (2C, 2C=C fused), 162.26 (1C, C=N pyridine), 169.96 (1C, C=O amide) ppm; MS: *m*/*z* = 519 [M^+^], 300 (100%). Anal. Calcd for C_24_H_22_ClN_9_O_3_ (519.95): C, 55.44; H, 4.27; Cl, 6.82; N, 24.25; O, 9.23%. Found C, 55.42; H, 4.27; Cl, 6.84; N, 24.25; O, 9.25%.

#### 3.1.14. 7-Amino-6-(6-chloro-7H-[1,2,4]triazolo [3,4-b][1,3,4]thiadiazin-3-yl)-5-(4-chlorophenyl)-1,3-dimethylpyrido[2,3-d]pyrimidine-2,4(1H,3H)-dione (**14**)

In a 100 mL round-bottom flask, compound **4** (4.31 g, 10 mmol) was agitated with 0.8 mL of chloroacetyl chloride (10 mmol) in 10 mL of ethanol, while catalyst such as tri ethyl amine (five drops) was added. At room temperature, the reaction mixture was agitated for twenty-four hours. After finishing, the solid product was recovered through vacuum filtration, and cold ethanol was used for washing to remove residual impurities. Recrystallization from diethyl ether afforded pure compound **14** as a dark red powder.

Yield 88%; m.p. 298–300 °C; IR (*ν_max_*/cm^−1^): 3410, 3312 (NH_2_), 3050 (CH-arom.), 2929 (CH-Aliph.), 1691, 1648 (2C=O amide), 1595 (C=N pyridine), 1558, 1512 (2C=N triazole), 1491 (C=N thiadiazin), 1453 (C=C arom.); ^1^H-NMR (DMSO-*d*_6_, 400 MHz); *δ* 3.06, 3.15 (s, 6H, 2CH_3_), 4.92 (s, 2H, CH_2_), 7.26–7.41 (m, 4H, Ar-H), 8.60 (s, 2H, NH_2_, D_2_O exchangeable) ppm; ^13^C-NMR (DMSO-*d*_6_, 100 MHz): *δ* 23.07, 26.34 (2C, 2CH_3_), 34.16 (1C, CH_2_ thiadiazine), 102.80 (1C, C pyridine-C=O), 106.00 (1C, C pyridine-triazole), 128.65, 128.85, 128.92, 129.31, 129.83, 130.84 (6C, C-Ar), 150.36 (C=N triazole-pyridine), 151.06 (1C, C=N thiadiazine), 151.08 (1C, C pyridine-Ph-Cl), 154.88, 159.36 (2C, 2C=O uracil), 157.22 (1C, C pyridine-N-CH_3_), 157.50 (1C, C=N pyridine), 162.80 (1C, C=N triazole- thiadiazine) ppm. Anal. Calcd for C_19_H_14_Cl_2_N_8_O_2_S (489.34): C, 46.64; H, 2.88; Cl, 14.49; N, 22.90; O, 6.54; S, 6.55%. Found C, 46.65; H, 3.00; Cl, 14.52; N, 22.91; O, 6.53; S, 6.55%.

#### 3.1.15. 7-Amino-5-(4-chlorophenyl)-6-(6-mercapto-[1,2,4]triazolo[3,4-b][1,3,4]thiadiazol-3-yl)-1,3-dimethylpyrido[2,3-*d*]pyrimidine-2,4(1*H*,3*H*)-dione (**15**)

Compound **4** (4.30 g, 10 mmol) and carbon disulfide (0.6 g, 10 mmol) were combined in 10 mL of pyridine to create compound **15**. The reaction was refluxed for 4 h at 60 °C. After finishing, neutralization with HCl and 50 mL of ice-cold water was performed with cooled mixture. Cold ethanol was used to remove impurities from the precipitate after it had been isolated by vacuum filtering. Compound **15** was refined by recrystallization from ethanol, and the final result was a purple powder.

Yield 65%; m.p. 239–241 °C; IR (*ν_max_*/cm^−1^): 3449, 3276 (NH_2_), 3072, 3049 (CH arom.), 2984, 2940 (CH-Aliph.), 2550 (SH), 1683, 1633 (2 C=O amide), 1584, 1524 (4 C=N), 1432 (C=C arom.); ^1^H-NMR (DMSO-*d*_6_, 400 MHz,): *δ* 3.07, 3.13 (s, 6H, 2CH_3_), 7.15–7.70 (m, 4H, Ar-H), 8.52 (s, 2H, NH_2_, D_2_O exchangeable), 13.24 (s, 1H, SH) ppm; ^13^C-NMR (DMSO-d6, 100 MHz) δ = 23.04, 26.20 (2C, 2CH_3_), 102.00 (1C, C pyridine-C=O), 107.57 (1C, C pyridine-triazole), 128.68, 129.07, 129.74, 131, 131.26, 132.28 (6 C, C-Ar), 152.00 (1C, C=N triazole-pyridine), 152.89 (1C, C pyridine-Ph-Cl), 154.13 (1C, C uracil-N), 154.93, 158.26 (2C, 2C=O uracil), 155.58 (1C, C pyridine-NH2), 166.07 (1C, C=N triazol-S), 188.17 (1C, C=N-SH) ppm; MS: *m*/*z* = 572 [M^+^], 284 (100%). Anal. Calcd for C_18_H_13_ClN_8_O_2_S_2_ (472.93): C, 45.71; H, 2.77; Cl, 7.50; N, 23.69; O, 6.77; S, 13.56%. Found C, 47.71; H, 2.77; Cl, 7.54; N, 23.69; O, 6.78; S, 13.56%.

#### 3.1.16. 7-Amino-5-(4-chlorophenyl)-1,3-dimethyl-*N*-(3-methyl-5-oxo-4,5-dihydro-1*H*-pyrazole-1-carbonothioyl)-2,4-dioxo-1,2,3,4-tetrahydropyrido[2,3-*d*]pyrimidine-6-carboxamide (**16**)

A new methyl pyrazol-3-one derivative **16**, was synthesized by refluxing of compound **6** (4.33 g, 10 mmol) with ethyl acetoacetate (1.3 mL, 10 mmol) in 10 mL of DMF, activation was made by sodium hydride (NaH, 0.24 g, 10 mmol) as a base for 14 h at 100 °C, with thin-layer chromatography (TLC) tracking the development. Following completion, introducing precipitate after cooling was made by adding 50 milliliters of crushed ice. Following vacuum filtration, the solid was collected, and any impurities were removed by washing it with cold ethanol. By recrystallizing ethanol, pure compound **16** was produced as a pale orange powder.

Yield 79%; m p 229–231 °C; IR (*ν_max_*/cm^−1^): 3341, 3194 (NH_2_), 3061 (CH-arom.), 2931, 2839, 2816 (CH-Aliph.), 1727 (C=O pyrazalone), 1703, 1652 (2C=O uracil), 1597 (C=O amide), 1560 (C=N pyridine), 1516 (C=N pyrazalone), 1491 (C=C arom.), 1090 (C=S). ^1^H-NMR (DMSO-*d*_6_, 400 MHz,) *δ* = 1.94 (s, 3H, CH_3_), 3.01 (s, 2H, CH_2_), 3.06, 3.16 (s, 6H, 2CH_3_), 7.14–7.39 (m, 4H, Ar-H), 8.30 (brs, 2H, NH_2_ exchangeable), 12.42 (brs, 1H, NH exchangeable) ppm; ^13^C-NMR (DMSO-d_6_, 100 MHz) *δ* = 16.00 (1C, CH_3_ pyrazalone), 23.32, 26.13 (2C, 2CH_3_), 45.05 (1C, CH_2_), 103.60 (2C, 2C pyridine-C=O), 127.52, 128.07, 129.07, 129.33, 129.84, 131.64 (6C, C-Ar), 151.34 (1C, C pyridine-Ph-Cl), 154.58, 158.89 (2C, C=O uracil), 159.58 (2C, 2C=N pyridine, pyrazole), 161.25 (1C, C pyridine-N-CH_3_), 165.33 (1C, 1C=O pyrazole), 170.06 (1C, C=O amide), 182.25 (1C, C=S) ppm; MS: *m*/*z* = 499 [M^+^], 245 (100%). Anal. Calcd for C_21_H_18_ClN_7_O_4_S (499.93): C, 50.45; H, 3.63; Cl, 7.09; N, 19.61; O, 12.80; S, 6.41%. Found C, 50.48; H, 3.70; Cl, 7.12; N, 19.61; O, 12.82; S, 6.41%.

### 3.2. Biological Activity

The supplementary material included all descriptions of all in vitro investigations.

#### 3.2.1. Assessment of Antiproliferative Activity

Three human malignant cells were acting as evaluating agents of the antitumor efficacy of the newly obtained 1,3-dimethyl-2,4-dioxopyrido[2,3-*d*]pyrimidine -based derivatives **1**–**16** in vitro in order to determine their IC_50_ values.

#### 3.2.2. In Vitro Enzyme Inhibitory Assay Against EGFRWT, Mutant EGFRL858R, and EGFRT790M

The inhibitory effect of newly 1,3-dimethyl-2,4-dioxopyrido[2,3-*d*]pyrimidine derivatives **1** and **2** against EGFR^WT^, mutant EGFR^L858R^, and EGFR^T790M^ was investigated in vitro in comparison to erlotinib.

#### 3.2.3. Cell Cycle and Apoptosis Analysis

Apoptosis research and cell cycle analysis were used on malignant MCF-7 cells for 1,3-dimethyl-2,4-dioxopyrido[2,3-*d*]pyrimidine derivative **1** through flow cytometry.

#### 3.2.4. In Vitro Wound Healing Assessment

The screening procedure and details made use of the wound healing (scratch assay) were provided in the ESI materials.

#### 3.2.5. In Vitro Antibacterial Assessment

Using the agar plate diffusion technique, the inhibition zone widths of 1,3-dimethyl-2,4-dioxopyrido[2,3-*d*]pyrimidine -based derivatives **1**–**16** were examined against a number of species. The two-fold serial dilution method was used to further assess the minimum inhibitory concentration (MIC).

### 3.3. Computational Studies

#### 3.3.1. ADMET In Silico Evaluation

The admetSAR 1.0 free web tools can be used to analyze the ADMET evaluation of the targeted drugs and SwissADME.

#### 3.3.2. Docking Simulation

ChemDraw version 12 was used to draw Compounds **1** and **2**, which were then transformed to 3D and energy-minimized in MOE (v.2024.0601). The Protonate 3D procedure was used to build the EGFR^WT^ and EGFR^T790M^ crystal structures (PDB: 1M17, 3IKA) for docking upon collection from the Protein Data Bank.

Prior to evaluating the compounds in the ATP-binding sites, Triangle Matcher placement and the London dG score function were employed for docking, which was confirmed by re-docking native ligands. The same procedure was used to produce and dock HCV NS5A (PDB: 1AJ6, 3FQM) and *E. coli* DNA gyrase. The Supplementary Information contains all of the experimental details.

#### 3.3.3. Molecular Dynamics Simulation

The supplementary file contains the specifics of the molecular dynamics simulation of 1,3-dimethyluracil derivative **1** with EGFR^WT^ and mutant EGFR^T790M^.

#### 3.3.4. Quantum Chemical Calculation

Understanding molecular characteristics and reaction pathways requires the use of quantum mechanical computations. In this study, density functional theory (DFT) calculations were performed using the 6311G++(d,p) basis set and the B3LYP functional to optimize molecular structures and analyze electronic properties. Gaussian software was used for all computations; more information is given in the associated documentation.

## 4. Conclusions

The outcomes of this study highlight the 7-substituted tetrahydropyrido[2,3-*d*]pyrimidine-6-carboxylates as a versatile chemotype with significant anticancer and antibacterial properties, accompanied by computationally derived antiviral activity predictions. Compound **1**, in particular, demonstrated multi-target activity through EGFR inhibition (wild and mutant forms), apoptosis induction, and antibacterial efficacy. These findings encourage further structural optimization to improve potency and selectivity, alongside evaluation in resistant cancer models and advanced microbial strains. In addition, computational ADMET predictions provided insights into pharmacokinetic and toxicity profiles. The antiviral potential, assessed exclusively via computational approaches, highlights possible avenues for further experimental validation. Collectively, these results support the therapeutic promise of these derivatives in oncology and infectious disease research.

## Figures and Tables

**Table 1:** IC_50_ Inhibitory impact (µM) in vitro of the potentially valuable 1,3-dimethyl uracil **1** and **2** against wild EGFR, mutant EGFR (L858R), and EGFR (T790M), compared with erlotinib and fold-change in potency relative to wild-type EGFR, illustrating resistance impact.

Compd. No.	IC_50_ (Mean ± SD) (µM)			Fold-Change	
	EGFR	EGFR_L858R	EGFR_T790M	L858R/WT	T790M/WT
**1**	0.093 ± 0.003	1.157 ± 0.036	0.174 ± 0.006	12.44×	1.87×
**2**	0.212 ± 0.007	1.603 ± 0.050	0.854 ± 0.027	7.56×	4.03×
**Erlotinib**	0.051 ± 0.002	0.239 ± 0.007	0.098 ± 0.003	4.69×	1.92×

SD reflects variability from three measurements, × expresses fold change, while IC_50_ is defined as the concentration necessary to achieve 50% enzyme inhibition.

**Table 2:** Agar diffusion technique MIC of the most active chemicals.

Compounds	MIC (µg/mL)			
	*E. coli* ATCC25915	*S. aureus* ATCC25923	*E. faecalis* ATCC29212	*P. aeruginosa* ATCC10145
**1**	50	100	100	50
**12**	50	>100	100	50
**13**	50	>100	>100	100
**16**	50	100	100	5

**Table 3:** Physicochemical properties calculations of the promising 1,3-dimethyl-2,4-dioxopyrido[2,3-*d*]pyrimidines **1** and **2**.

Compd.	Violations ^a^	MW ^b^	nHBD ^c^	nHBA ^d^	nRB ^e^	TPSA (Å^2^) ^f^	MLogP ^g^
**Rule**	-	≤500	≤5	≤10	≤10	≤140	≤4.15
**1**	0 (Lipinski & Veber)	388.80	1	5	4	109.21	2.86
**2**	0 (Lipinski & Veber)	374.78	3	5	3	138.03	1.34

^a^ Violations from Lipinski and Veber Rules; ^b^ Molecular Weight; ^c^ Number of Hydrogen Bond Donor; ^d^ Number of Hydrogen Bond Acceptor; ^e^ Number of Rotatable Bond; ^f^ Calculated Lipophilicity (MLog Po/w); ^g^ Topological Polar Surface Area.

**Table 4:** DFT-Based Quantum Chemical Calculations and Reactivity Descriptors of synthesized Compounds.

Cpds.	LUMO	HOMO	ΔE	A	I	X	η	S	ω	ΔN max	ΔN
**1**	−0.168	−0.2744	0.1064	0.168	0.2744	0.2212	0.0532	18.79699	0.919726	2.078947	63.71053
**2**	−0.17313	−0.17791	0.00478	0.17313	0.17791	0.17552	0.00239	418.41	12.89007	36.71967	1427.715
**3**	−0.17124	−0.21647	0.04523	0.17124	0.21647	0.193855	0.022615	44.21844	1.661718	4.285983	150.4786
**4**	−0.18425	−0.24145	0.0572	0.18425	0.24145	0.21285	0.0286	34.96503	1.584095	3.721154	118.6565
**5**	−0.1689	−0.22721	0.05831	0.1689	0.22721	0.198055	0.029155	34.29943	1.345422	3.396587	116.6514
**6**	−0.21131	−0.25065	0.03934	0.21131	0.25065	0.23098	0.01967	50.83884	2.712342	5.871378	172.0646
**7**	−0.16429	−0.20577	0.04148	0.16429	0.20577	0.18503	0.02074	48.21601	1.650728	4.460704	164.2953
**8**	−0.17823	−0.20828	0.03005	0.17823	0.20828	0.193255	0.015025	66.55574	2.48569	6.431115	226.514
**9**	−0.16436	−0.20201	0.03765	0.16436	0.20201	0.183185	0.018825	53.12085	1.782563	4.865471	181.0575
**10**	−0.16012	−0.23905	0.07893	0.16012	0.23905	0.199585	0.039465	25.33891	1.009354	2.528633	86.15754
**11**	−0.16261	−0.19105	0.02844	0.16261	0.19105	0.17683	0.01422	70.32349	2.198935	6.217651	239.9146
**12**	−0.17746	−0.25503	0.07757	0.17746	0.25503	0.216245	0.038785	25.78316	1.20567	2.78774	87.45333
**13**	−0.17528	−0.21691	0.04163	0.17528	0.21691	0.196095	0.020815	48.04228	1.847382	4.710425	163.4375
**14**	−0.18373	−0.21469	0.03096	0.18373	0.21469	0.19921	0.01548	64.59948	2.563606	6.434432	219.6638
**15**	−0.16836	−0.20404	0.03568	0.16836	0.20404	0.1862	0.01784	56.05381	1.94341	5.21861	190.9697
**16**	−0.18343	−0.2266	0.04317	0.18343	0.2266	0.205015	0.021585	46.32847	1.947239	4.749016	157.4006

**Table 5:** Quantum Chemical Descriptors and ESP-Based Composite Bioactivity Prediction of Synthesized Compounds.

Compound	ΔE	S	ω	ESP Score	Composite Bioactivity Score
**1**	0.1064	18.79699	0.919726	0.187937	0.636331
**2**	0.00478	418.41	12.89007	0.870748	0.830612
**3**	0.04523	44.21844	1.661718	0.245007	0.188622
**4**	0.0572	34.96503	1.584095	0.187937	0.637946
**5**	0.05831	34.29943	1.345422	0.378923	1.083423
**6**	0.03934	50.83884	2.712342	0.252531	0.223867
**7**	0.04148	48.21601	1.650728	0.231074	0.175793
**8**	0.03005	66.55574	2.48569	0.215803	0.244654
**9**	0.03765	53.12085	1.782563	0.118538	0.261083
**10**	0.07893	25.33891	1.009354	0.157292	0.169537
**11**	0.02844	70.32349	2.198935	0.5149	0.290725
**12**	0.07757	25.78316	1.20567	0.15	1.066763
**13**	0.04163	48.04228	1.847382	0.217997	0.326359
**14**	0.03096	64.59948	2.563606	0.184459	0.237995
**15**	0.03568	56.05381	1.94341	0.295571	0.275627
**16**	0.04317	46.32847	1.947239	0.272797	0.336711

## Data Availability

The original contributions presented in this study are included in the article. Further inquiries can be directed to the corresponding author.

## Supplementary Materials

The following supporting information can be downloaded at: https://www.mdpi.com/article/10.3390/ph18101472/s1, The following supporting information contain IC50 values of compounds against HeLa, HepG-2, and MCF-7 cell lines, Tables S1–S9, and Figures S1–S56.
