# Supplementary material for: Innovative Amino-Functionalization of Pyrido[2,3-d]pyrimidine Scaffolds for Broad Therapeutic Applications Supported by Computational Analyses
Source: Pharmaceuticals (Basel). 2025 Sep 30;18(10):1472. doi: 10.3390/ph18101472 (PMC12567244; doi:10.3390/ph18101472)
Supplement: Supplementary file 1 [file pharmaceuticals-18-01472-s001.zip › pharmaceuticals-3863184-supplementary.pdf]

Supporting Information

Innovative Amino-Functionalization of Pyrido[2,3-d]pyrimidine Scaffolds for Broad Therapeutic Applications Supported by Computational Analyses

Hagar S. El-Hema [1,*], Haitham E. Shehata [2], Mohamed A. Hawata [2,*], Eman S. Nossier [3,4], Ahmed F. El-Sayed [5,6], Najla A. Altwaijry [7], Asmaa Saleh [7], Modather F. Hussein [8], Amr Sabry [9] and Adel A.-H. Abdel-Rahman [2,*]

[1] Basic Science Department (Chemistry), Thebes Higher Institute for Engineering, Thebes academy, Maadi 11434, Egypt

[2] Chemistry Department, Faculty of Science, Menoufia University, Shebin El-Kom, 32511 Egypt

[3] Pharmaceutical Medicinal Chemistry and Drug Design Department, Faculty of Pharmacy (Girls), Al-Azhar University, Cairo, 11754, Egypt

[4] The National Committee of Drugs, Academy of Scientific Research and Technology, Cairo, 11516, Egypt

[5] Microbial Genetics Department, Biotechnology Research Institute, National Research Centre, Giza, Egypt

[6] Egypt Center for Research and Regenerative Medicine (ECRRM), Cairo, Egypt

[7] Department of Pharmaceutical Sciences, College of Pharmacy, Princess Nourah bint Abdulrahman University, P.O. Box 84428, Riyadh 11671, Saudi

Arabia

[8] Chemistry Department, College of Science, Jouf University, P.O. Box 2014, Sakaka, Aljouf 72341, Saudi Arabia

[9] Department of pharmaceutical manufacturing, College of Pharmaceutical Sciences and Drug Manufacturing, Misr University for Science and Technology (MUST), 6th of October City, P.O. 77, Giza, Egypt

*Correspondance: hagarsabry.23@yahoo.com (H.S.E.-H.); adelnassar63@yahoo.com (A.A.-H.A.-R.); Drmohamedhwata@gmail.com (M.A.-A.H)

Content

- Experimental of chemistry, biological evaluation, docking study, Dynamic stimulations study, Quantum chemical calculations.
- **Table S1 .** The percentage cytotoxicity of the active compounds on human tumor HeLa, HepG-2, MCF-7, and normal WI38 cell lines at different concentrations **.**
- **Table S2.** The cytotoxic effects of 1,3-dimethyl-2,4-dioxopyrido[2,3-*d*]pyrimidines **1–16** on normal WI-38 cell lines and human cancer cell lines HeLa, HepG-2, and MCF-7 that was evaluated at different concentrations using the MTT assay, represented by IC_{50} values and SI selectivity.
- **Table S3.** Detailed results of the enzyme inhibitory assay of 1,3-dimethyl-2,4-dioxopyrido[2,3-*d*]pyrimidines **1** and **2**.
- **Table S4.** Cell cycle analysis after 48 h incubation with compound 1 compared with untreated MCF-7 cells.
- **Table S5.** Apoptosis induction analysis within MCF-7 cells treated with compound **1** compared with untreated MCF-7 cells.
- **Table S6.** Detailed results of wound healing assay of 1,3-dimethyl-2,4-dioxopyrido[2,3-*d*]pyrimidine 1 within MCF-7.
- **Table S7.** Antimicrobial activity of samples evaluated by a well diffusion method.
- **Table S8.** Anticipated ADMET profile of 1,3-dimethyl-2,4-dioxopyrido[2,3-*d*]pyrimidines **1** and **2** using admetSAR 1.0.
- **Table S9.** Visualization of HOMO–LUMO, ESP Maps for synthesized compounds
- **Table S10.** ESP area distribution of compounds **1–16**. Red/Orange/Yellow = Negative ESP regions; Blue/Cyan = Positive ESP regions; Green = Neutral

regions. Values represent the percentage contribution of each surface area to the total molecular ESP map.

- **Table S11.** Electrostatic Potential Surface Maps of synthesized Compounds.
- **Figure S1–S55. Copies** of IR, ^{1}H NMR, ^{13}C NMR, and mass spectra of Compounds.
- **Figure S56**. Antimicrobial activity of samples evaluated by well diffusion method against *S. aureus* ATCC25923, *E. faecalis* ATCC29212 *P. aeruginosa* ATCC10145,and *E. coli* ATCC25915.

3. Experimental

3.1. Chemistry

All melting points are uncorrected and were determined using an Electrothermal IA 9100 apparatus (Shimadzu, Kyoto, Japan). Anti-cancer activity assays were conducted at the Faculty of Pharmacy, Dakahlia, Mansoura University, Egypt. IR spectra were recorded on a JASCO FT/IR 6100 spectrometer (Japan) at Al-Azhar University, Cairo, Egypt, using KBr discs. The ^{1}H and ^{13}CNMR spectra were obtained using a BRUKER 400 MHz and JEOL 500 MHz spectrometers for ^{1}H NMR, and a 100 MHz spectrometer for ^{13}CNMR. These measurements were carried out at the Faculty of Science, Zagazig University, and the Faculty of Science, Mansoura University, Egypt. The spectra were recorded in DMSO-d_6 with tetramethyl silane (TMS, 0.00 ppm) as the internal standard. In the ^{1}HNMR spectra, chemical shifts (δ) are reported along with the number of protons, signal multiplicities (s = singlet, d = doublet, t = triplet, q = quartet, m = multiplet, br = broad), and coupling constants (J) in hertz (Hz). Mass spectrum was carried out using a direct probe controller inlet connected to a single quadrupole mass analyzer in a thermo scientific gcms model (isq lt), using thermo x-calibur software. Reaction progress was monitored by thin-layer chromatography (TLC), performed on Macherey–Nagel aluminum-backed plates pre-coated with silica gel 60 (UV254). All chemicals, solvents, and reagents were obtained from Aldrich Chemical Co., USA, and other commercial suppliers. All solvents were dried before being used. Refer to Supplementary Materials for ^{1}H-NMR and ^{13}C-NMR spectra of sample compounds.

The HeLa, HepG-2, and MCF-7 cell lines were obtained from reputable local sources in Egypt. All cell lines were authenticated and tested negative for mycoplasma contamination. All experiments were conducted in the Research Laboratory, Faculty of Pharmacy, Mansoura University, Egypt. The use of human-

derived cell lines in this study was approved by the Institutional Review Board of Mansoura University, Faculty of Pharmacy (protocol code MU-FP-IRB-2025-01, approved on 10 January 2025). Written informed consent was obtained for all primary human materials used in establishing these cell lines, as applicable.

3.2. Biological evaluation

3.2.1. Antiproliferative activity

The cell lines were purchased from the American Type Culture collection as follows: cervical carcinoma (HeLa), liver carcinoma (HepG-2), breast carcinoma (MCF-7), and normal lung fibroblast cells (WI-38). Cytotoxic activity screening was performed using MTT assay. Exponentially, cells were placed in 10^4 cells/ well for 24 h, and then add fresh medium which containing different concentrations of the tested sample. Serial two-fold dilutions of the tested sample were added using a multichannel pipette. Moreover, all cells were cultivated at 37 °C, 5% CO_2 and 95% humidity. Also, incubation of control cells occurred at 37 °C. However, after incubation for 24 h, different concentrations of samples (100, 50, 25, and 12.5 μM) were added and continued the incubation for 48 h, then, add the crystal violet solution 1% to each well for 0.5 h to examine viable cells. Rinse the wells using water until no stain. After that, add 30% glacial acetic acid to all wells with shaking plates on Microplate reader (TECAN, Inc.) to measure the absorbance, using a test wavelength of 490 nm. Besides, compare the treated samples with the control cell. The cytotoxicity was estimated by IC_{50} in (μM), the concentration that inhibits 50% of growth of cells.

3.2.2. *In vitro* enzyme inhibitory assay against $EGFR^{WT}$, mutant $EGFR^{L858R}$, and $EGFR^{T790M}$

All the compounds were further examined for their inhibitory activities against $EGFR^{WT}$, mutant $EGFR^{L858R}$, and $EGFR^{T790M}$. Homogeneous time resolved fluorescence (HTRF) assay was applied in this test with $EGFR^{WT}$, mutant $EGFR^{L858R}$, and $EGFR^{T790M}$

(Sigma). Firstly, $EGFR^{WT}$, mutant $EGFR^{L858R}$, and $EGFR^{T790M}$ and their substrates were incubated with the tested compounds in the enzymatic buffer for 5 min. ATP (1.65 μM) was added to the reaction mixture to allow the enzymatic reaction. The assay was conducted for 30 min at room temperature. The reaction was stopped by the addition of detection reagents which contain EDTA. The detection step continued for 1 h, and then the IC_{50} values were determined using GraphPad Prism 5.0. Three independent experiments were performed for each concentration.

3.2.3. Cell cycle arrest and apoptosis of compound 1

Cell cycle analysis and apoptosis study were carried out using flow cytometry. MCF-7 cells were seeded at 8×10^4 and incubated at 37°C in 5% CO_2 overnight. After treatment with the tested compound **1** for 24 h, cell pellets were collected and centrifuged (300 g, 5 min). For cell cycle analysis cell pellets were fixed with 70% ethanol on ice for 15 min and collected again. The pellets were incubated with propidium iodide (PI) staining solution at room temperature for 1 h and analyzed by a Gallios flow cytometer (Beckman Coulter, Brea, CA, USA). Apoptosis detection was carried out by FITC AnnexinV/PI commercial kit (Becton Dickenson, Franklin Lakes, NJ, USA) following the manufacturer protocol. The samples were analyzed by fluorescence-activated cell sorting (FACS) with a Gallios flow cytometer (Beckman Coulter, Brea, CA, USA) within 1 h after staining. Data were analyzed using Kaluza v 1.2 (Beckman Coulter).

3.2.4. Wound healing assay

The anti-migratory effect of the compound was assessed using MCF-7 cells. After seeding 8×10^5 cells per well in a six-well plate and allowing them to adhere, a straight wound was created in the confluent cell monolayer using a 200 μL pipette tip. The wells were gently rinsed three times with sterile PBS to eliminate non-adherent cells, followed by the addition of 2 mL fresh medium. The initial wound width (0 h) was documented using an inverted microscope. The medium was then replaced with fresh medium containing varying concentrations of compound **11c**,

and the cells were cultured for 24 h. After incubation, the scratch area was re-examined under the microscope to evaluate migratory changes.

3.2.5. Antimicrobial activity assay

Diameter of the inhibition zones

The biological potential of the newly prepared 1,3-dimethyl-2,4-dioxopyrido[2,3-*d*]pyrimidines **1–16** were inspected toward the examined organisms and expressed as the diameter of the inhibition zones due to the agar plate diffusion technique. The tested microorganisms were used in this study, against four bacterial strains *Escherichia coli* (ATCC 25915), *Staphylococcus aureus* (ATCC 25923), *Enterococcus faecalis* (ATCC 29212), and *Pseudomonas aeruginosa* (ATCC 10145). The pathological strains (100 µl) were outgrowing in 10 mL of fresh media till they reached a count of nearly 108 cells/ml for bacteria. Also, each well (10 mm diameter holes cut in the agar gel) included 1mL of each sample (at 0.5 mg/mL). Whoever, incubation of plates was done for 24 h at 37 ˚C for bacteria and 72 h at 27˚C for fungi activity. The plates were done in triplicate and the average inhibition zone diameters were recorded in mm and used as criterion for the microbial activity. DMSO (solvent controls) was used for dissolving the examined compounds and illustrated no inhibition zone, indicating that it has no effect on the growth of the tested biological strains. Furthermore, the proper target compounds were further tested to estimate their antimicrobial activity represented as minimum inhibitory concentration (MIC) using the modified agar well diffusion method. Ciprofloxacin was used as a standard antibacterial drug.

Minimal Inhibitory Concentration (MIC) Measurement

The bacteriostatic activity of the compounds was then evaluated using the two-fold serial dilution technique. Two-fold serial dilutions of the tested compounds' solutions were prepared using the proper nutrient broth. The final concentrations of the solutions were 100, 50, and 25 µg/mL. The tubes were then inoculated with the test organisms, grown in their suitable broth at 37°C for 24 hours for the tested

microorganisms (1×10^8 CFU/mL for bacteria), each 5 mL received 0.1 mL of the above inoculum and incubated at 37°C for 24 h. The lowest concentration showing no growth was taken as the minimum inhibitory concentration (MIC).

4.1. Molecular docking study

The 2D structures of 1,3-dimethyl-2,4-dioxopyrido[2,3-*d*]pyrimidine derivatives **1** and **2** were drawn through Chem. Draw. The protonated 3D was employed using standard bond lengths and angles, using Molecular Operating Environment (MOE-Dock) software version 2024.0601. Then, the geometry optimization and energy minimization were applied to get the Conf Search module in MOE, followed by saving of the moe file for upcoming docking process. The co-crystallized structures of $EGFR^{WT}$, and mutant $EGFR^{T790M}$ with their ligands erlotinib and WZ4002 were downloaded (PDB codes: 1M17 and 3IKA, respectively) from protein data bank. All minimizations were performed using MOE until an RMSD gradient of 0.05 $kcal{\cdot}mol^{-1}Å^{-1}$ with MMFF94x force field and the partial charges were automatically calculated. Preparation of the enzymes' structures were done for molecular docking using Protonate 3D protocol with the default options in MOE. London dG scoring function and Triangle Matcher placement method were used in the docking protocol. Initially, the validation of the docking processes was established by docking the native ligands, followed by docking the derivatives **1** and **2** within the ATP-binding sites after eliminating the co-crystallized ligands.

For confirmation of the antimicrobial and antiviral activity, the crystal structures of *E. coli* DNA gyrase and HCV NS5A were obtained from the RCSB database (PDB codes: 1AJ6 & 3FQM, respectively), following the previously mentioned method to complete the docking processes of the promising compound **1**.

4.2. Molecular Dynamic Simulations

Molecular dynamic simulations were executed, spanning a duration of 100 ns, employing the Desmond software package developed by Schrödinger LLC. These simulations utilized computational resources hosted on the Smart Encoders website. The initial configurations of protein–ligand complexes for the molecular dynamics simulations were sourced from preceding docking studies, which offer predictions of ligand binding behavior under static conditions. Subsequent simulations were conducted to forecast how ligand binding would occur within a physiological context.

The protein–ligand complexes underwent initial processing using either the Protein Preparation Wizard or Maestro, incorporating optimization and minimization steps for complex refinement. The assembly of all systems was facilitated through the System Builder tool. A solvent model featuring an orthorhombic box, specifically the Transferable Intermolecular Interaction Potential 3 Points model, was chosen. The OPLS 2005 force field was applied for the ensuing simulations. Neutralization of models was achieved by introducing counter ions where necessary.

To emulate physiological conditions, a concentration of 0.15 M salt (NaCl) was introduced. For the complete simulation, the moles, pressure, temperature conservation (NPT) ensemble was selected, maintaining a temperature of 300 K and a pressure of 1 atm. Prior to the simulation, models underwent relaxation. Trajectories were saved at 50 ps intervals for subsequent analysis, and the stability of the simulations was assessed by calculating the RMSD of both the protein and the ligand as a function of time.

4.3. Quantum Chemical Calculations

Density Functional Theory (DFT) computations were performed using the B3LYP functional, a hybrid exchange-correlation functional that combines the gradient-corrected correlation functional of Lee, Yang, and Parr (LYP) with Becke's three-parameter exchange functional. By addressing integration concerns, this method has advantages over pure DFT techniques. Gaussian 09 was used to fully optimize all molecular geometries at the B3LYP/6-311G++(d,p) level of theory. The energy gap (ΔE), global electrophilicity (ω), softness (σ), electronegativity (χ), hardness (η), and ionization potential (I) were then determined using frontier molecular orbital (FMO) analysis. Using the optimized geometries, calculations were carried out at the B3LYP/6-311G++(d,p) level of theory in order to visualize the molecular electrostatic potential (MEP). The MEP maps reveal information about the molecules' electrophilic and nucleophilic areas

Tables

Table S1. The percentage cytotoxicity of the active compounds on human tumor HeLa, HepG-2, MCF-7, and normal WI38 cell lines at different concentrations.

Conc.(μM)	WI38	Hela	HepG-2	MCF-7
Erlotinib				
1.56	47.4	7.9	9.2	8.1
3.125	58.5	18.1	16.6	15.2
6.25	72.6	23.9	25.7	23.0
12.5	84.7	34.6	37.2	34.8
25	98.2	56.5	61.3	51.6
50	100	72.1	72.9	68.9
100	100	87.7	95.4	87.5
1				
1.56	47.9	7.7	7.2	4.9

3.125	60.4	14.5	11.9	10.2
6.25	72.9	20.8	20.3	16.5
12.5	87.2	31.9	39.4	23.4
25	98.6	45.2	50.8	41.6
50	100	65.6	61.6	58.1
100	100	88.4	73.4	67.3
2				
1.56	43.9	8.2	13.7	8.7
3.125	55.1	16.9	22.6	16.8
6.25	62.9	24.0	30.9	28.8
12.5	84.2	32.5	46.8	34.6
25	95.3	54.0	65.1	60.4
50	100	70.4	87.2	70.3
100	100	88.3	99.3	89.2
3				
1.56	38.1	16.7	25.1	20.5
3.125	50.9	27.6	31.8	31.8
6.25	62.7	33.3	45.6	42.6
12.5	78.7	45.2	57.2	54.2
25	96.5	68.6	70.3	71.7
50	100	85.5	91.4	92.3
100	100	100	100	100
4				
1.56	34.2	23.4	23.7	26.8
3.125	49.5	30.9	37.9	37.1
6.25	59.4	44.2	49.1	50.7
12.5	74.1	52.4	53.8	58.9
25	96.5	65.6	70.6	74.4
50	100	91.6	92.3	93.9
100	100	100	100	100
5				
1.56	27.9	49.8	56.9	49.2
3.125	41.9	63.1	69.7	60.1
6.25	56.2	75.9	82.4	72.9
12.5	71.8	91.4	95.3	85.2
25	83.9	100	100	98.4
50	100	100	100	100
100	100	100	100	100

6				
1.56	31.6	23.8	24.8	29.8
3.125	40.9	38.5	39.1	40.9
6.25	61.2	50.1	51.7	52.9
12.5	70.5	54.7	68.5	65.4
25	86.3	76.2	79.0	78.9
50	99.7	94.9	97.6	98.7
100	100	100	100	100
7				
1.56	26.8	7.9	20.3	12.9
3.125	37.1	19.3	27.8	21.8
6.25	50.5	24.4	38.7	29.7
12.5	61.5	38.5	51.4	40.3
25	75.4	57.3	73.6	67.2
50	93.9	84.4	91.4	81.4
100	100	97.6	100	98.1
8				
1.56	23.8	45.9	46.4	48.6
3.125	35.7	56.7	61.6	59.1
6.25	50.6	68.3	79.5	68.7
12.5	63.5	84.6	86.1	89.3
25	71.8	99.2	98.2	99.4
50	90.9	100	100	100
100	100	100	100	100
9				
1.56	53.4	42.9	47.8	42.9
3.125	77.8	54.1	65.7	57.1
6.25	83.4	66.5	76.9	69.3
12.5	99.6	78.4	90.2	82.1
25	100	93.8	100	95.6
50	100	100	100	100
100	100	100	100	100
10				
1.56	29.9	36.2	44.3	37.7
3.125	44.5	48.1	56.1	53.1
6.25	58.8	63.2	70.5	62.5
12.5	64.4	75.7	83.6	74.3
25	95.3	96.0	97.4	96.7

50	100	100	100	100
100	100	100	100	100
11				
1.56	52.8	41.3	45.5	38.6
3.125	73.4	49.8	58.0	54.5
6.25	89.7	65.7	70.3	69.0
12.5	95.6	78.6	83.4	88.4
25	100	92.1	98.0	100
50	100	100	100	100
100	100	100	100	100
12				
1.56	21.4	34.7	38.5	41.8
3.125	30.9	45.0	53.1	53.1
6.25	41.2	60.1	64.9	64.2
12.5	50.3	76.5	71.4	77.0
25	70.9	94.2	92.3	92.6
50	91.6	100	100	100
100	100	100	100	100
13				
1.56	13.1	26.8	30.4	31.3
3.125	17.8	35.7	41.6	41.8
6.25	29.0	50.6	53.1	52.7
12.5	40.3	67.1	65.5	73.4
25	63.7	82.0	79.2	88.2
50	78.6	95.3	98.3	99.6
100	96.5	100	100	100
14				
1.56	24.1	42.7	50.8	46.3
3.125	35.0	58.3	64.7	52.3
6.25	46.7	69.1	76.5	89.8
12.5	57.3	84.8	92.6	97.3
25	71.8	99.2	100	100
50	90.5	100	100	100
100	100	100	100	100
15				
1.56	34.6	33.6	38.6	36.7
3.125	44.8	45.1	49.1	49.1
6.25	58.1	56.0	62.7	60.3

12.5	69.3	69.2	78.5	74.5
25	91.4	91.3	96.0	87.2
50	100	100	100	100
100	100	100	100	100
16				
1.56	36.5	30.4	42.9	35.4
3.125	55.4	42.5	55.7	49.1
6.25	68.8	53.6	67.2	56.3
12.5	78.0	66.7	80.4	69.9
25	93.7	87.9	94.7	83.8
50	100	99.6	100	98.5
100	100	100	100	100

Table S2. The cytotoxic effects of 1,3-dimethyl-2,4-dioxopyrido[2,3-*d*]pyrimidines **1–16** on normal WI-38 cell lines and human cancer cell lines HeLa, HepG-2, and MCF-7 that was evaluated at different concentrations using the MTT assay, represented by IC_{50} values and SI selectivity.

Compd. No.	HeLa	SI HeLa	HepG-2	SI HepG-2	MCF-7	SI MCF-7	WI-38
**1**	6.29±0.5	12.98	5.90±0.3	13.83	3.98±0.2	20.51	81.65±4.1
**2**	7.54±0.7	8.6	12.94±1.0	4.95	8.61±0.6	7.44	64.07±3.5
**3**	14.12±1.2	3.73	21.41±1.5	2.46	19.50±1.3	2.70	52.76±2.9
**4**	18.91±1.4	2.43	23.06±1.6	1.99	26.05±1.7	1.76	46.09±2.7
**5**	89.43±4.6	0.39	>100	-	84.08±4.2	0.41	35.22±2.2
**6**	24.83±1.6	1.54	29.73±1.8	1.29	31.96±1.9	1.20	38.39±2.6
**7**	9.72±0.9	2.75	17.52±1.3	1.52	11.62±0.9	2.30	26.76±1.8
**8**	71.38±3.8	0.35	82.75±4.1	0.30	79.33±3.9	0.31	25.20±1.8
**9**	62.73±3.5	-	88.60±4.4	-	67.80±3.6	-	>100
**10**	48.68±2.8	0.77	69.43±3.7	0.54	53.11±3.1	0.71	37.93±2.4
**11**	56.35±3.2	-	73.23±3.8	-	60.63±3.5	-	>100

**12**	44.21±2.6	0.41	54.26±3.2	0.33	58.80±3.3	0.31	18.24±1.4
**13**	28.67±1.9	0.37	32.65±2.1	0.32	36.15±2.2	0.29	10.65±0.8
**14**	68.70±3.6	0.32	93.74±4.8	0.23	75.08±3.8	0.29	22.49±1.6
**15**	39.79±2.4	1.03	51.75±2.9	0.79	47.46±2.8	0.86	41.17±2.5
**16**	34.77±2.1	1.65	65.06±3.4	0.88	42.86±2.5	1.34	57.61±3.2
**Erlotinib**	8.04±0.5	9.74	9.18±0.6	8.53	7.26±0.3	10.78	78.32±3.9

The standard deviation, or SD, is the mean of three measurements, while the IC50 is the concentration of a substance required to impede development by 50%. SI (Selectivity Index) was calculated as the ratio of IC_{50} for the normal cell line (WI-38) to IC_{50} for the corresponding cancer cell line. Higher SI values indicate greater selectivity toward cancer cells.

Table S3. Detailed results of the enzyme inhibitory assay of 1,3-dimethyl-2,4-dioxopyrido[2,3-*d*]pyrimidines **1** and **2**.

<u>EGFR WT</u>

EGFR

code	IC50	conc	log	%inh	T2	T1	ΔT	RFU2	RFU1	ΔRFU	slope	K.Activity
**1**		100	2	96.1	30	0	30	2339	0	2339	1982	4.7205
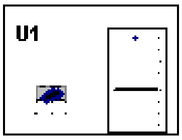		10	1	85.3	30	0	30	8762	0	8762	1982	17.683
		1	0	74.5	30	0	30	15171	0	15171	1982	30.618
		0.1	-1	51	30	0	30	29151	0	29151	1982	58.831
		0.01	-2	29.6	30	0	30	41872	0	41872	1982	84.505
EC				0	30	0	30	59461	0	59461	1982	120

code	IC50	conc	log	%inh	T2	T1	ΔT	RFU2	RFU1	ΔRFU	slope	K.Activity
**2**		100	2	94.6	30	0	30	3186	0	3186	1982	6.4299
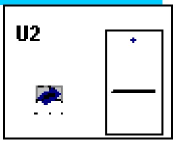		10	1	81.4	30	0	30	11072	0	11072	1982	22.345
		1	0	67.1	30	0	30	19584	0	19584	1982	39.524
		0.1	-1	42.5	30	0	30	34169	0	34169	1982	68.959
		0.01	-2	24.6	30	0	30	44828	0	44828	1982	90.47

EC				0	30	0	30	57139	0	57139	1905	120
	[redacted]		[redacted]		[redacted]		[redacted]		[redacted]			[redacted]
code	IC50	conc	log	%inh	T2	T1	ΔT	RFU2	RFU1	ΔRFU	slope	K.Activity
Erlotinib		100	2	96.9	30	0	30	1841	0	1841	1982	3.7154
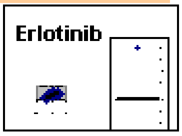		10	1	90	30	0	30	5932	0	5932	1982	11.972
		1	0	76.3	30	0	30	14066	0	14066	1982	28.387
		0.1	-1	57.6	30	0	30	25186	0	25186	1982	50.829
		0.01	-2	32.8	30	0	30	39976	0	39976	1982	80.678
EC				0	30	0	30	57139	0	57139	1905	120
	[redacted]		[redacted]		[redacted]		[redacted]		[redacted]			[redacted]

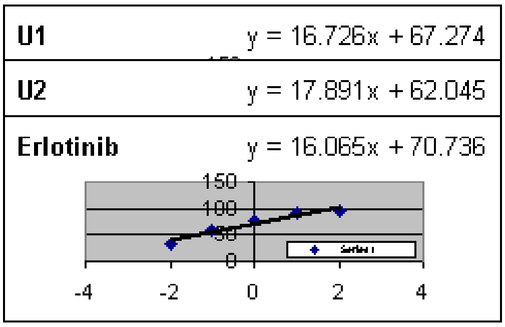

EGFR L858R

**L858R**			[redacted]									
code	IC50	conc	log	%inh	T2	T1	ΔT	RFU2	RFU1	ΔRFU	slope	K.Activity
**1**		100	2	93.7	30	0	30	2976	0	2976	1586	7.5057
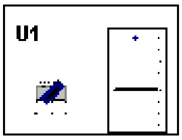		10	1	75.6	30	0	30	11587	0	11587	1586	29.223
		1	0	40	30	0	30	28538	0	28538	1586	71.975
		0.1	-1	23.7	30	0	30	36284	0	36284	1586	91.511
		0.01	-2	9.88	30	0	30	42881	0	42881	1586	108.15
EC				0	30	0	30	47582	0	47582	1586	120
	[redacted]		[redacted]		[redacted]		[redacted]		[redacted]			[redacted]
code	IC50	conc	log	%inh	T2	T1	ΔT	RFU2	RFU1	ΔRFU	slope	K.Activity
**2**		100	2	90.7	30	0	30	4429	0	4429	1586	11.17
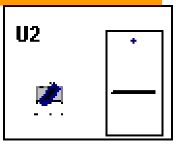		10	1	68.3	30	0	30	15061	0	15061	1586	37.985

code	IC50	conc	log	%inh	T2	T1	ΔT	RFU2	RFU1	ΔRFU	slope	K.Activity
		1	0	43.5	30	0	30	26879	0	26879	1586	67.791
		0.1	-1	19	30	0	30	38541	0	38541	1586	97.203
		0.01	-2	6.07	30	0	30	44691	0	44691	1586	112.71
EC				0	30	0	30	47582	0	47582	1586	120
	[redacted]		[redacted]		[redacted]		[redacted]		[redacted]	[redacted]		[redacted]

code	IC50	conc	log	%inh	T2	T1	ΔT	RFU2	RFU1	ΔRFU	slope	K.Activity
Erlotinib		100	2	95.5	30	0	30	2154	0	2154	1586	5.4325
Erlotinib 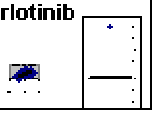		10	1	81.3	30	0	30	8876	0	8876	1586	22.386
		1	0	67.4	30	0	30	15493	0	15493	1586	39.074
		0.1	-1	41.2	30	0	30	27999	0	27999	1586	70.615
		0.01	-2	22.5	30	0	30	36875	0	36875	1586	93.001
EC				0	30	0	30	47582	0	47582	1586	120
	[redacted]		[redacted]		[redacted]		[redacted]		[redacted]	[redacted]		[redacted]

U1	y = 21.964x + 48.606
U2	y = 21.859x + 45.523
Erlotinib	y = 18.614x + 61.582

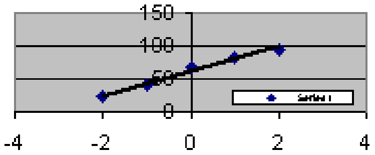

EGFR_T790M

T790M		[redacted]										
code	IC50	conc	log	%inh	T2	T1	ΔT	RFU2	RFU1	ΔRFU	slope	K.Activity
1		100	2	96.1	30	0	30	2052	0	2052	1763	4.6557
U1 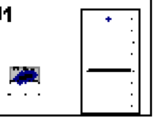		10	1	82.2	30	0	30	9436	0	9436	1763	21.409
		1	0	66.5	30	0	30	17692	0	17692	1763	40.141
		0.1	-1	51.1	30	0	30	25882	0	25882	1763	58.723
		0.01	-2	22.1	30	0	30	41217	0	41217	1763	93.516
EC				0	30	0	30	52886	0	52886	1763	120
	[redacted]		[redacted]		[redacted]		[redacted]		[redacted]	[redacted]		[redacted]

code	IC50	conc	log	%inh	T2	T1	ΔT	RFU2	RFU1	ΔRFU	slope	K.Activity
**12**		100	2	93.5	30	0	30	3418	0	3418	1763	7.755
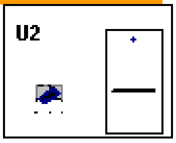		10	1	75.1	30	0	30	13155	0	13155	1763	29.847
		1	0	52.9	30	0	30	24928	0	24928	1763	56.558
		0.1	-1	29.8	30	0	30	37113	0	37113	1763	84.204
		0.01	-2	6.2	30	0	30	49612	0	49612	1763	112.56
EC				0	30	0	30	52886	0	52886	1763	120
	[redacted]		[redacted]		[redacted]		[redacted]		[redacted]			[redacted]

code	IC50	conc	log	%inh	T2	T1	ΔT	RFU2	RFU1	ΔRFU	slope	K.Activity
Erlotinib		100	2	97	30	0	30	1586	0	1586	1763	3.5984
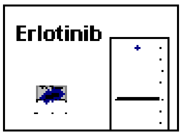		10	1	85	30	0	30	7928	0	7928	1763	17.988
		1	0	69.1	30	0	30	16318	0	16318	1763	37.023
		0.1	-1	51.3	30	0	30	25776	0	25776	1763	58.482
		0.01	-2	31	30	0	30	36492	0	36492	1763	82.795
EC				0	30	0	30	52886	0	52886	1763	120
	[redacted]		[redacted]		[redacted]		[redacted]		[redacted]			[redacted]

U1 $y = 17.919x + 63.593$

U2 $y = 21.998x + 51.512$

Erlotinib $y = 16.574x + 66.686$

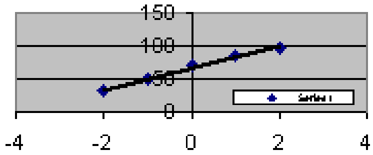

Table S4. Cell cycle analysis after 48 h incubation with compound 1 compared with untreated MCF-7 cells.

Compound No.	%G0-G1	%S	%G2/M
**1 /MCF-7**	74.86	22.79	1.75
**Cont./MCF-7**	56.29	35.72	7.99

Table S5. Apoptosis induction analysis within MCF-7 cells treated with compound **1** compared with untreated MCF-7 cells.

	Apoptosis			Necrosis
	**Total**	**Early**	**Late**	
**1 /MCF-7**	26.37	5.86	16.62	3.89
**Cont. /MCF-7**	3.18	0.64	0.23	2.31

Table S6. Detailed results of wound healing assay of 1,3-dimethyl-2,4-dioxopyrido[2,3-*d*]pyrimidine 1 within MCF-7.

Compound No.	% Closure
**1 /MCF-7**	69.63±2.57
**Cont./MCF-7**	94.815±3.5

	% Closure	Total area	Migrated cells area	T	Length	L.of migration	L1	L2	L3
				72h	mm	ΔL			
**U1/MCF7**	69.63	0.81	0.564		0.9	0.31	0.31	0.32	0.31
**cont. /MCF7**	94.815	0.81	0.768		0.9	0.43	0.42	0.43	0.43

Table S7. Antimicrobial activity of samples evaluated by a well diffusion method.

Compounds (100 µg/mL)	Inhibition zone (mm)			
	S. aureus ATCC25923	*E. faecalis* ATCC29212	*E.coli* ATCC25915	*P. aeruginosa* ATCC10145
	**G + ve**	**G + ve**	**G - ve**	**G - ve**
**DMSO**	(-)	(-)	(-)	(-)
**1**	(-)	(+) 2.0±0.05	(+) 9.50±0.12	(+) 10.50±0.05
**2**	(-)	(-)	(-)	(-)
**3**	(-)	(-)	(-)	(-)
**4**	(-)	(-)	(-)	(+) 6.00±0.00
**5**	(-)	(-)	(-)	(-)
**6**	(-)	(-)	(-)	(-)
**7**	(-)	(-)	(-)	(-)
**8**	(-)	(-)	(-)	(-)
**9**	(-)	(-)	(-)	(+) 7.60±0.00
**10**	(-)	(-)	(-)	(+) 6.0±0.00
**11**	(-)	(-)	(+) 2.0±0.10	(+) 8.60±0.00
**12**	(-)	(+) 2.0±0.05	(+) 10.40±0.08	(+) 10.50±0.00
**13**	(-)	(-)	(+) 14.50±0.15	(+) 16.80±0.00
**14**	(+) 3.0±0.05	(-)	(+) 8.15±0.30	(+) 9.50±0.00
**15**	(-)	(-)	(+) 7.50±0.15	(+) 10.30±0.00
**16**	(+) 4.50±0.10	(+) 4.00±0.60	(+) 18.50±0.15	(+) 17.60±0.00
**Ciprofloxacin** (50µg/mL)	(+) 8.00 ± 0.15	(+) 7.50 ± 0.00	(+) 10.00 ± 0.00	(+) 12.30 ± 0.40

The experiment was carried out in triplicate. Values are given as mean ± standard error. (G + ve) Gram-Positive, (G – ve) Gram-Negative, and (−) Negative result.

Table S8. Anticipated ADMET profile of 1,3-dimethyl-2,4-dioxopyrido[2,3-*d*]pyrimidines **1** and **2** using admetSAR 1.0.

Properties (Probability)	Compound	
	**1**	**2**
**Absorption**		
BBB	NO	NO
HIA	High	High
P-glycoprotein Substrate	Non-substrate (0.7201)	Non-substrate (0.7246)
**Distribution**		

Subcellular localization	Lysosome (0.5721)	Mitochondria (0.5731)
**Metabolism**		
CYP450 2C9 Substrate	Non-substrate (0.8159)	Non-substrate (0.8063)
CYP450 2D6 Substrate	Non-substrate (0.8580)	Non-substrate (0.8563)
CYP450 3A4 Substrate	Substrate (0.5635)	Substrate (0.5115)
CYP450 1A2 Inhibitor	Non-inhibitor (0.7394)	Non-inhibitor (0.7604)
CYP450 2C9 Inhibitor	Inhibitor (0.6289)	Non-inhibitor (0.6732)
CYP450 2D6 Inhibitor	Non-inhibitor (0.9144)	Non-inhibitor (0.9382)
CYP450 2C19 Inhibitor	Non-inhibitor (0.5074)	Non-inhibitor (0.7850)
CYP450 3A4 Inhibitor	Non-inhibitor (0.9313)	Non-inhibitor (0.7407)
**Excretion & Toxicity**		
**hERG Inhibition** T_hERG_I	Weak inhibitor (0.9784)	Weak inhibitor (0.9301)
T_hERG_II	Non-inhibitor (0.7368)	Non-inhibitor (0.6685)
**AMES Toxicity**	Non-AMES toxic (0.6509)	Non-AMES toxic (0.6061)
**Carcinogens**	Non-carcinogens (0.7669)	Non-carcinogens (0.7291)
**Acute Oral Toxicity (AO)**	III (0.7711)	III (0.7284)
**Carcinogenicity**	non-	non-required (0.5715)

**(Three-class)**	required (0.5320)	
**Biodegradation**	Not ready biodegradable (0.9859)	Not ready biodegradable (1.0000)

Table S9. Visualization of HOMO–LUMO, ESP Maps for synthesized compounds.

Compound	Lumo	Homo	ESP	ELF
1	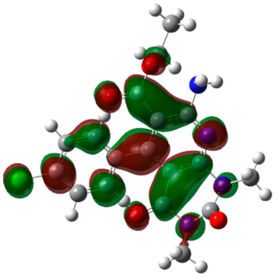	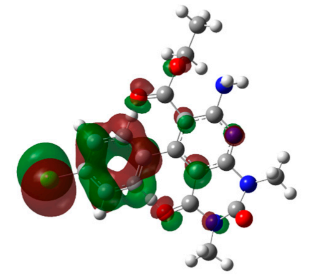	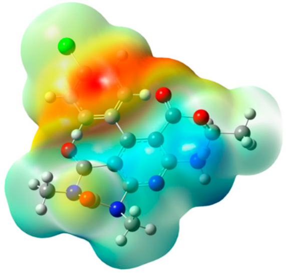	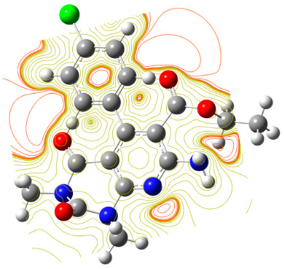
2	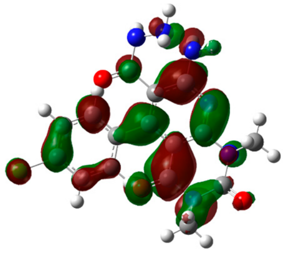	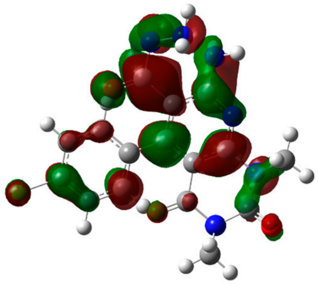	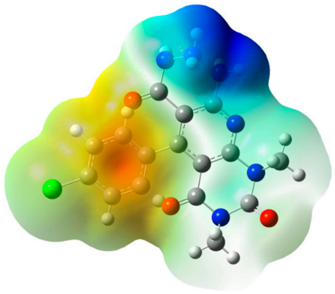	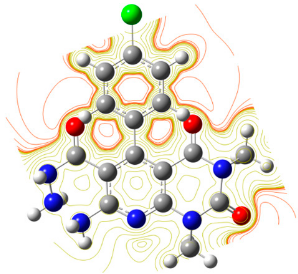
3	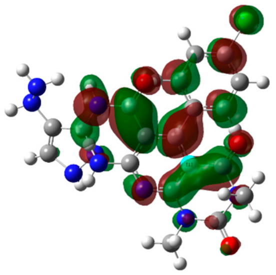	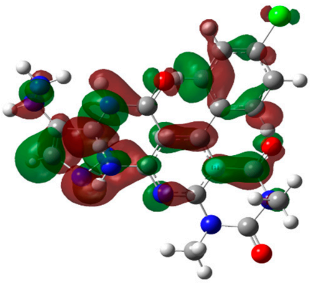	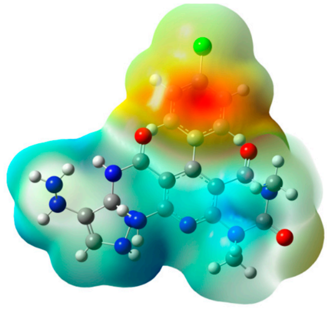	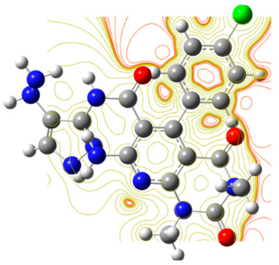
4	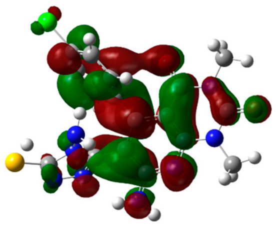	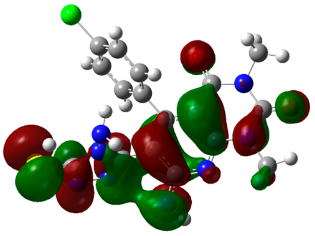	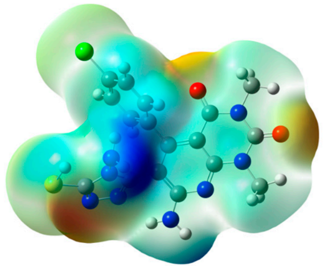	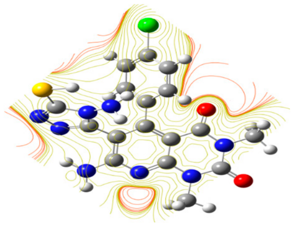

5

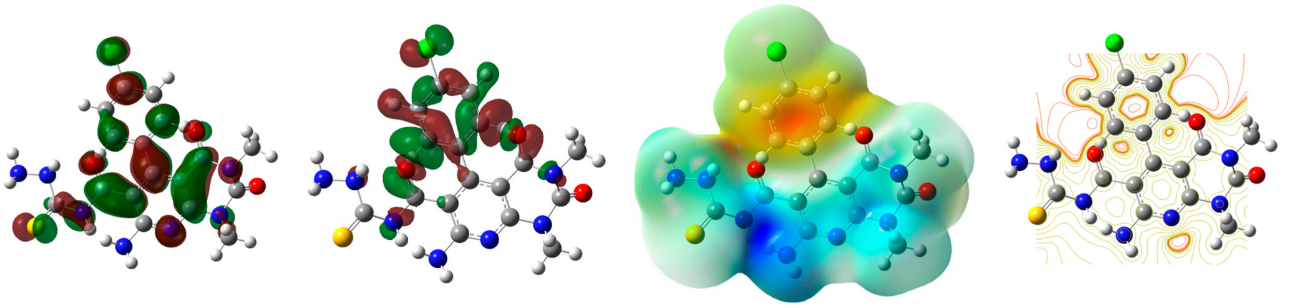

6

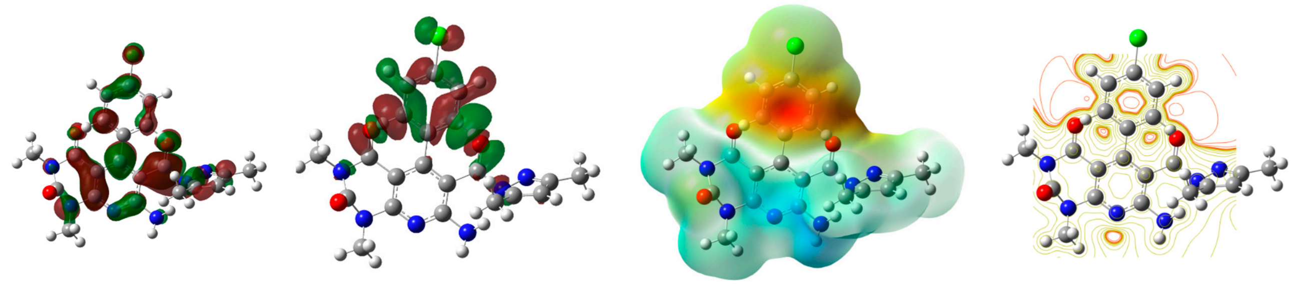

7

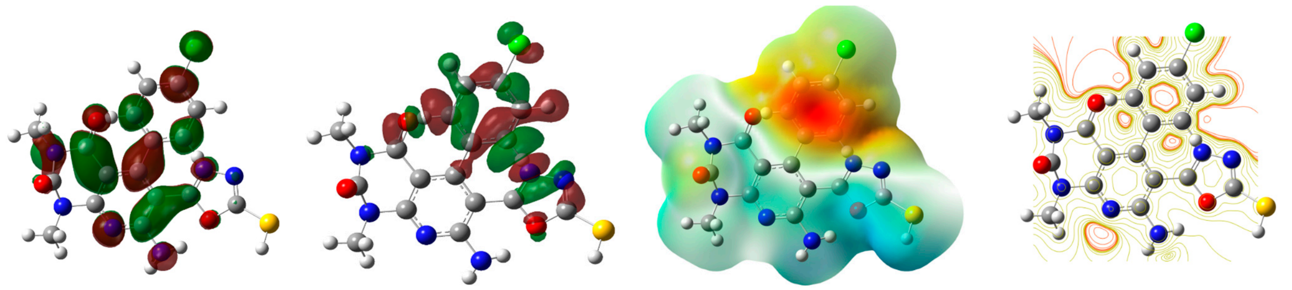

8

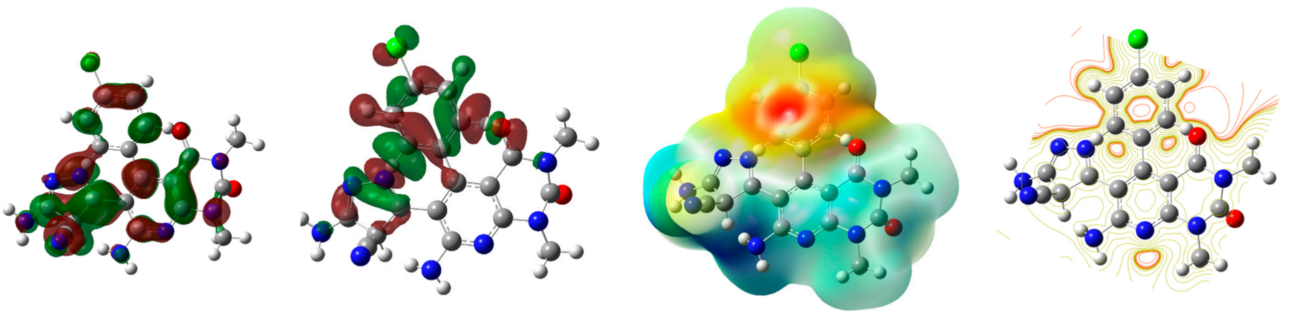

9

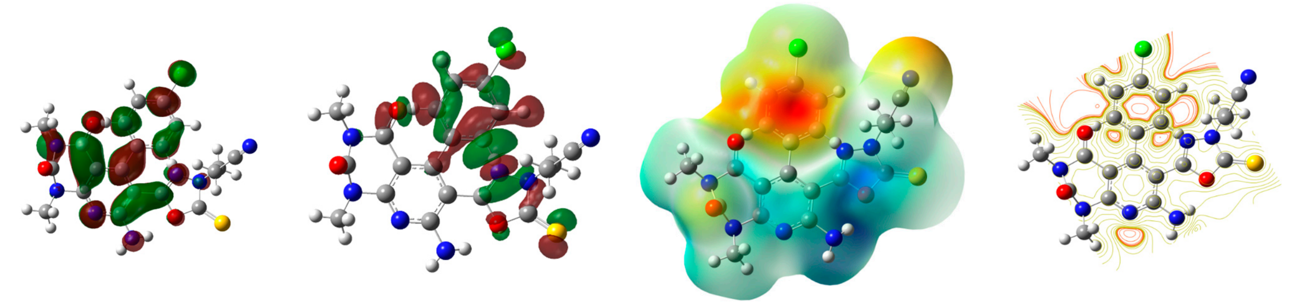

10

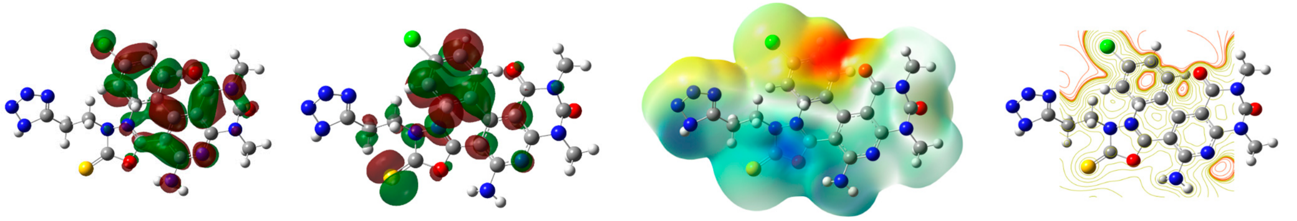

11

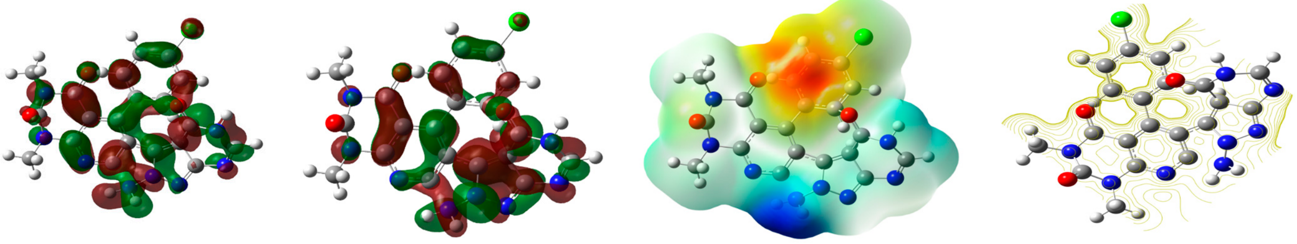

12

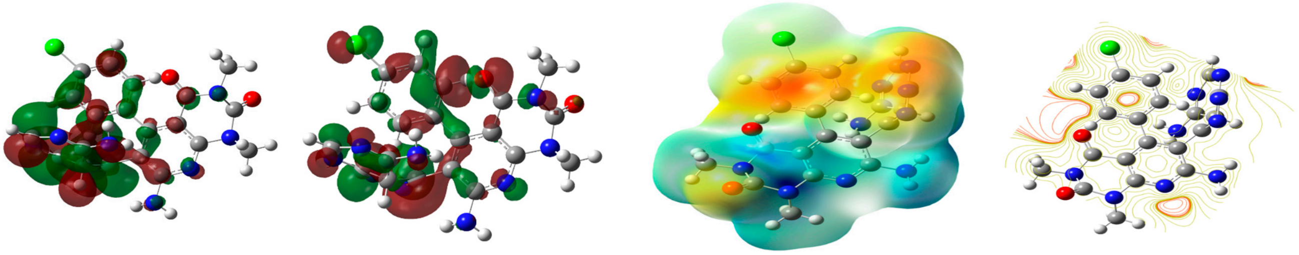

13

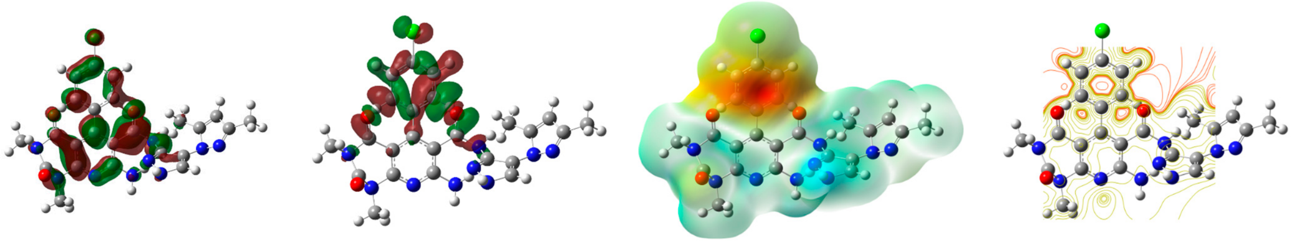

14

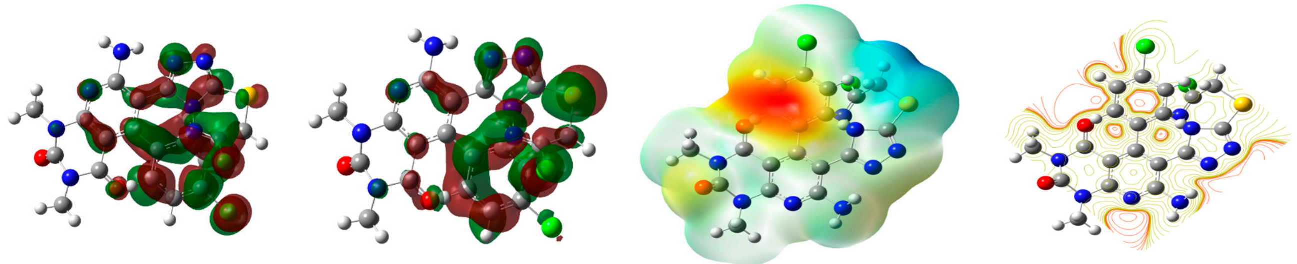

15

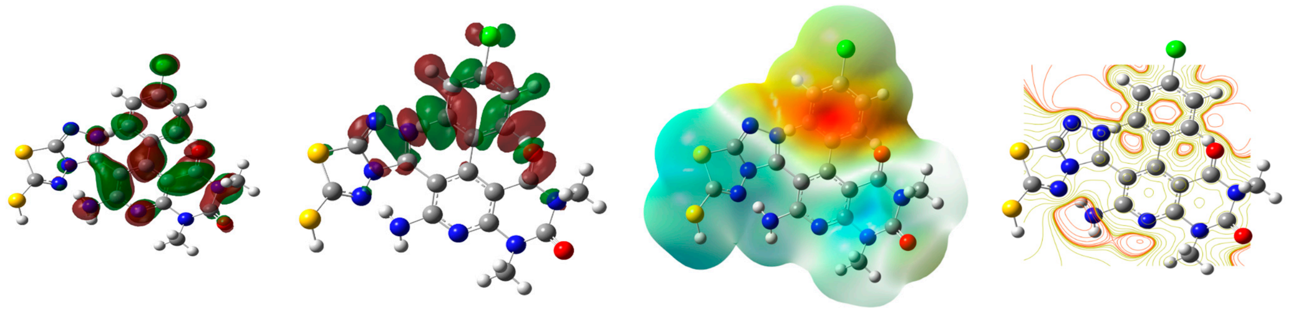

16

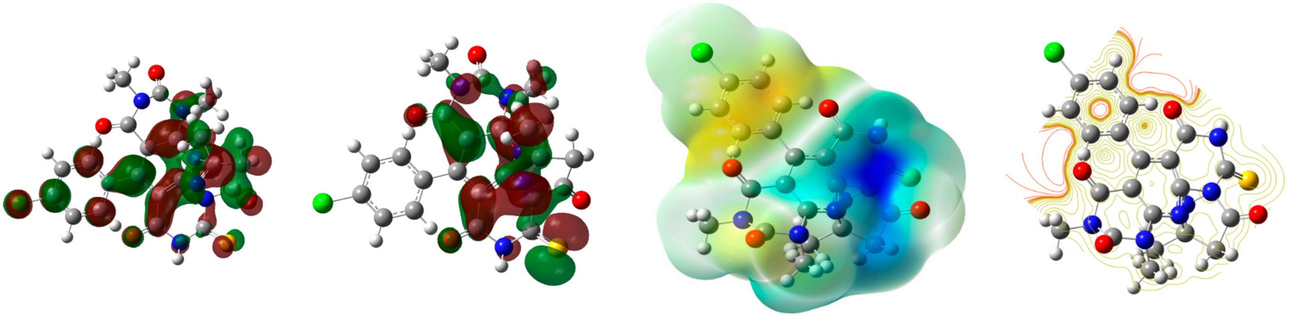

Table S10. ESP area distribution of compounds **1–16**. Red/Orange/Yellow = Negative ESP regions; Blue/Cyan = Positive ESP regions; Green = Neutral regions. Values represent the percentage contribution of each surface area to the total molecular ESP map.

Compound N.	**Negative Area %**	**Positive Area %**	**Neutral Area %**
1	11.68	20.25	25.29
2	14.82	9.85	32.53
3	12.19	11.22	32.63
4	8.16	13.70	41.62
5	8.80	26.86	27.42
6	10.81	8.79	34.06
7	15.14	6.72	38.20
8	15.65	6.85	40.29
9	15.37	8.94	41.47
10	12.91	7.13	40.65
11	11.78	8.60	42.93
12	6.00	24.89	33.00
13	8.14	3.81	34.97
14	12.18	7.35	42.99
15	13.17	6.90	40.17
16	10.98	7.02	45.34

Table S11. Electrostatic Potential Surface Maps of Synthesized Compounds.

1

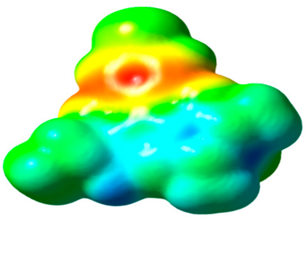

2

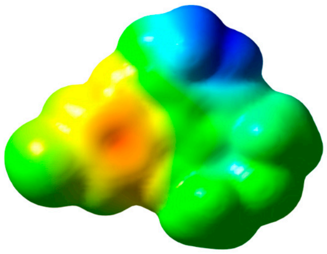

3

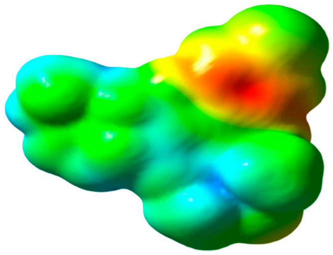

4

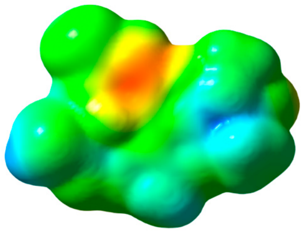

5

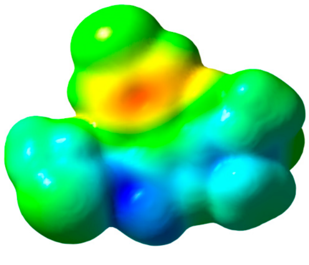

6

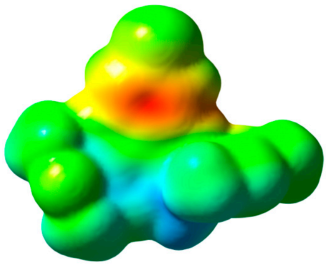

7

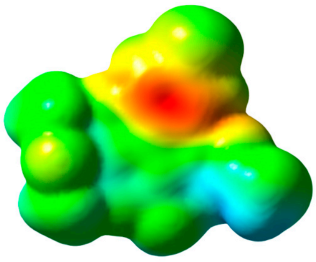

8

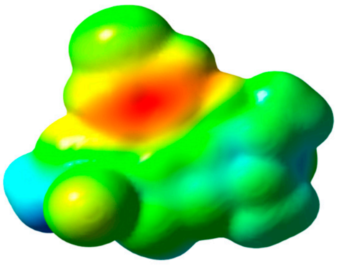

9

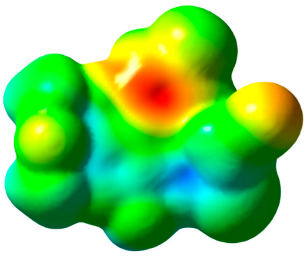

10

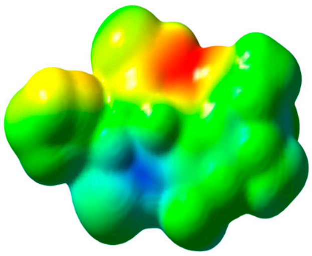

11

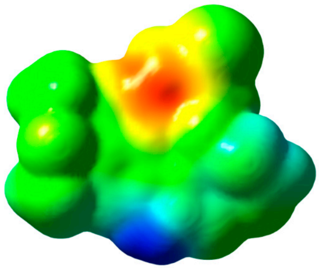

12

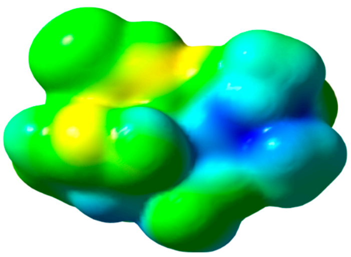

13

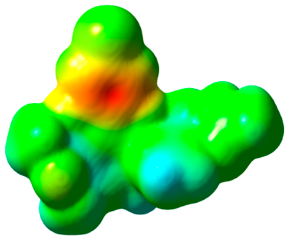

14

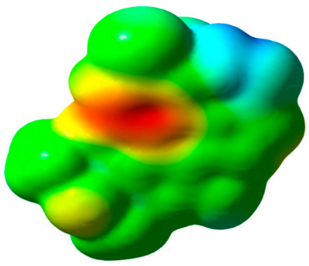

15

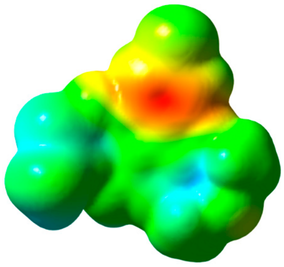

16

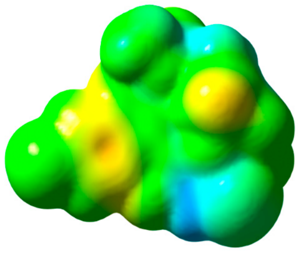

Copies of IR, Mass, ^{1}H NMR, and ^{13}C NMR spectra of Compounds

Characterization of Compound 1:

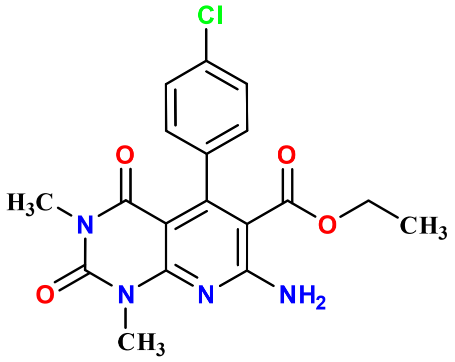

1

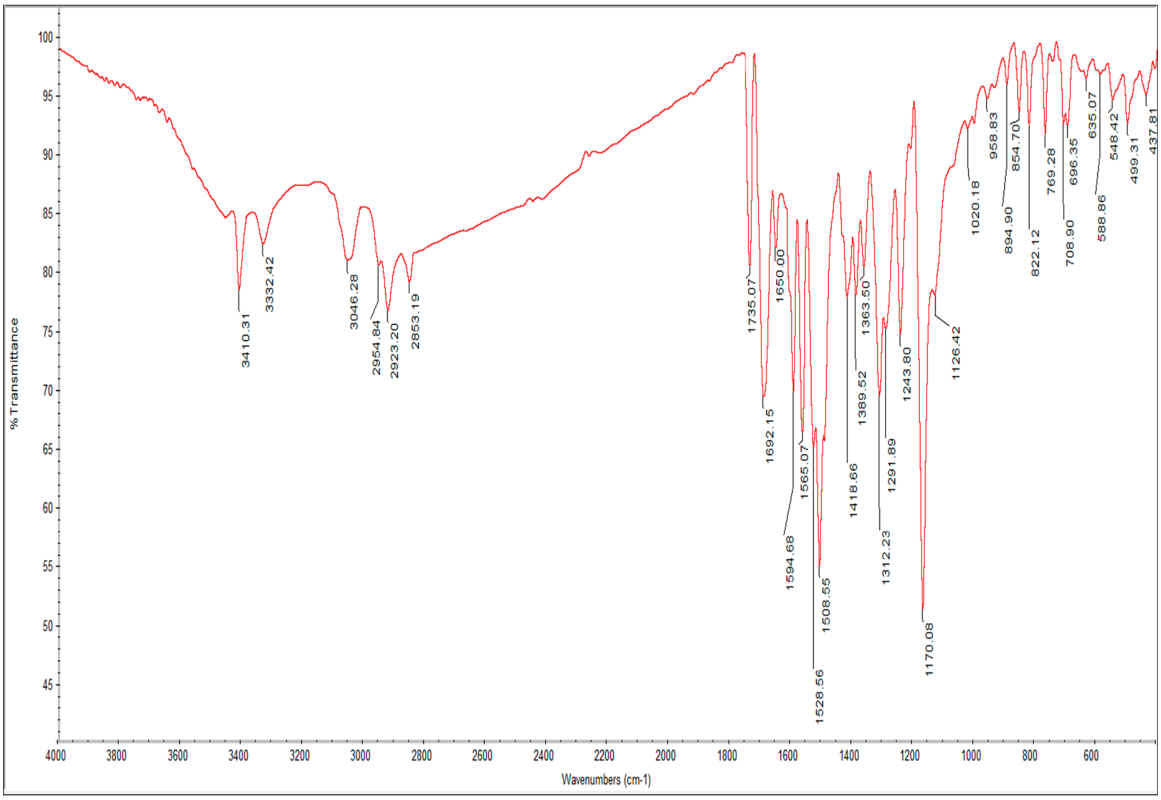

Figure S1 . IR of Compound **1**

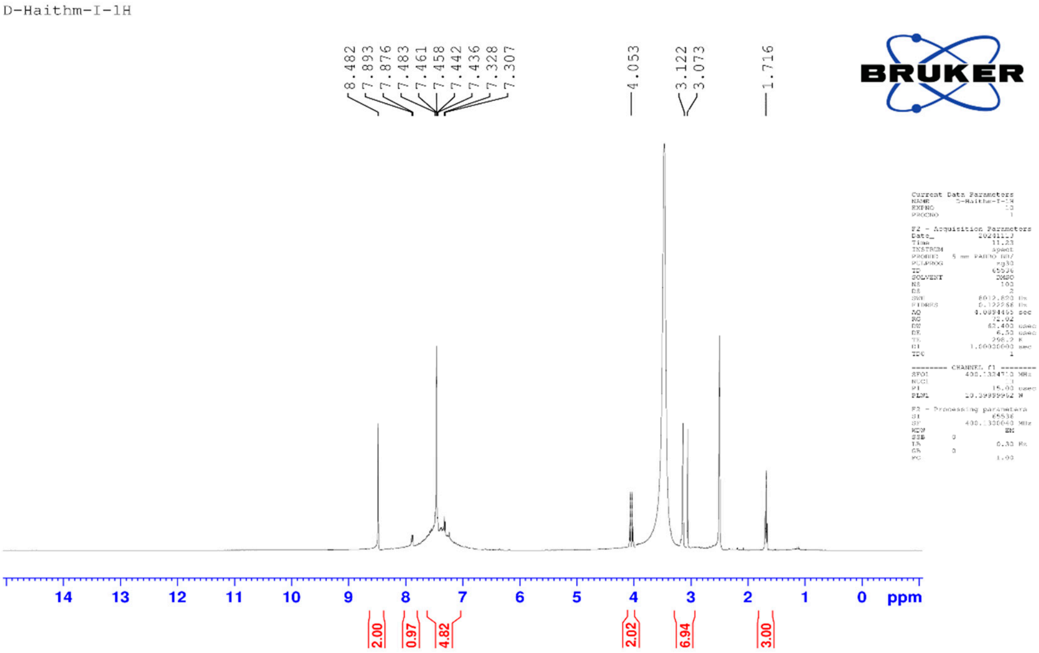

Figure S2. ^{1}H NMR spectrum (400 MHz, DMSO) of compound **1**

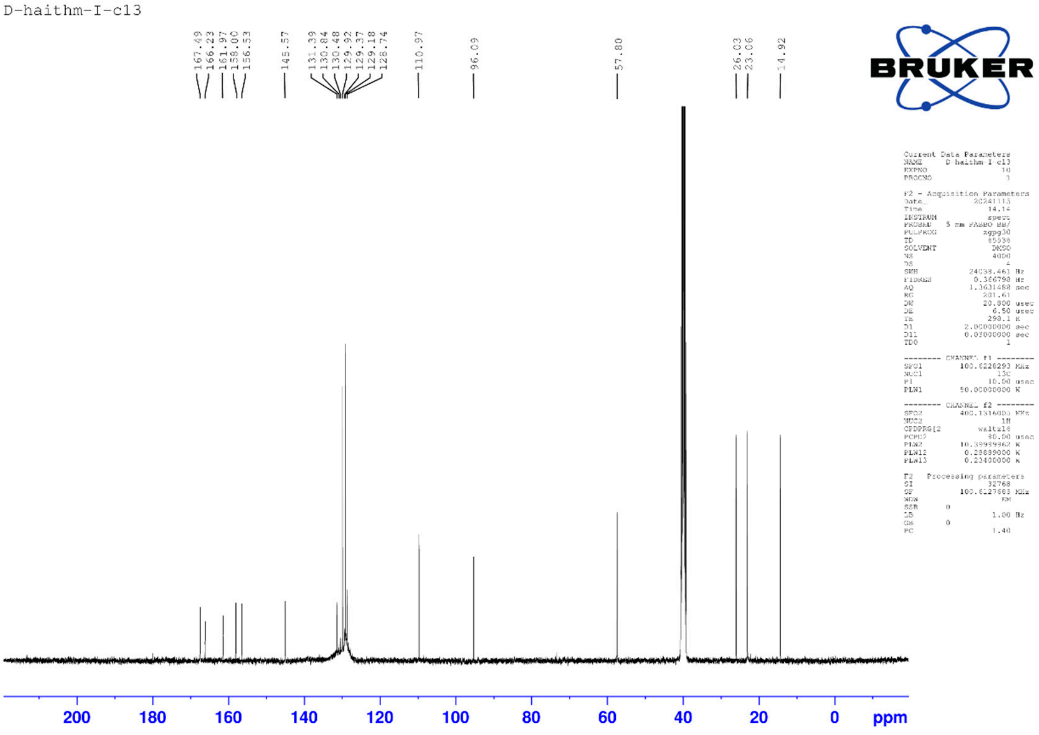

Figure S3. ^{13}C NMR spectrum (100 MHz, DMSO) of compound **1**

Characterization of Compound 2:-

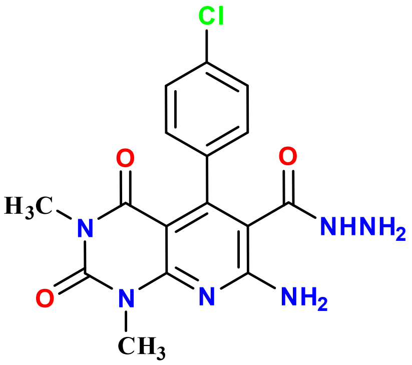

2

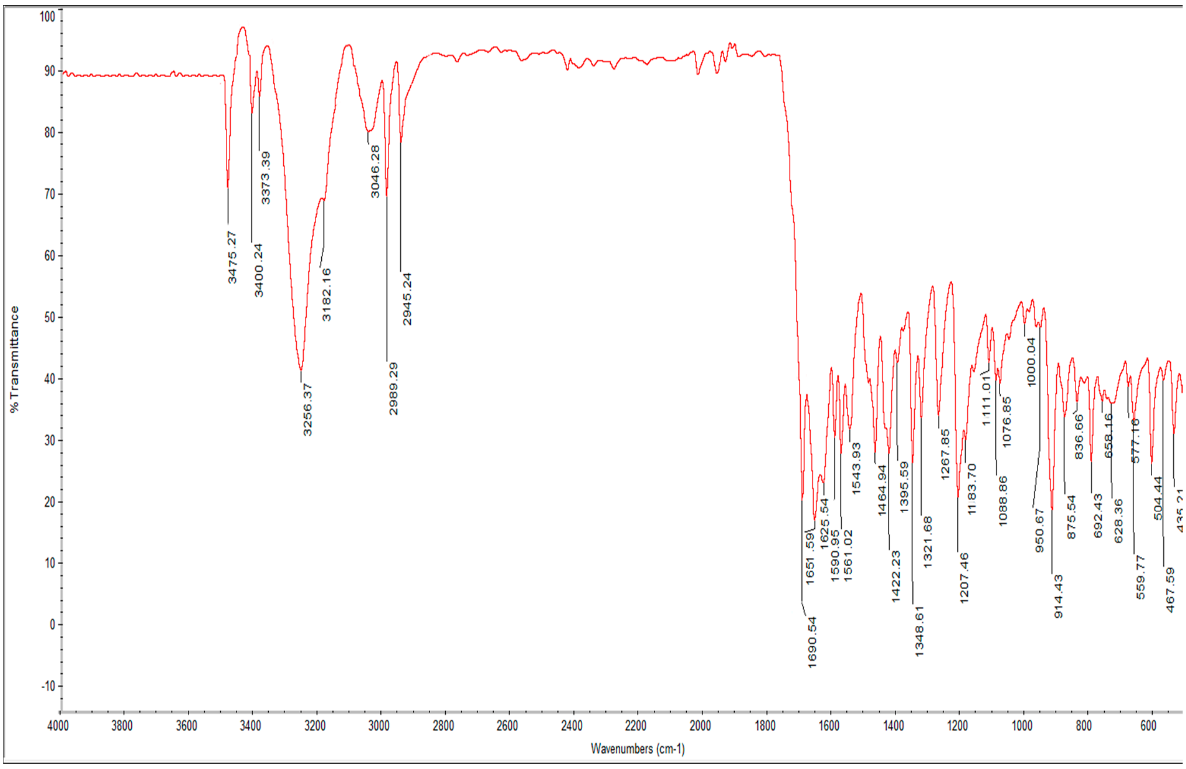

Figure S4 . IR of Compound **2**

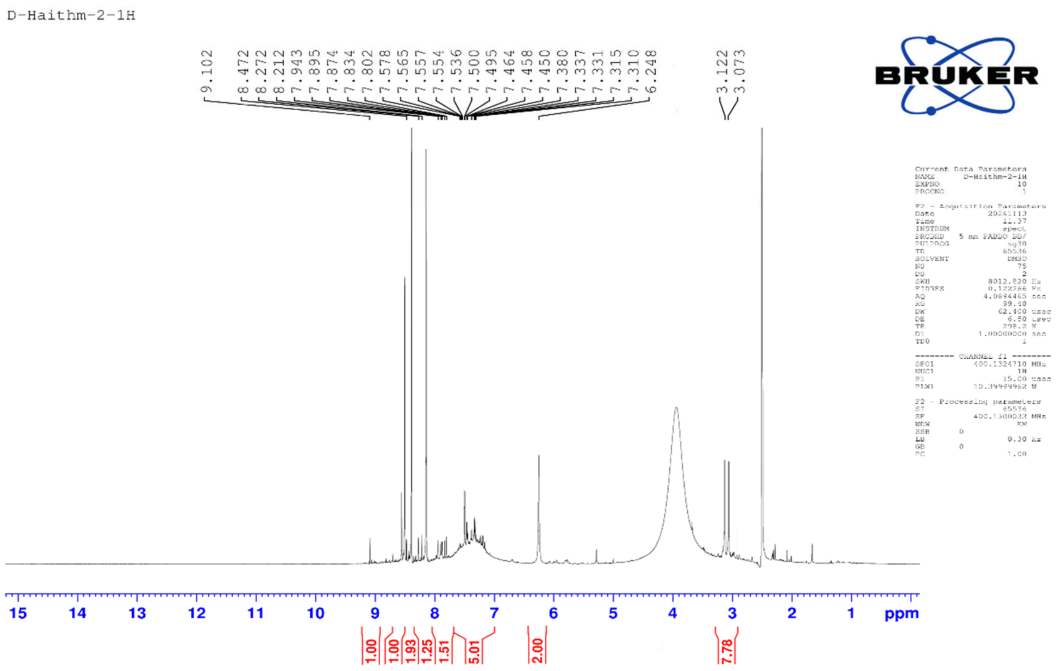

Figure S5. ^{1}H NMR spectrum (400 MHz, DMSO) of compound **2**

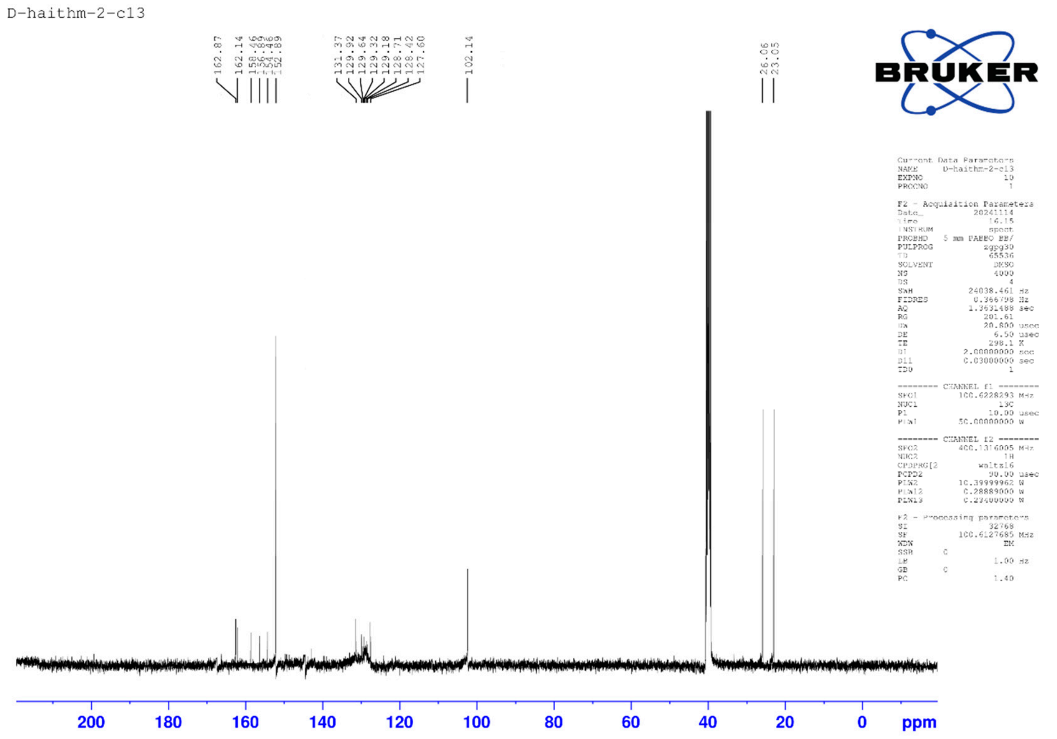

Figure S6. ^{13}C NMR spectrum (100 MHz, DMSO) of compound **2**

Characterization of Compound 3:-

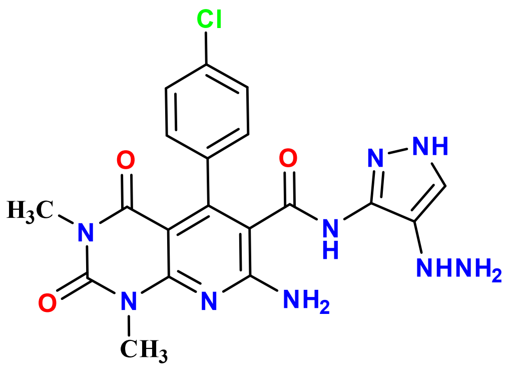

3

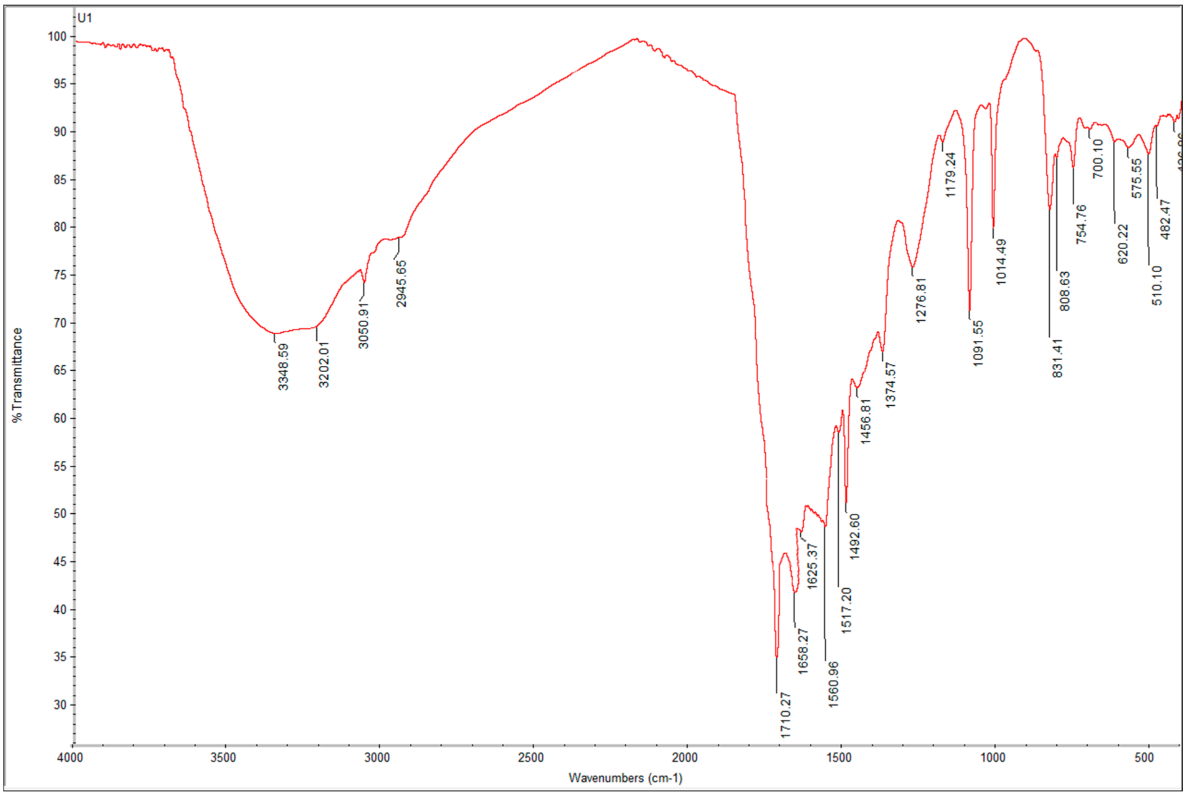

Figure S7 . IR of Compound **3**

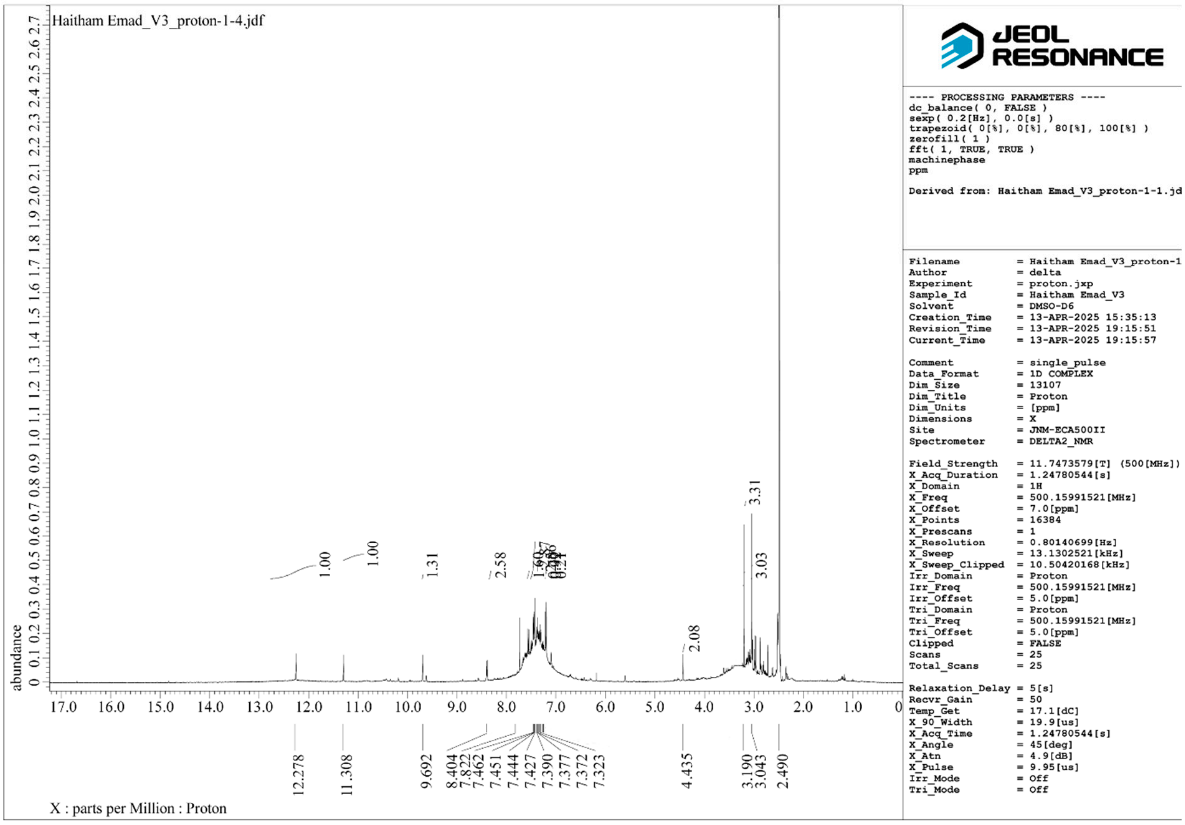

Figure S8. ^{1}H NMR spectrum (400 MHz, DMSO) of compound **3**

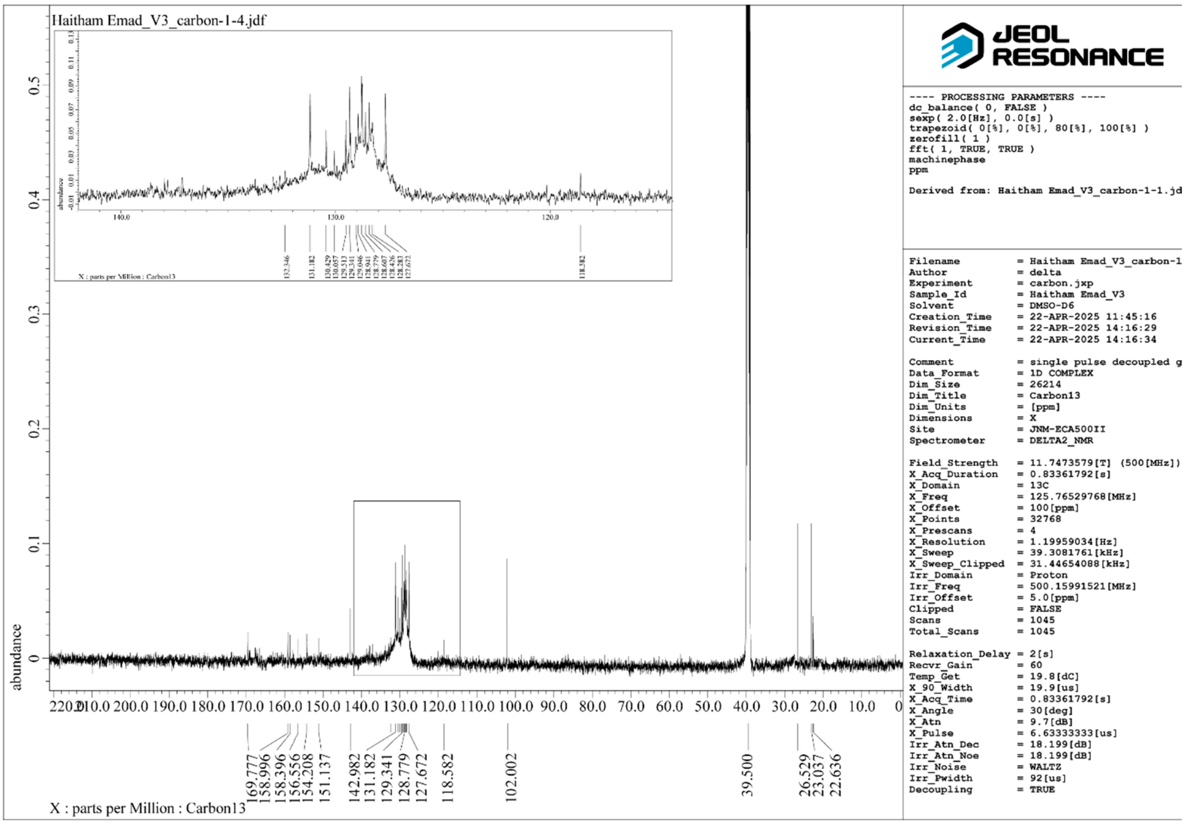

Figure S9. ^{13}C NMR spectrum (100 MHz, DMSO) of compound **3**

Characterization of Compound 4:-

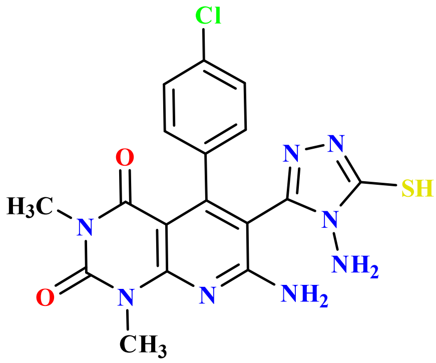

4

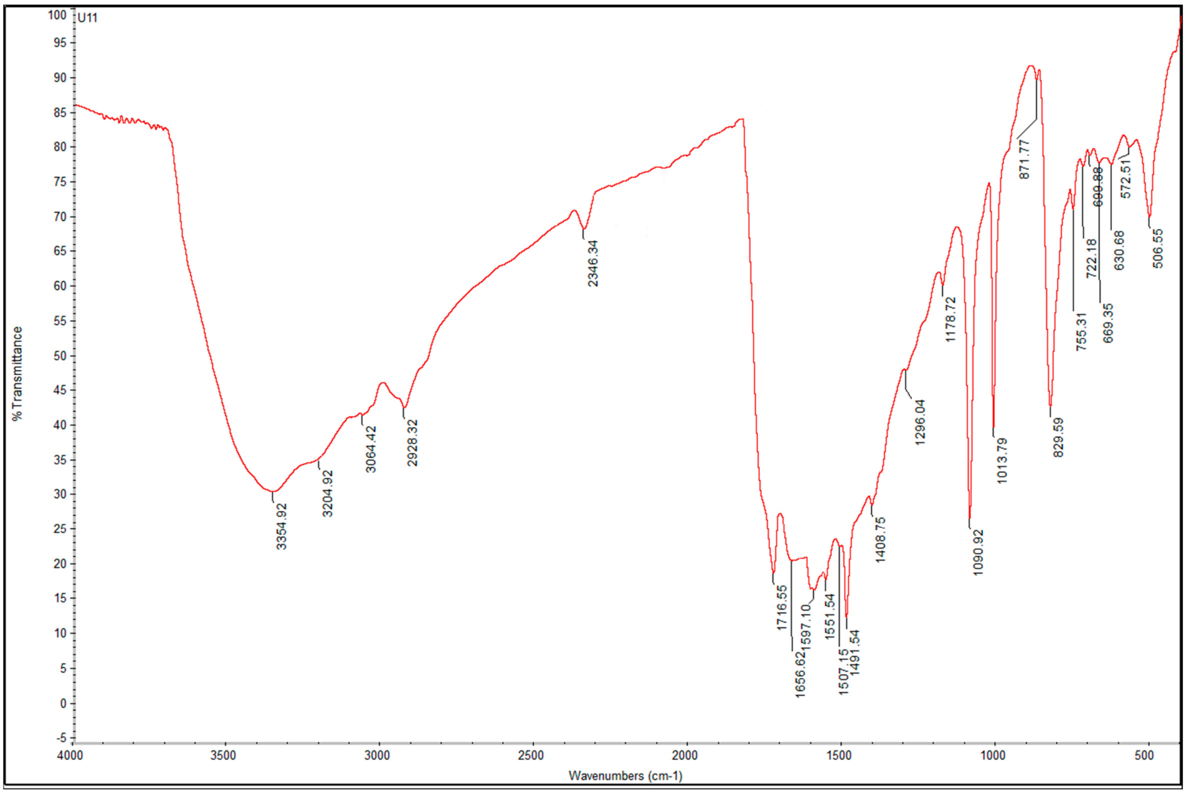

Figure S10 . IR of Compound **4**

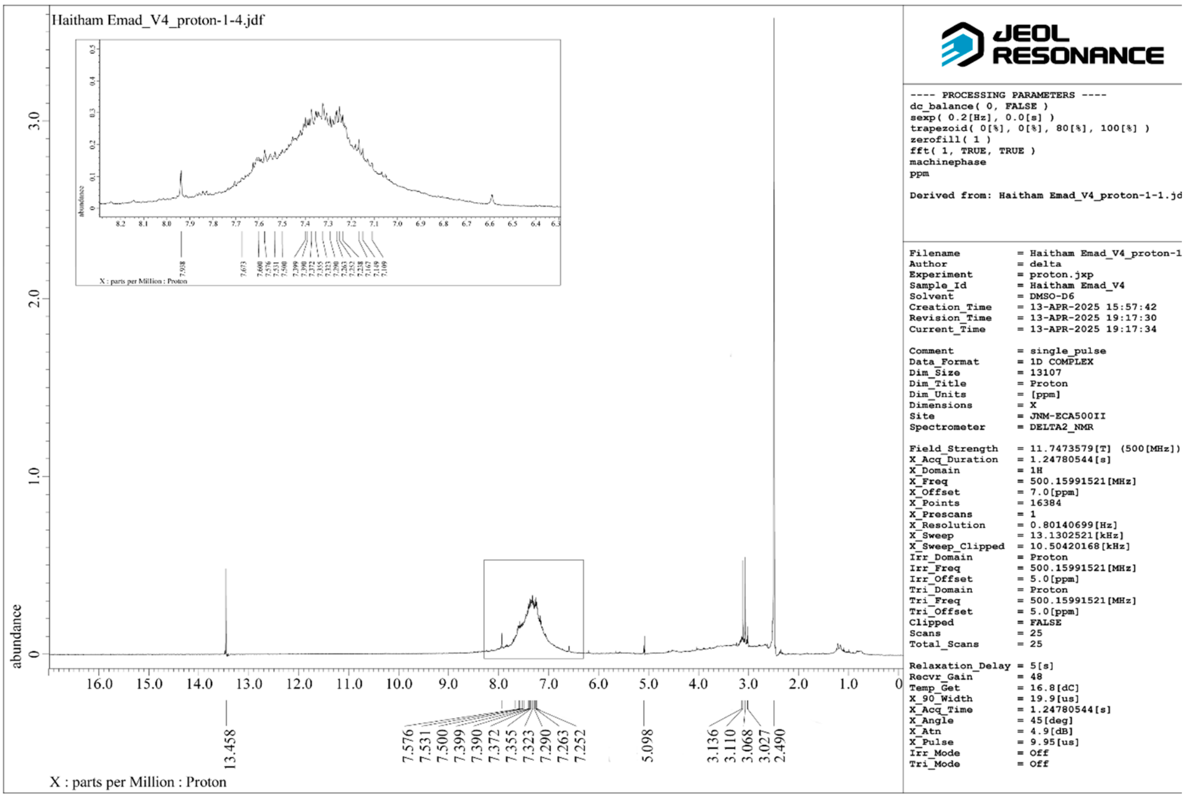

Figure S11. ^{1}H NMR spectrum (400 MHz, DMSO) of compound **4**

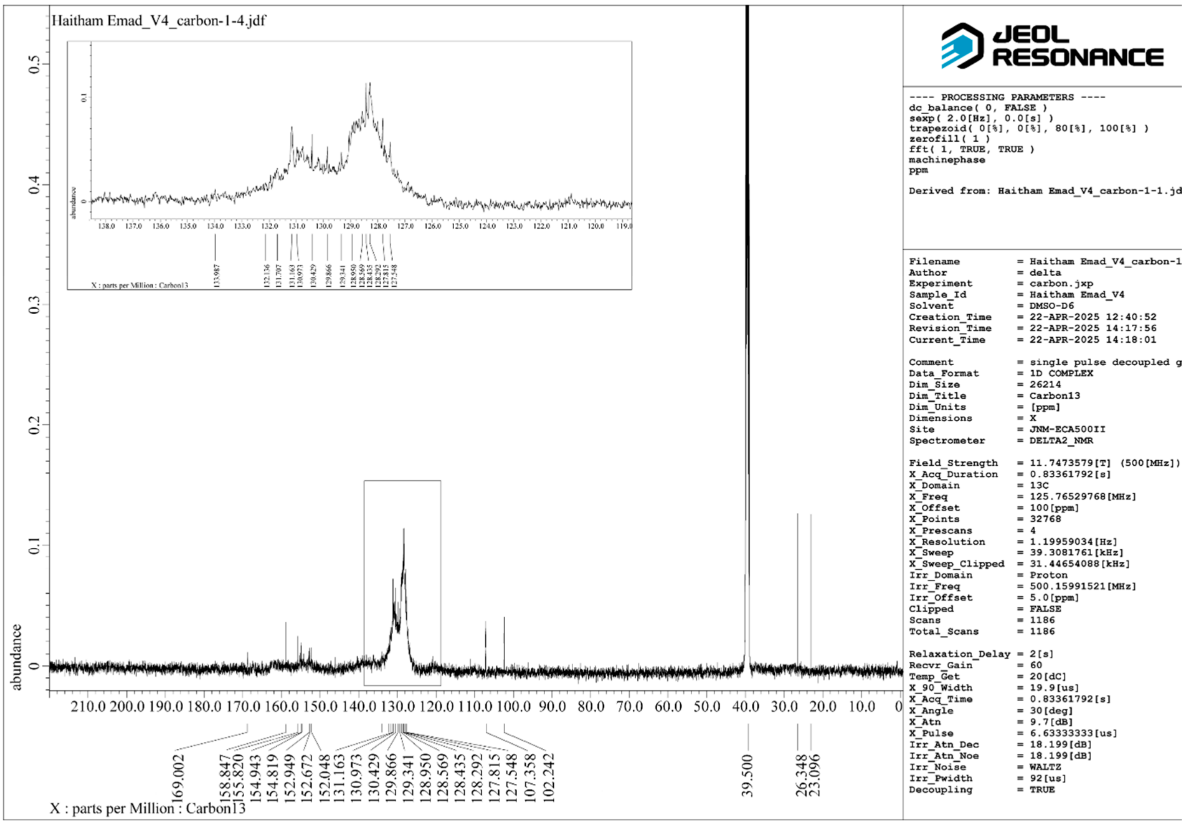

Figure S12. ^{13}C NMR spectrum (100 MHz, DMSO) of compound **4**

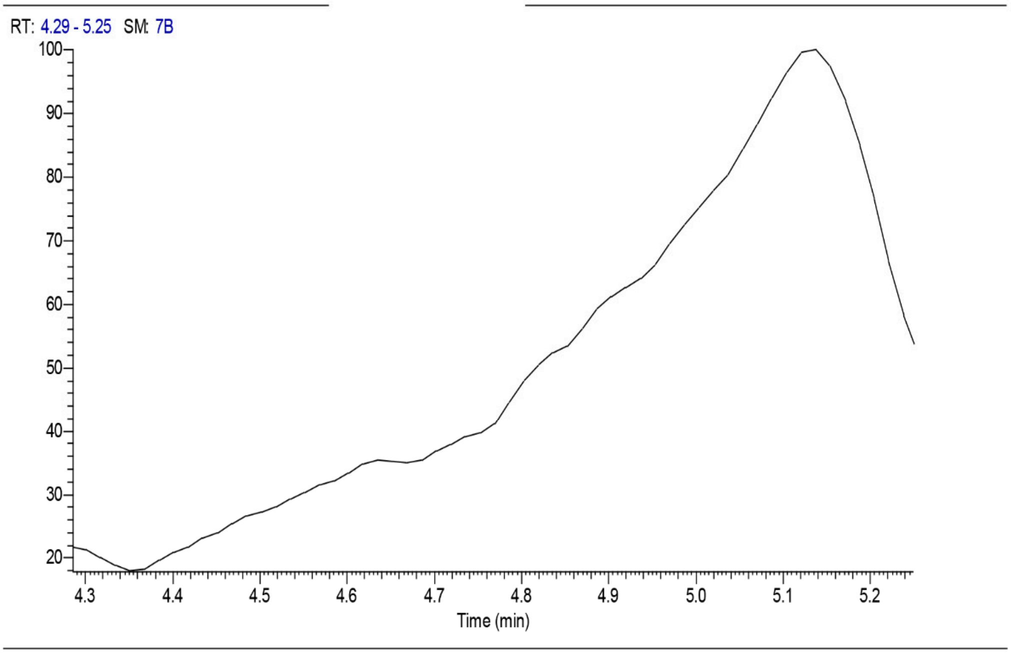

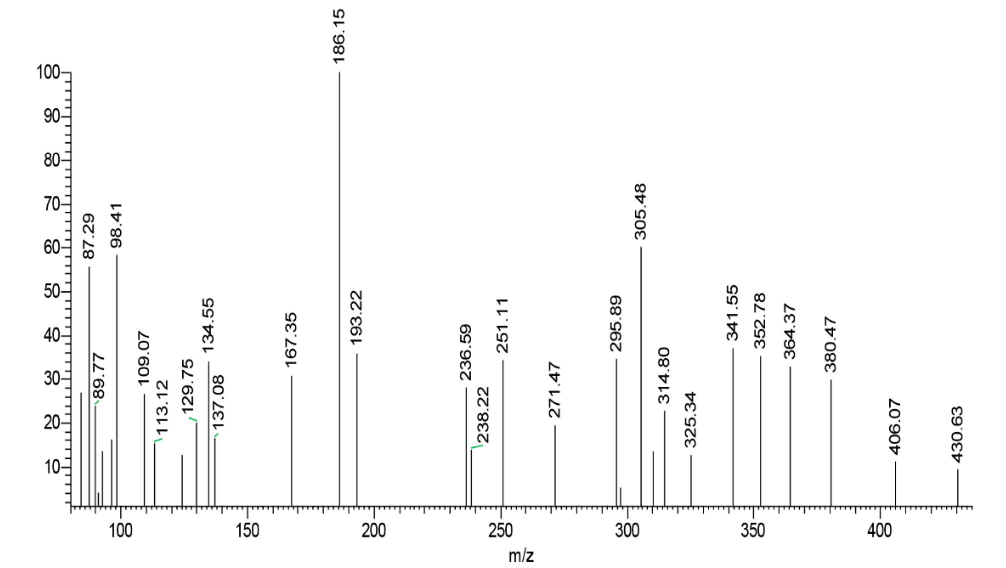

Figure S13. Mass spectrum of **4**

Characterization of Compound 5:-

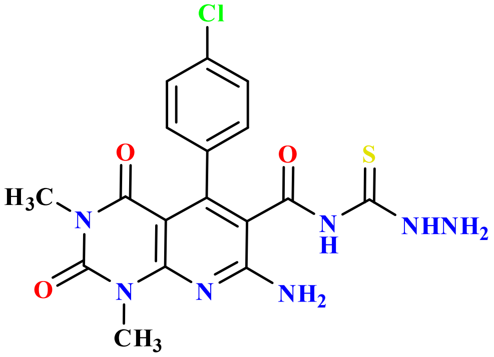

5

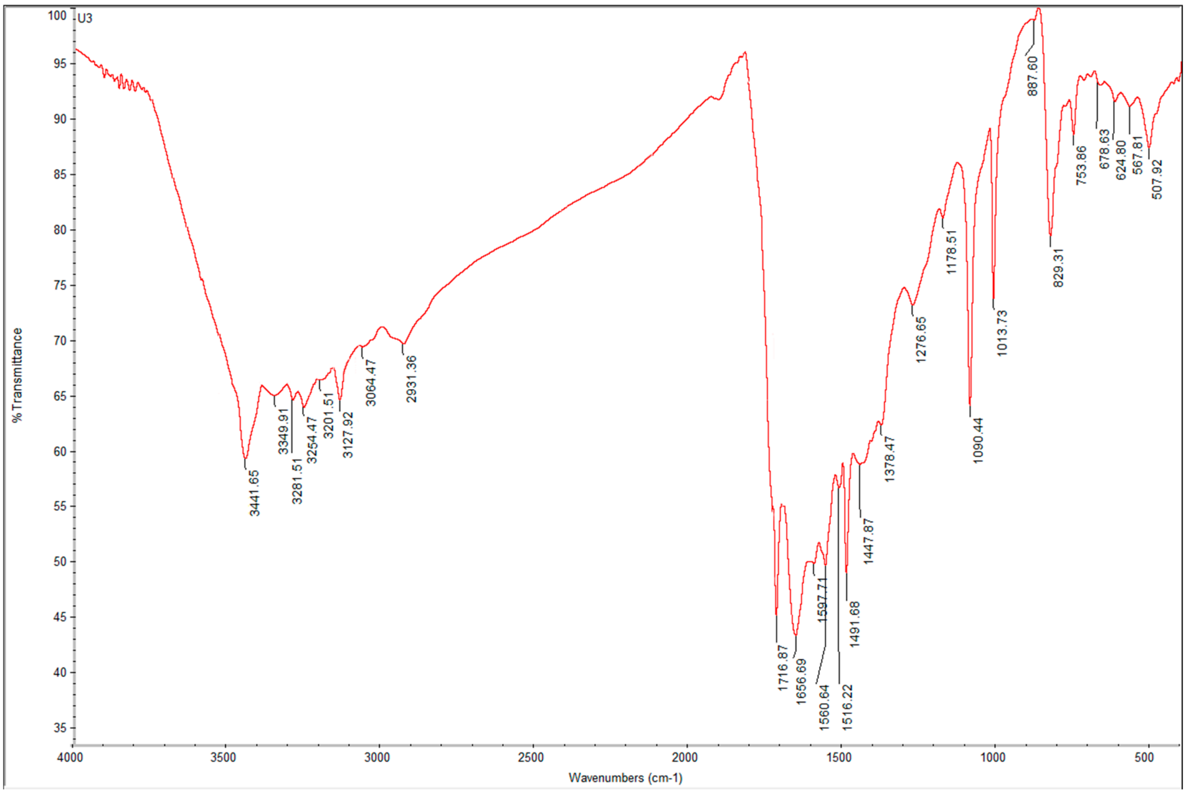

Figure S14 . IR of Compound **5**

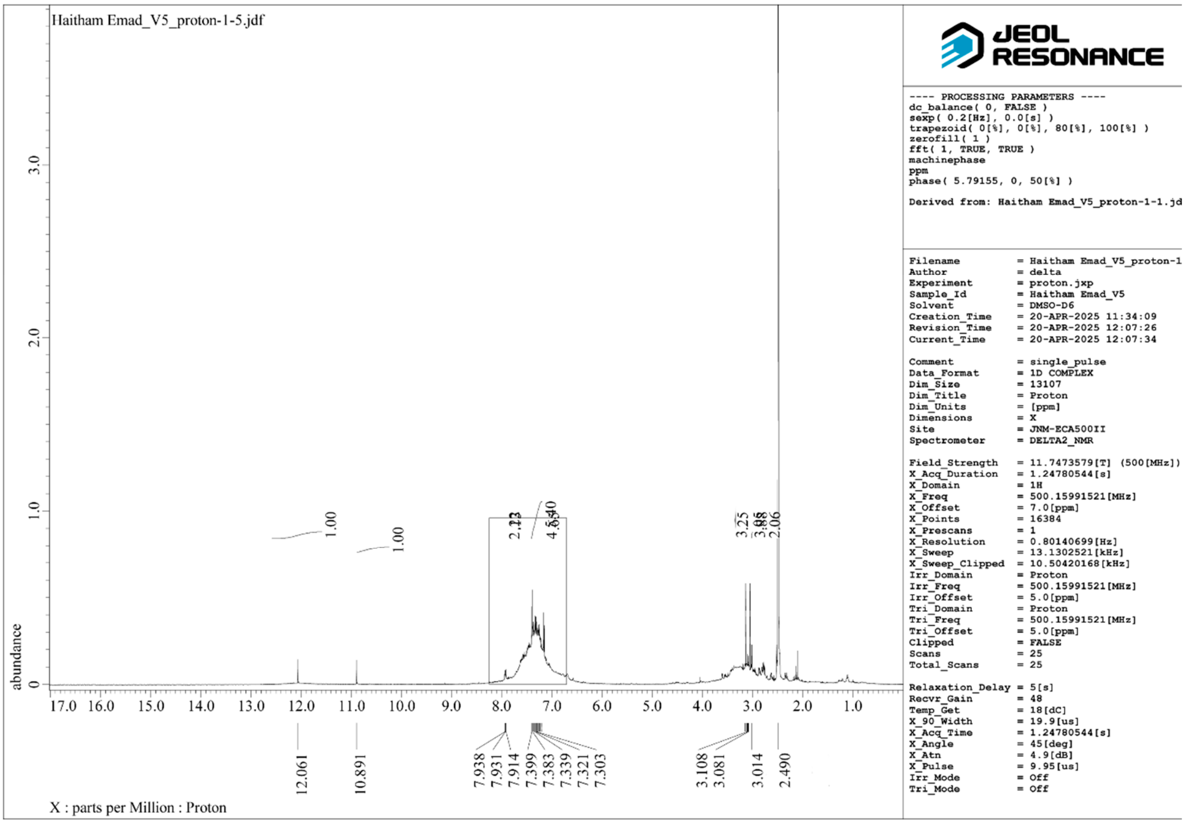

Figure S15. ^{1}H NMR spectrum (400 MHz, DMSO) of compound **5**

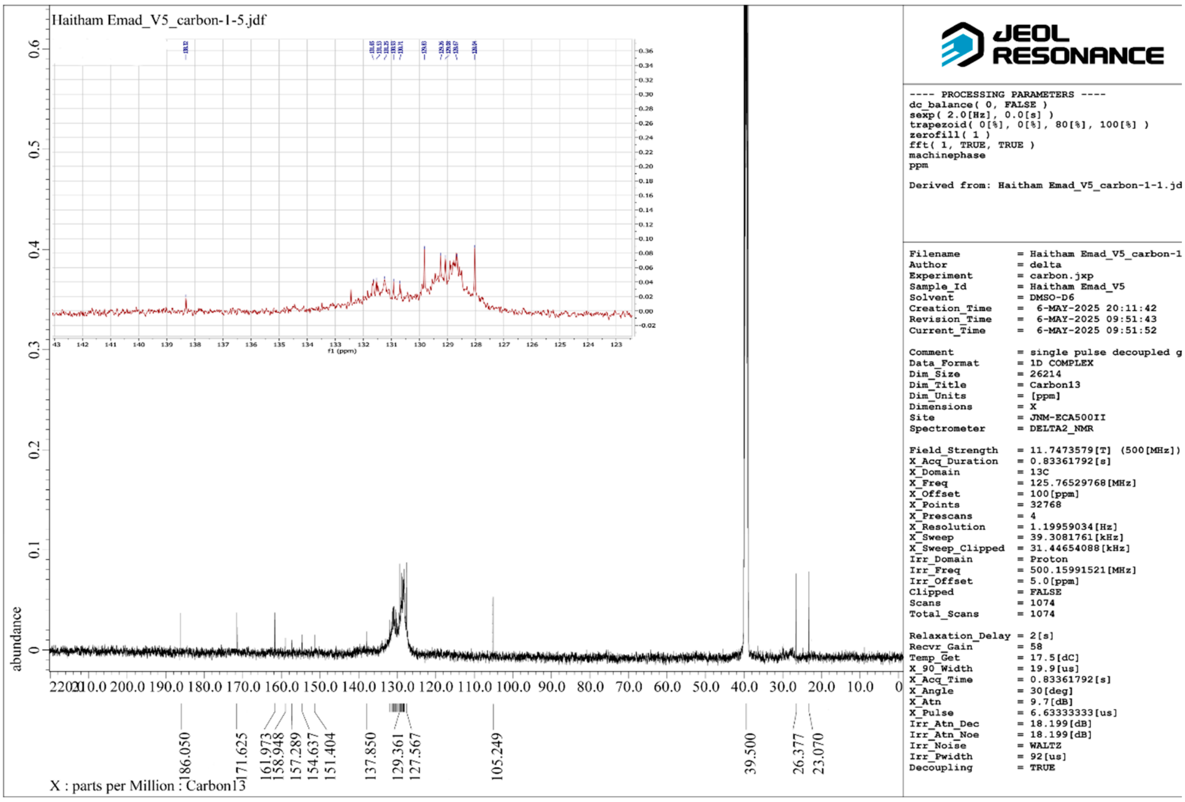

Figure S16. ^{13}C NMR spectrum (100 MHz, DMSO) of compound **5**

Characterization of Compound 6:-

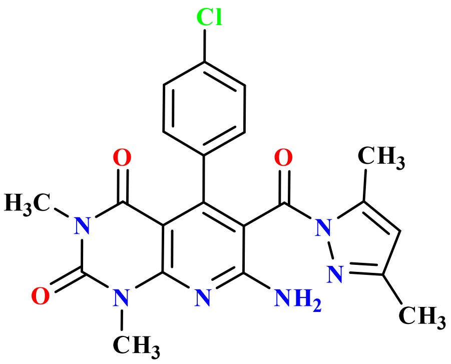

6

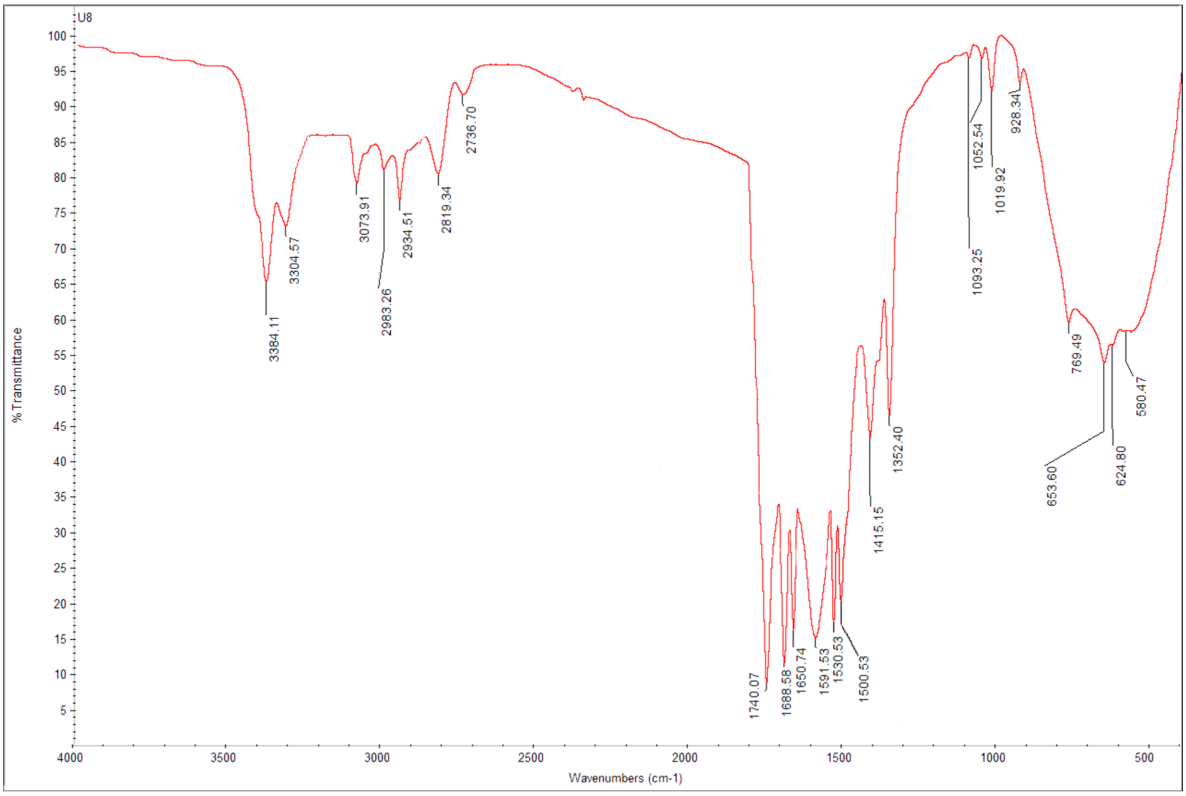

Figure S17. IR of Compound **6**

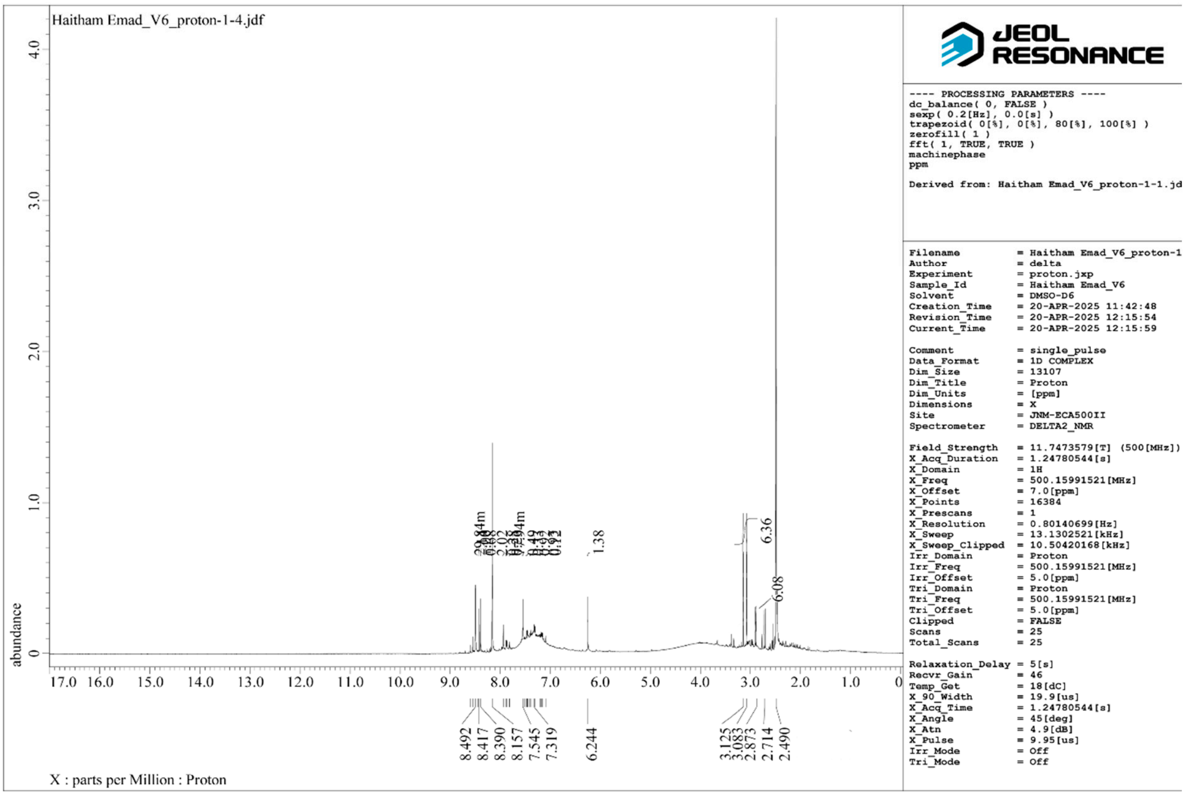

Figure S18. ^{1}H NMR spectrum (400 MHz, DMSO) of compound **6**

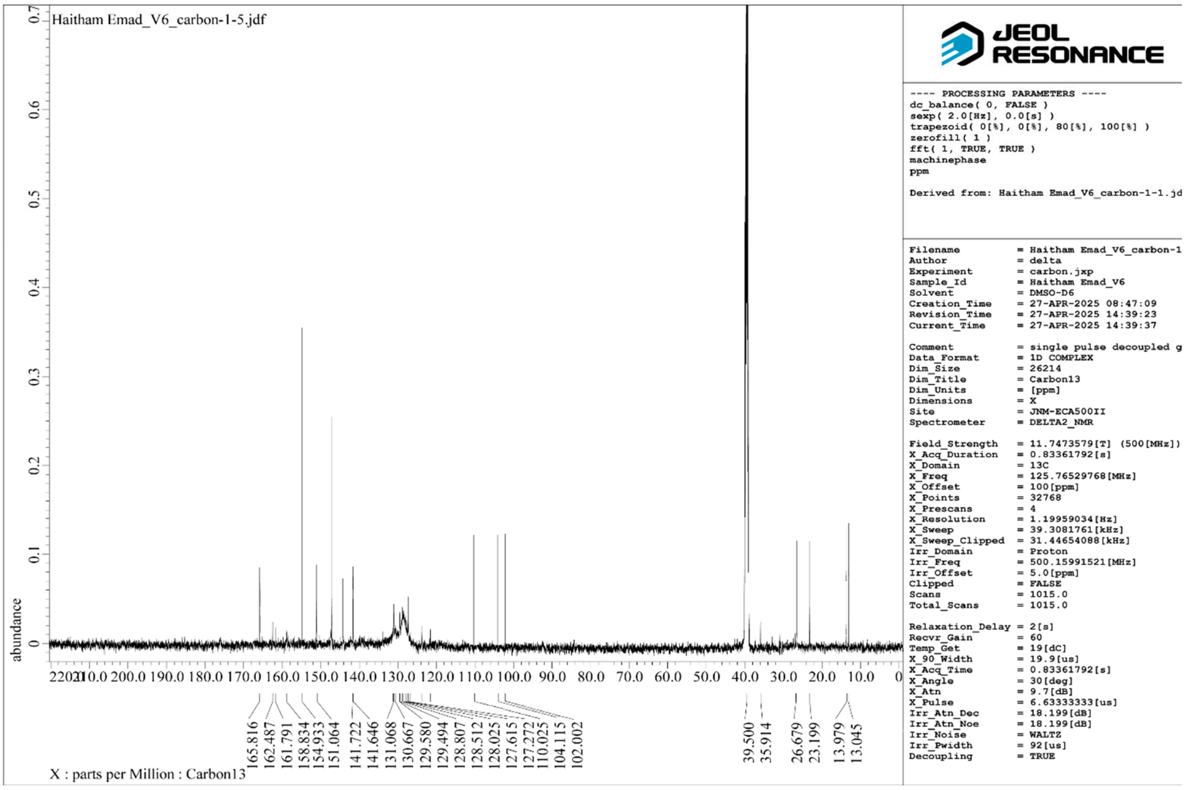

Figure S19. ^{13}C NMR spectrum (100 MHz, DMSO) of compound **6**

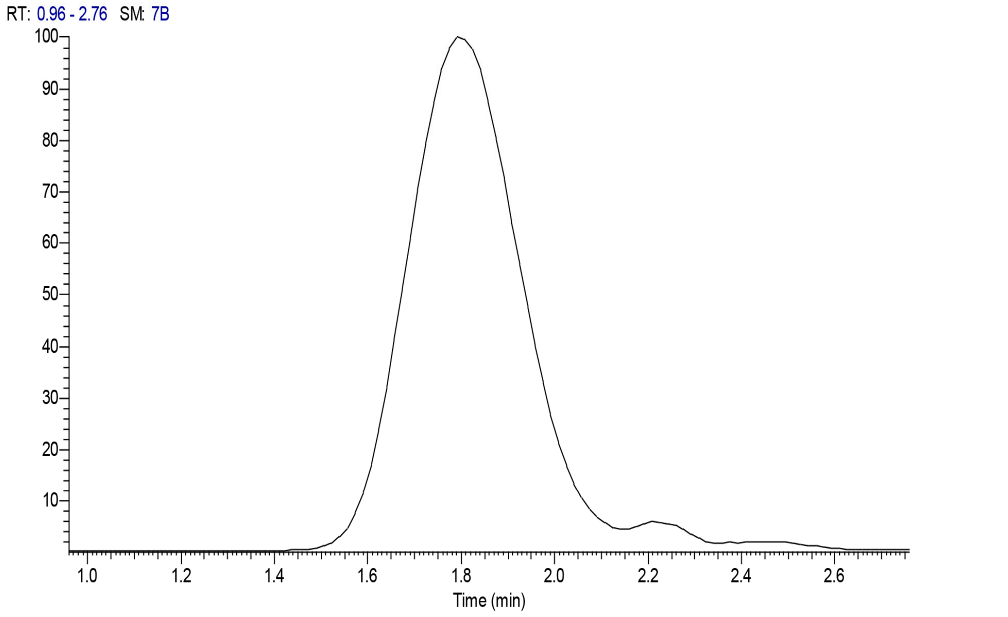

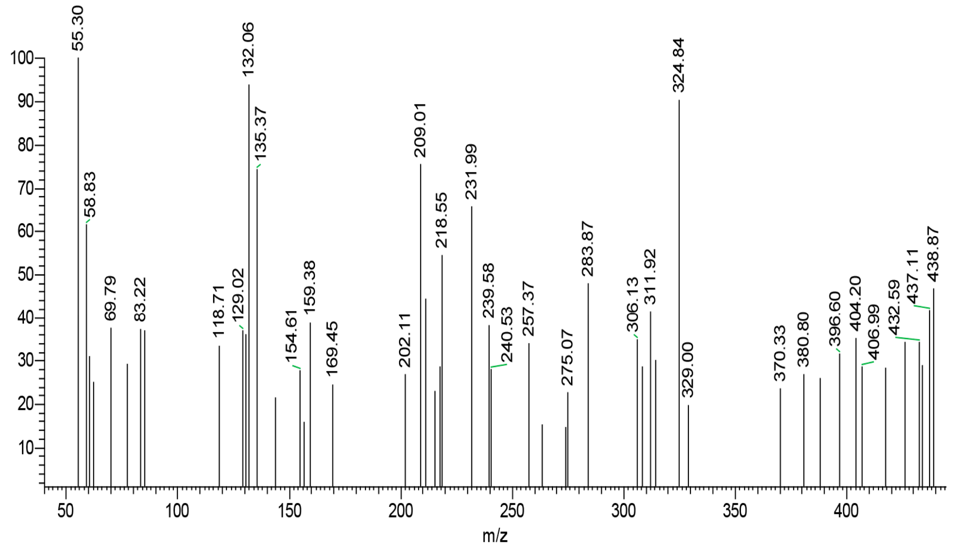

Figure S20. Mass spectrum of **6**

Characterization of Compound 7:-

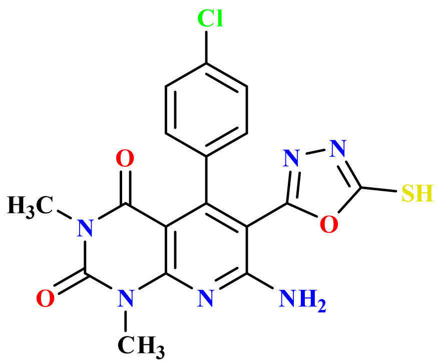

7

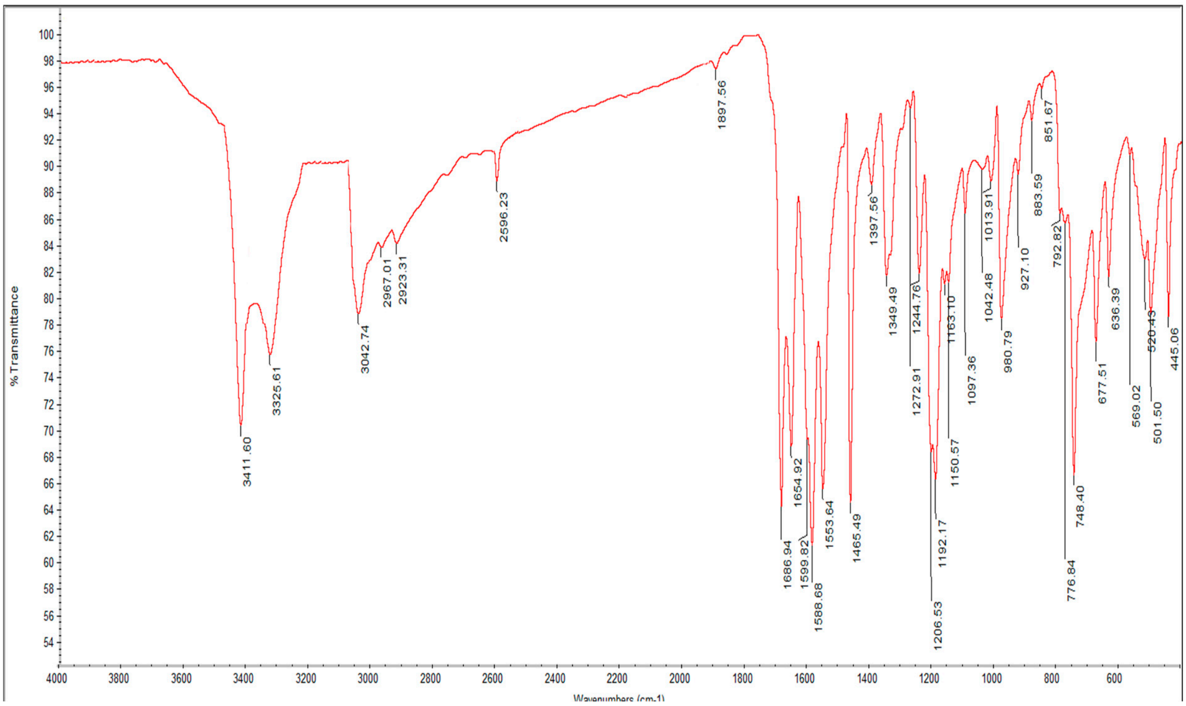

Figure S21 . IR of Compound **7**

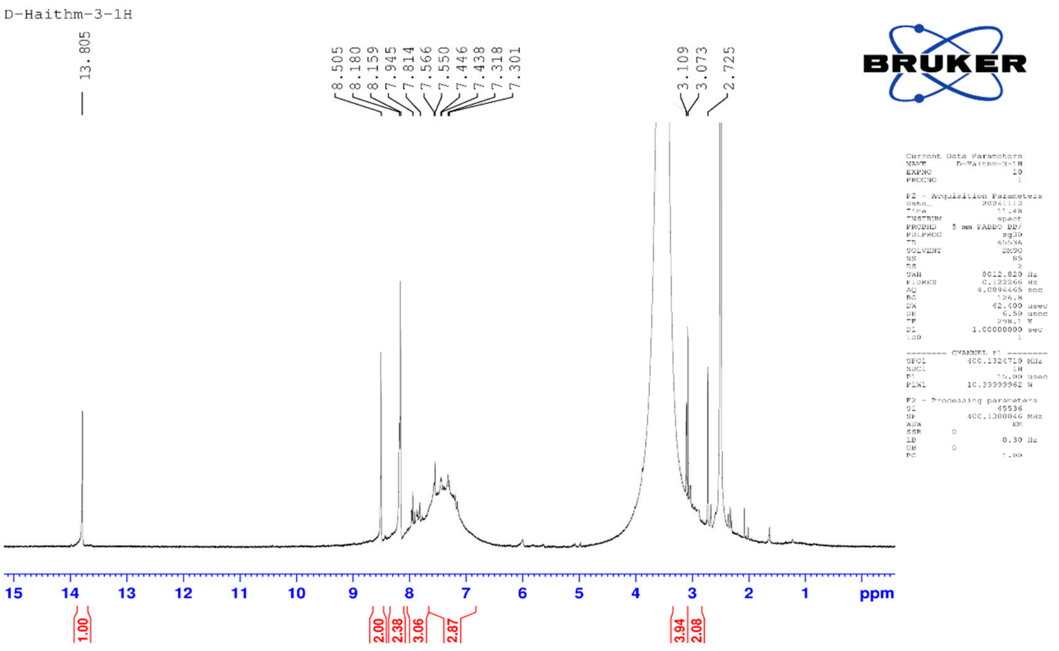

Figure S22. ^{1}H NMR spectrum (400 MHz, DMSO) of compound **7**

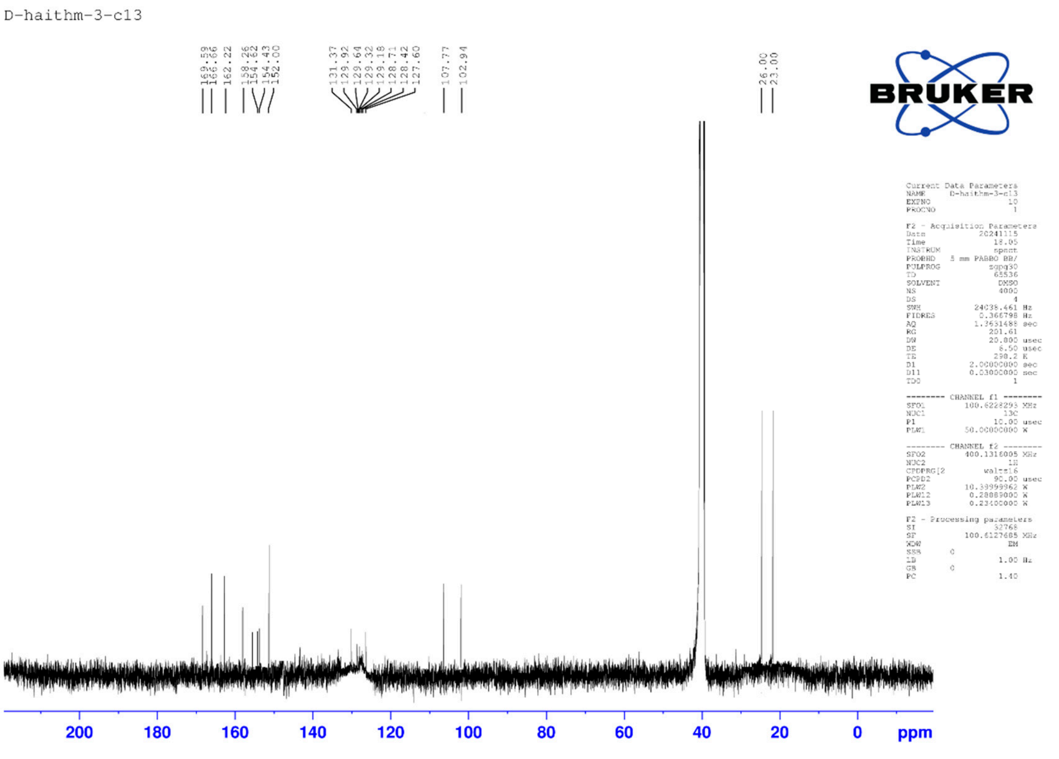

Figure S23. ^{13}C NMR spectrum (100 MHz, DMSO) of compound **7**

Characterization of Compound 8:-

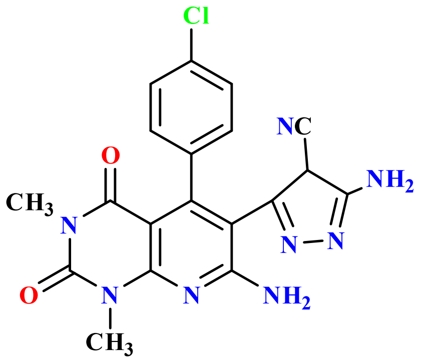

8

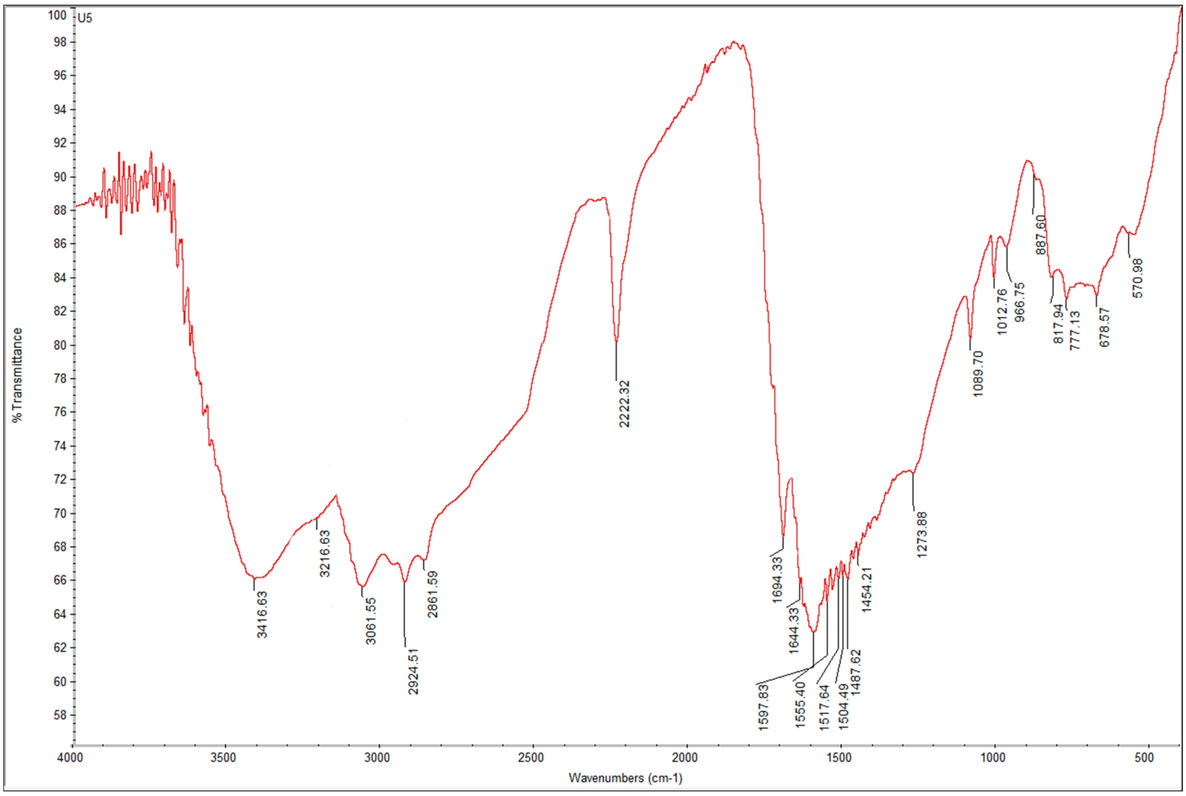

Figure S24 . IR of Compound **8**

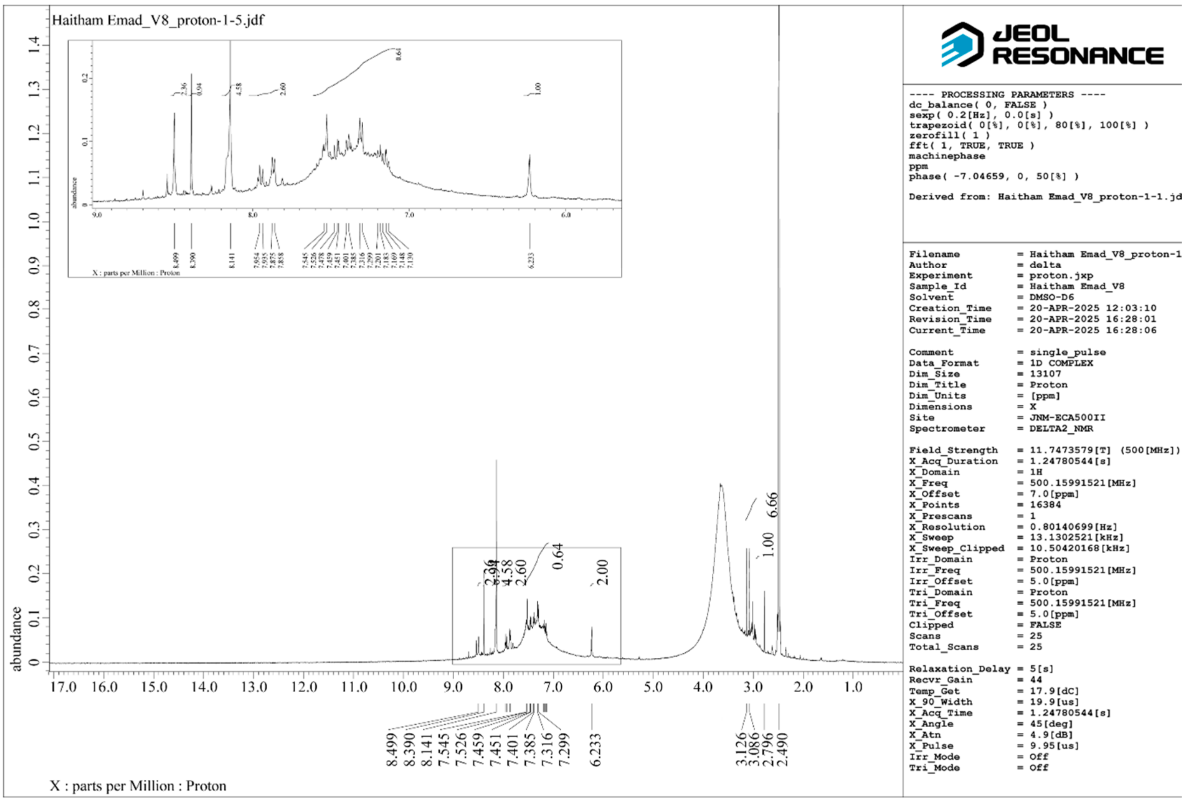

Figure S25. ^{1}H NMR spectrum (400 MHz, DMSO) of compound **8**

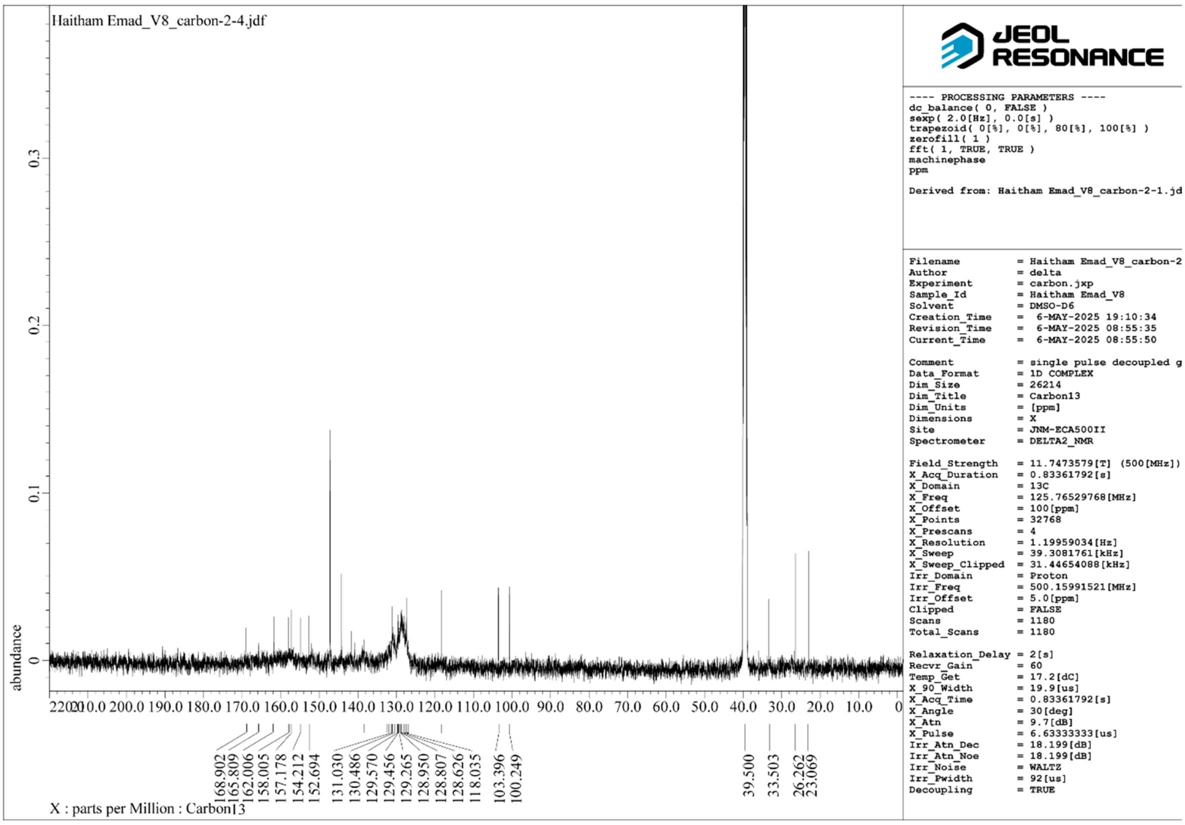

Figure S26. ^{13}C NMR spectrum (100 MHz, DMSO) of compound **8**

Characterization of Compound 9:-

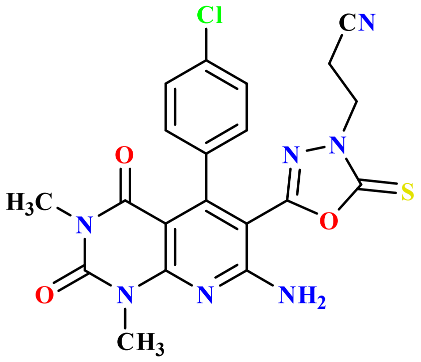

9

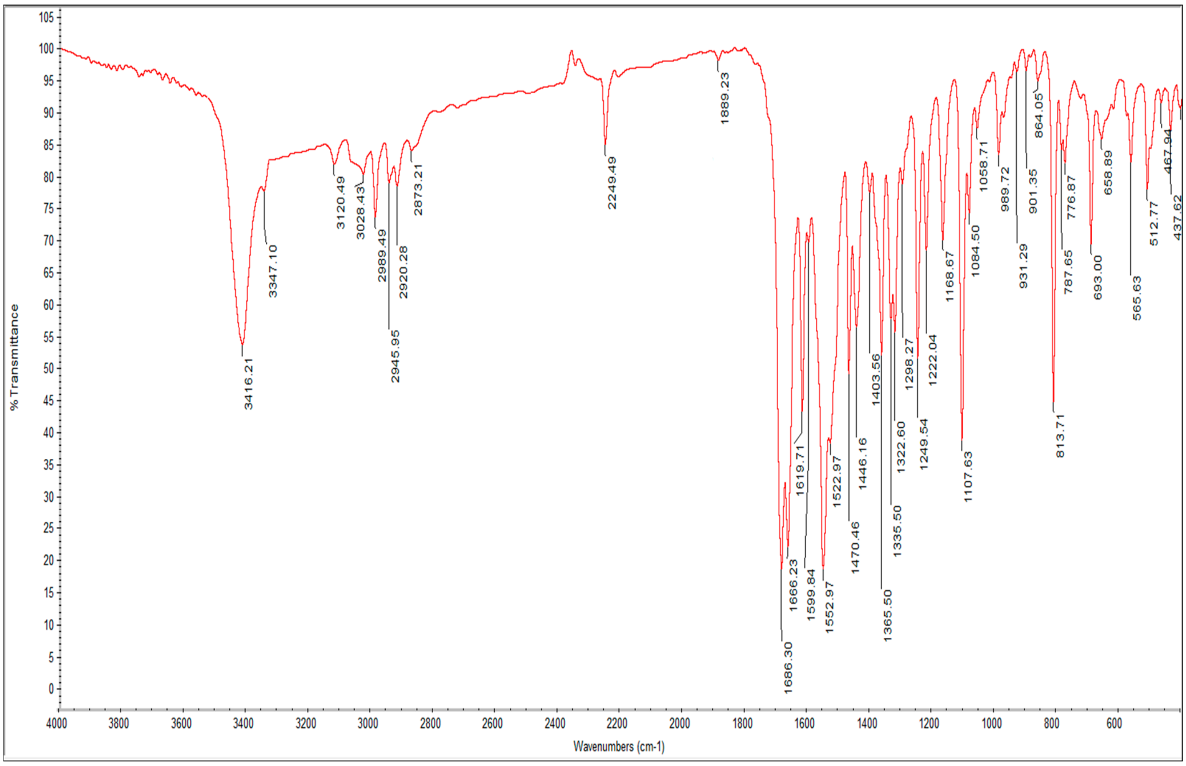

Figure S27 . IR of Compound **9**

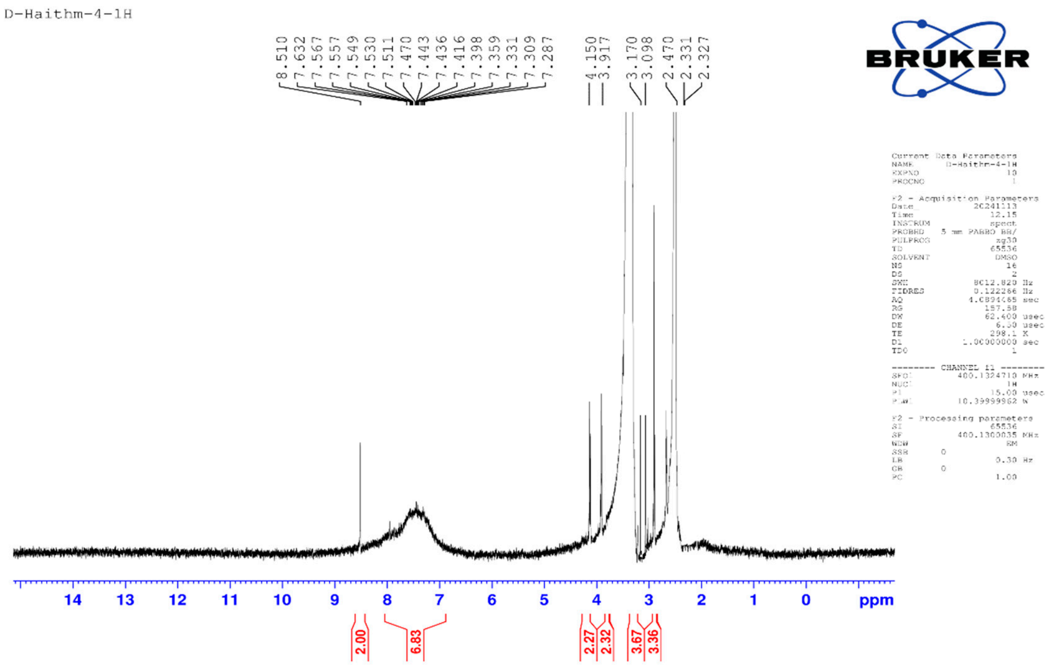

Figure S28. ^{1}H NMR spectrum (400 MHz, DMSO) of compound **9**

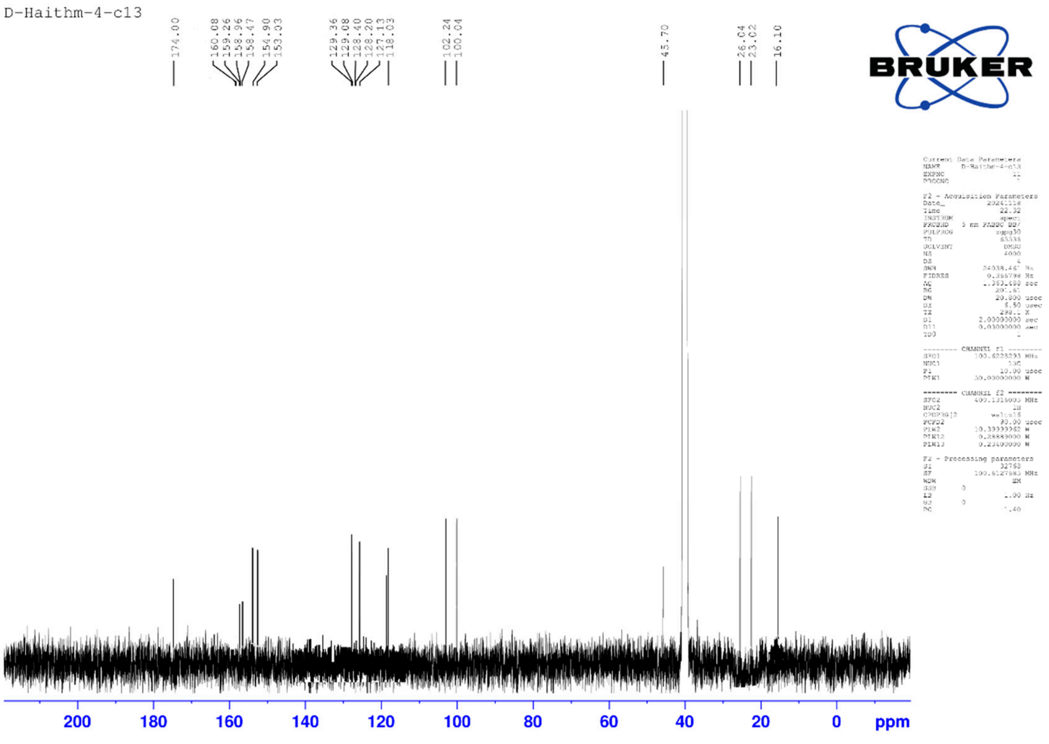

Figure S29. ^{13}C NMR spectrum (100 MHz, DMSO) of compound **9**

Characterization of Compound 10:-

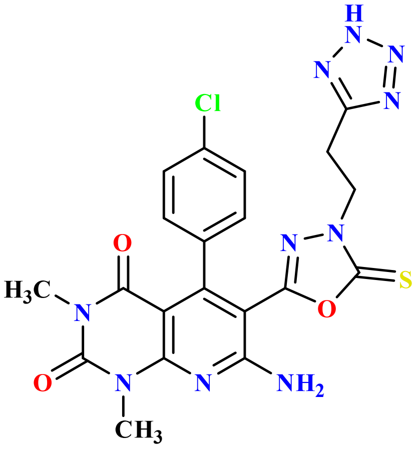

10

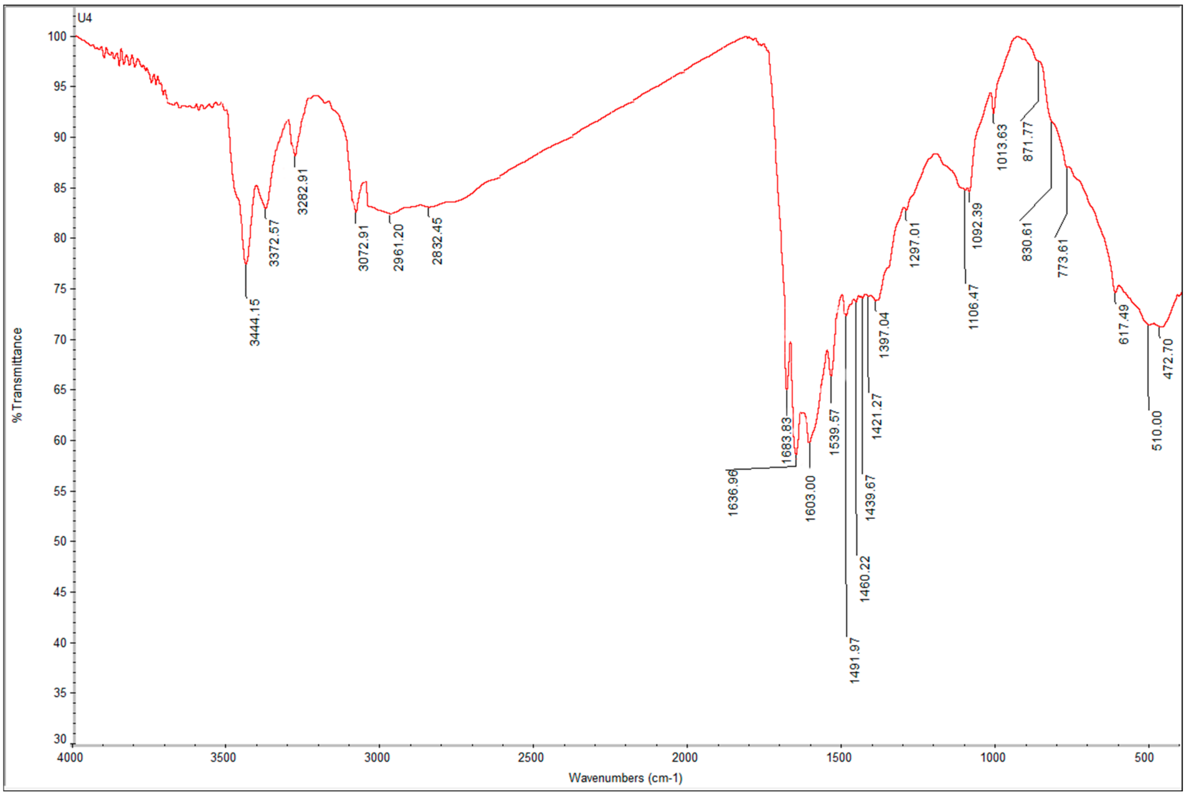

Figure S30 . IR of Compound **10**

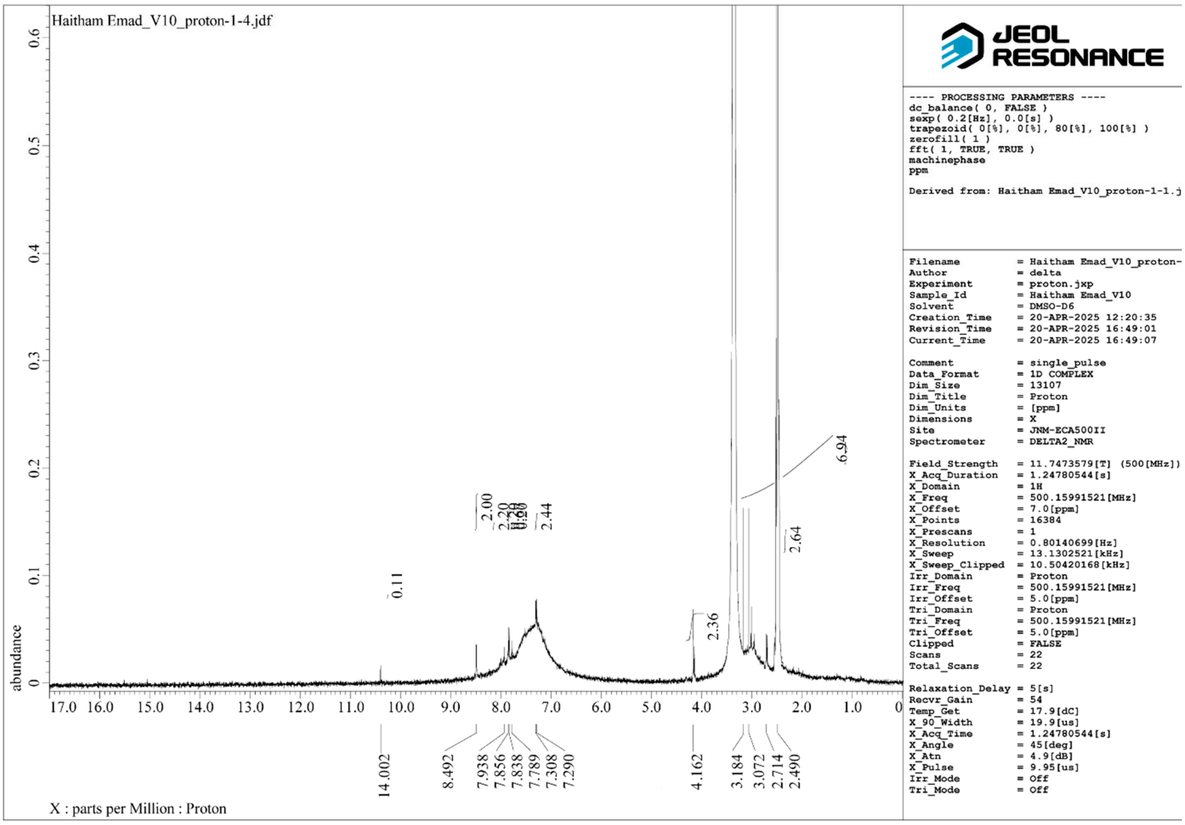

Figure S31. ^{1}H NMR spectrum (400 MHz, DMSO) of compound **10**

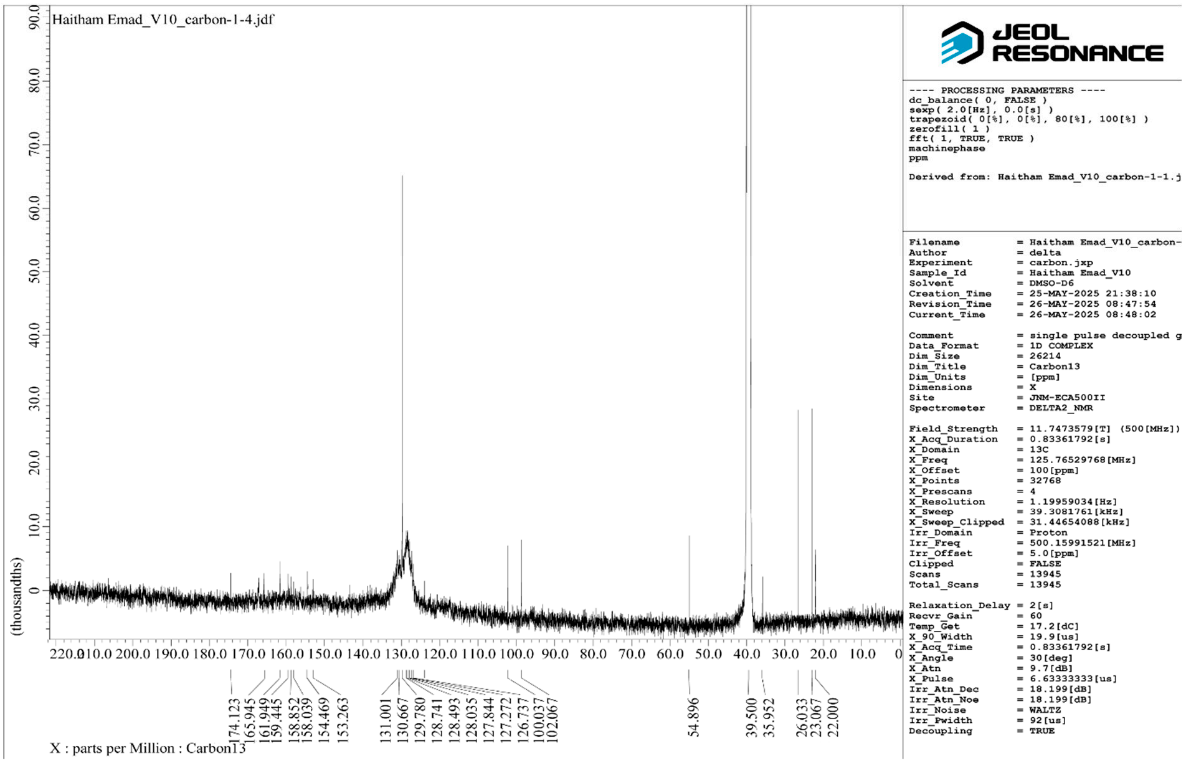

Figure S32. ^{13}C NMR spectrum (100 MHz, DMSO) of compound **10**

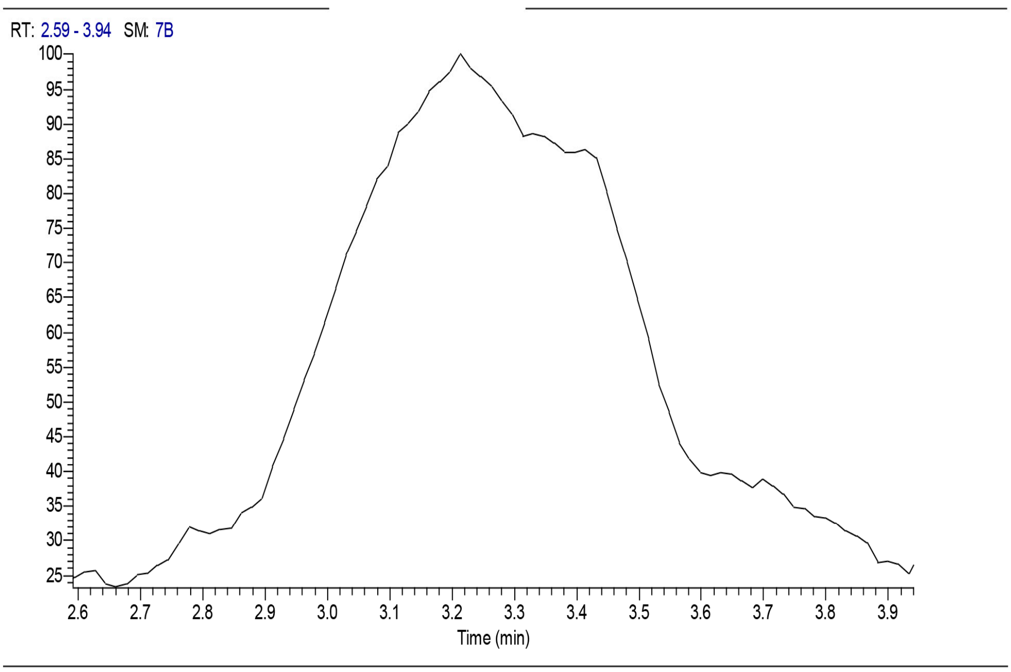

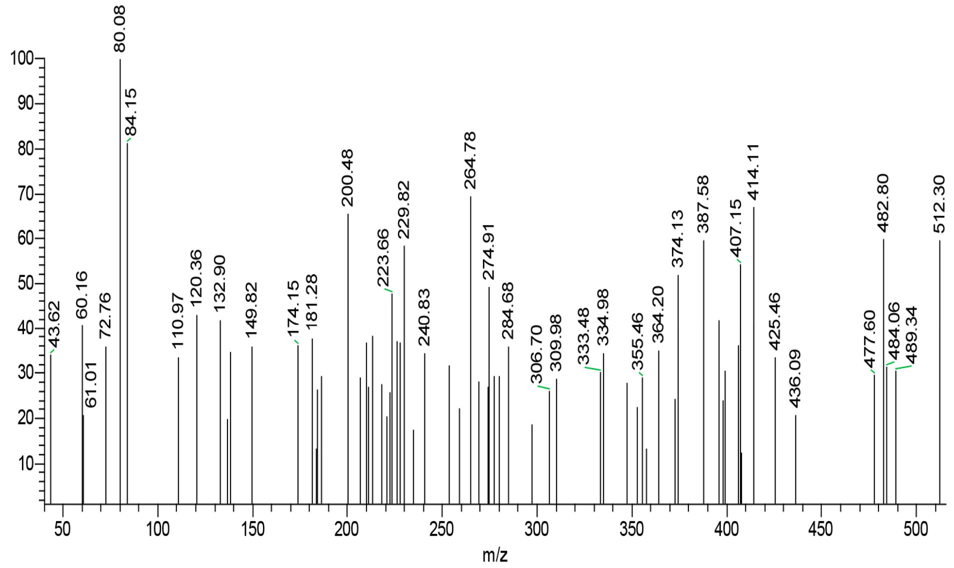

Figure S33. Mass spectrum of **10**

Characterization of Compound 11:-

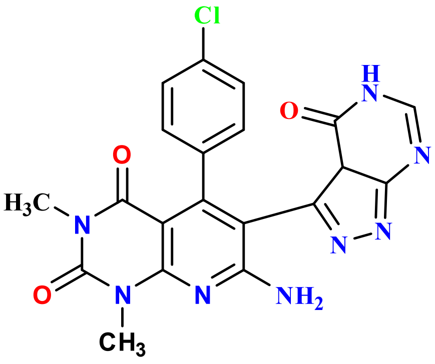

11

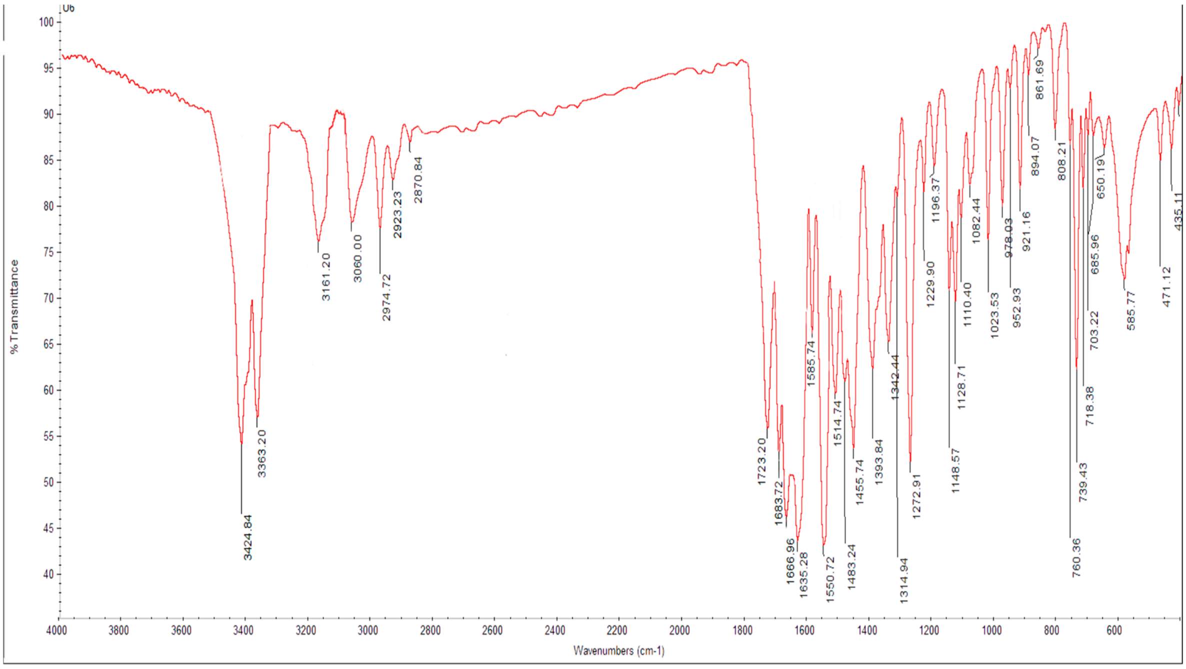

Figure S34 . IR of Compound **11**

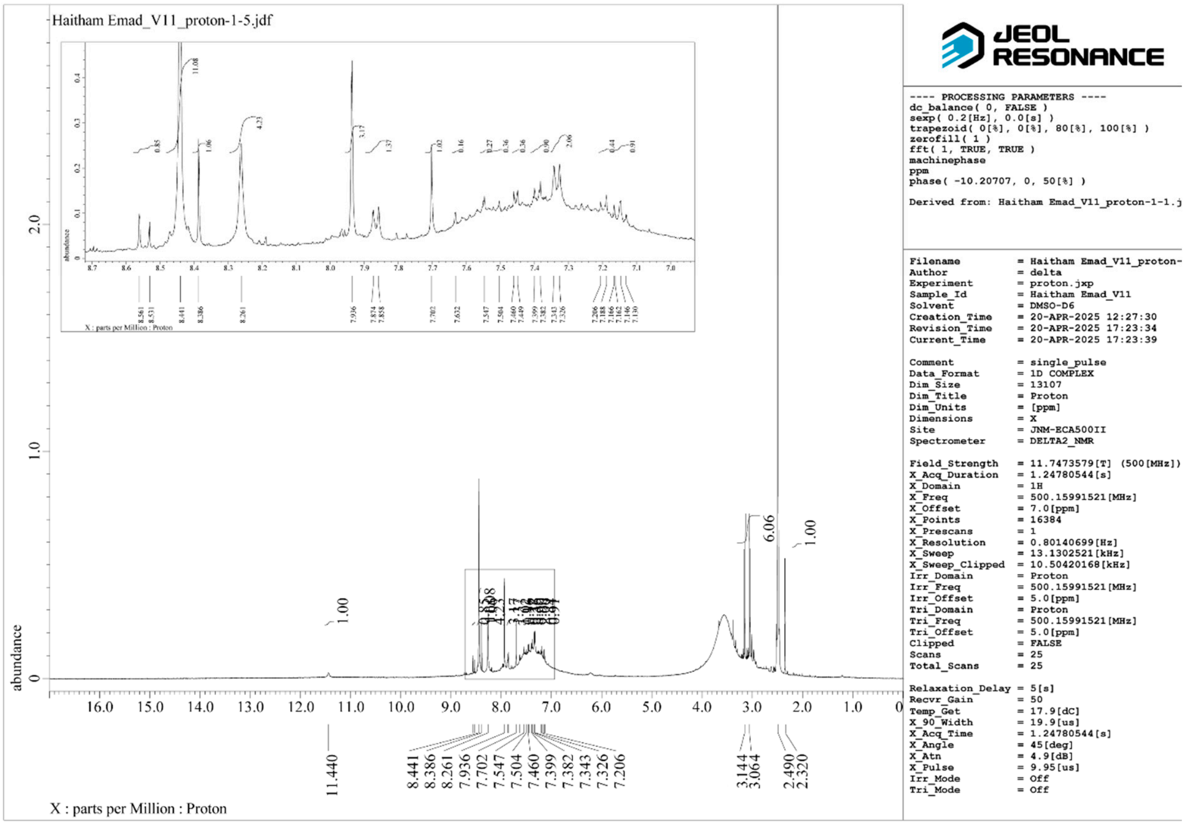

Figure S35. ^{1}H NMR spectrum (400 MHz, DMSO) of compound **11**

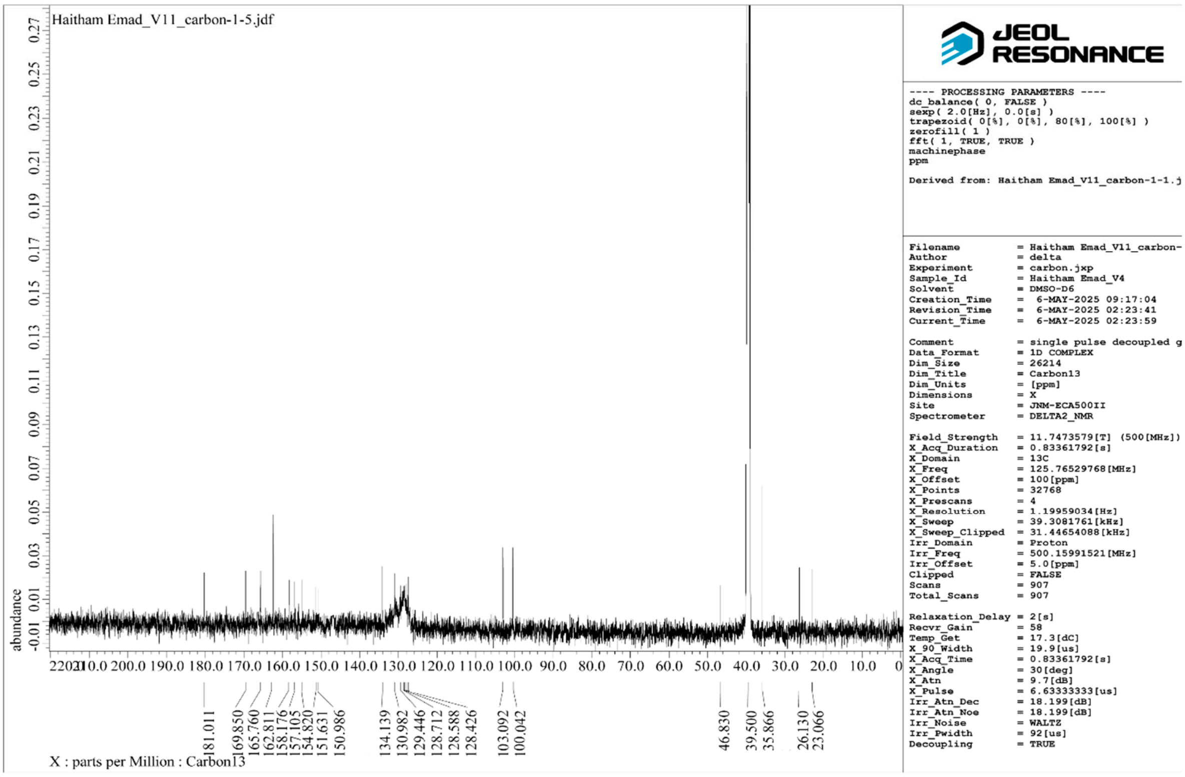

Figure S36. ^{13}C NMR spectrum (100 MHz, DMSO) of compound **11**

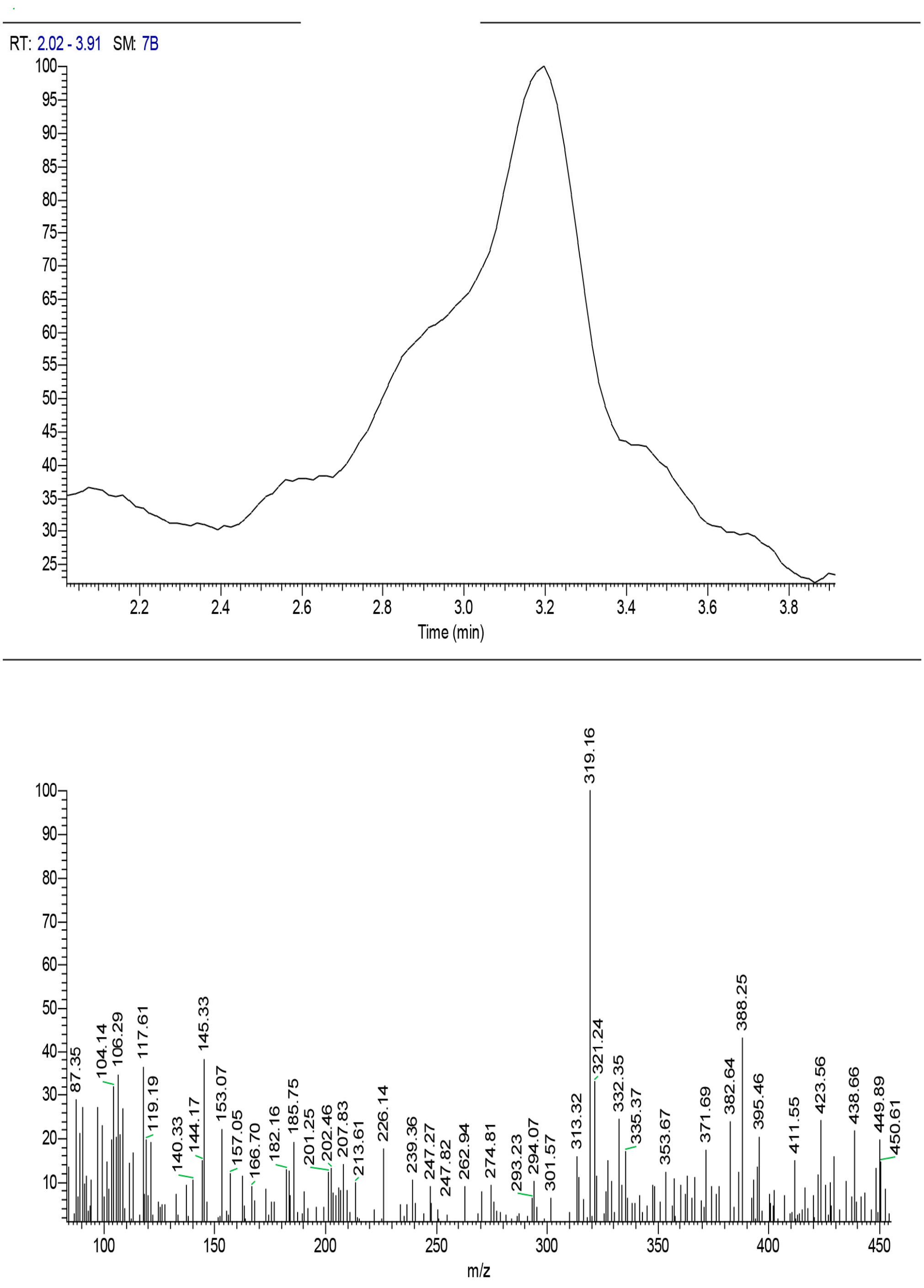

Figure S37. Mass spectrum of **11**

Characterization of Compound 12:-

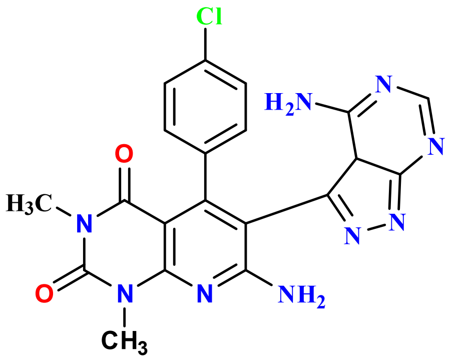

12

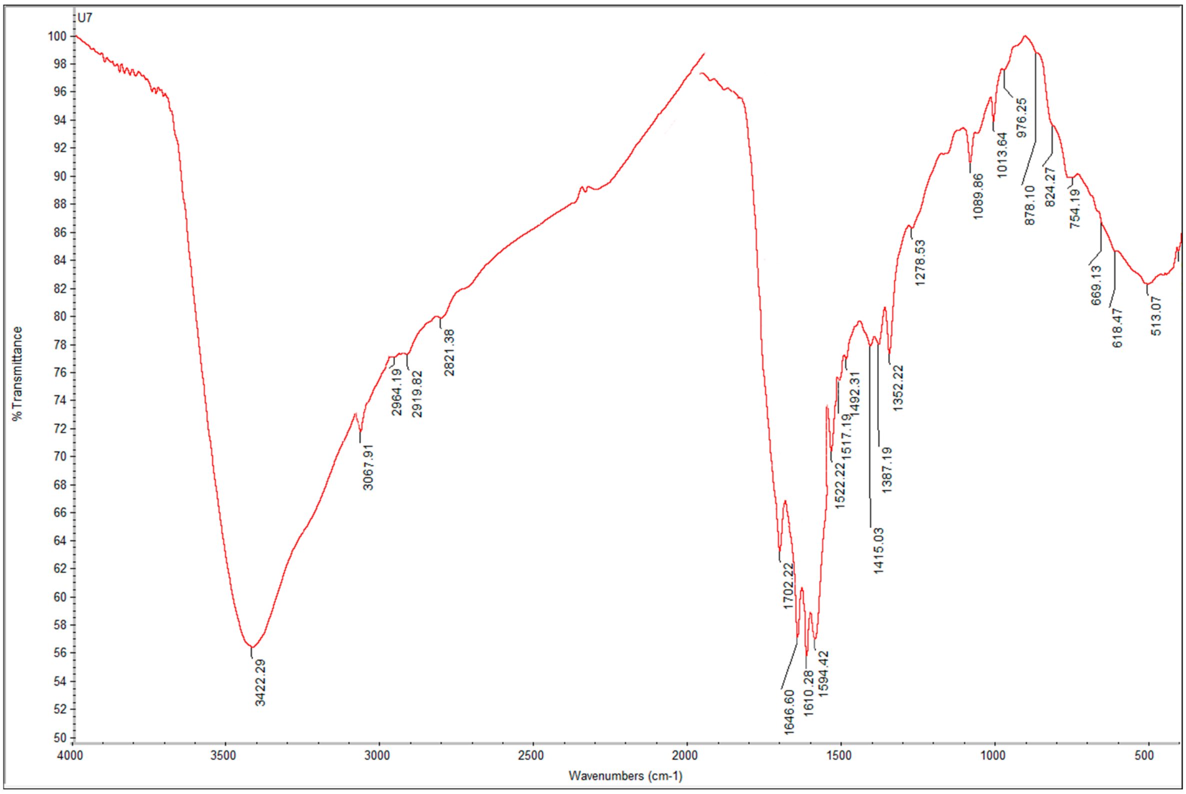

Figure S38 . IR of Compound **12**

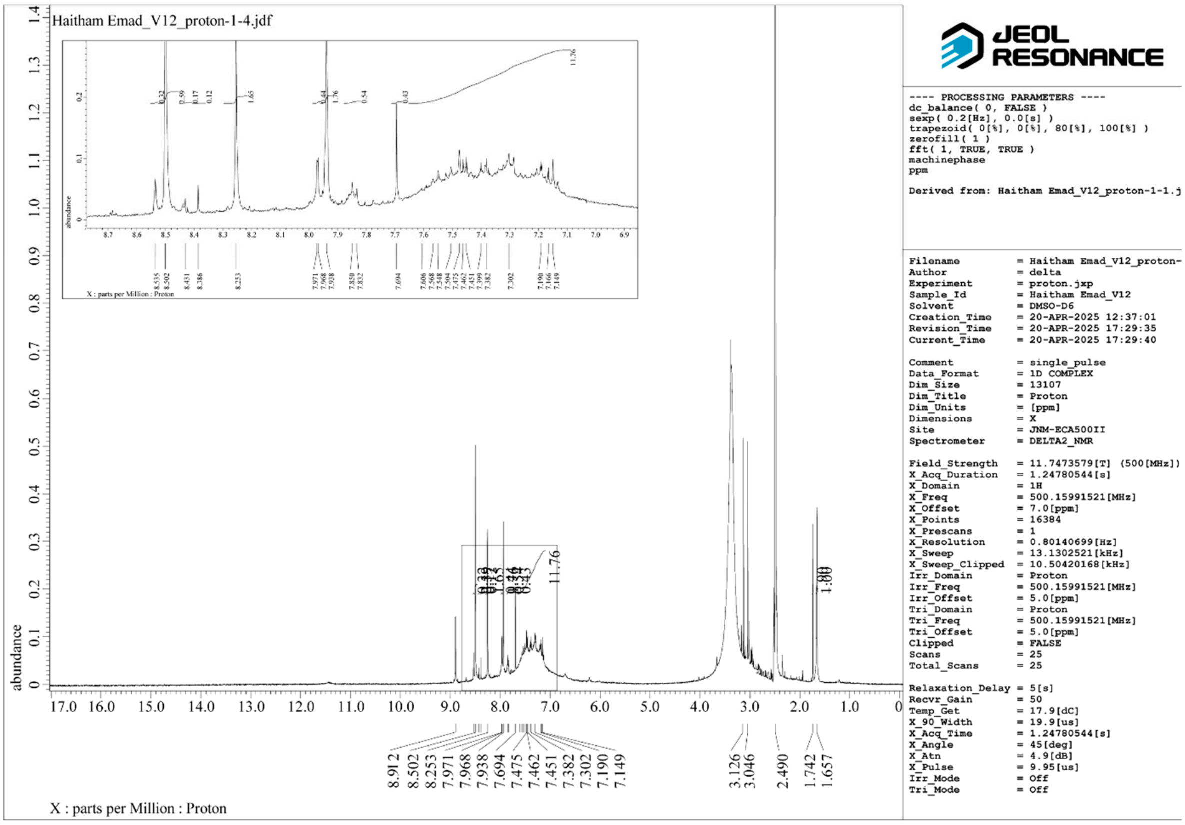

Figure S39. ^{1}H NMR spectrum (400 MHz, DMSO) of compound **12**

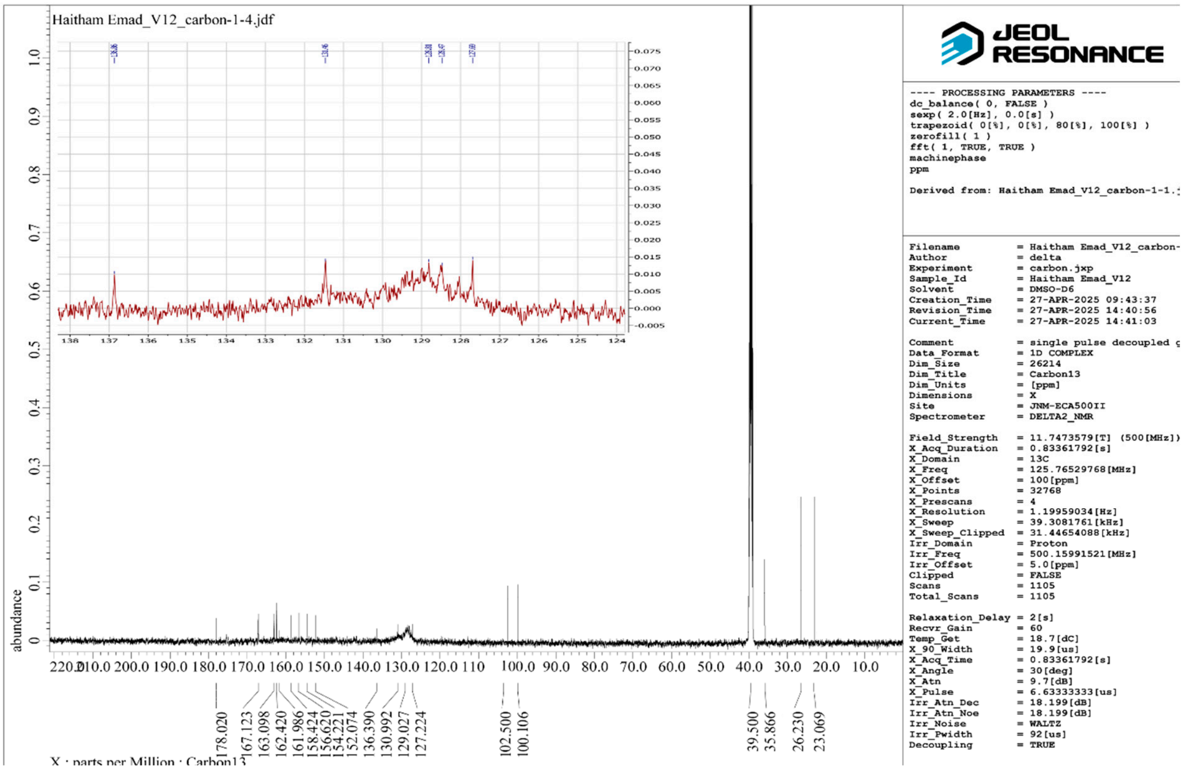

Figure S40. ^{13}C NMR spectrum (100 MHz, DMSO) of compound **12**

Characterization of Compound 13:-

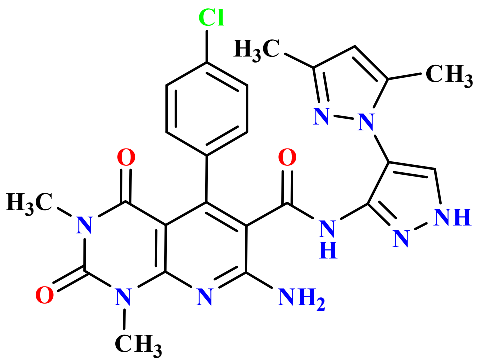

13

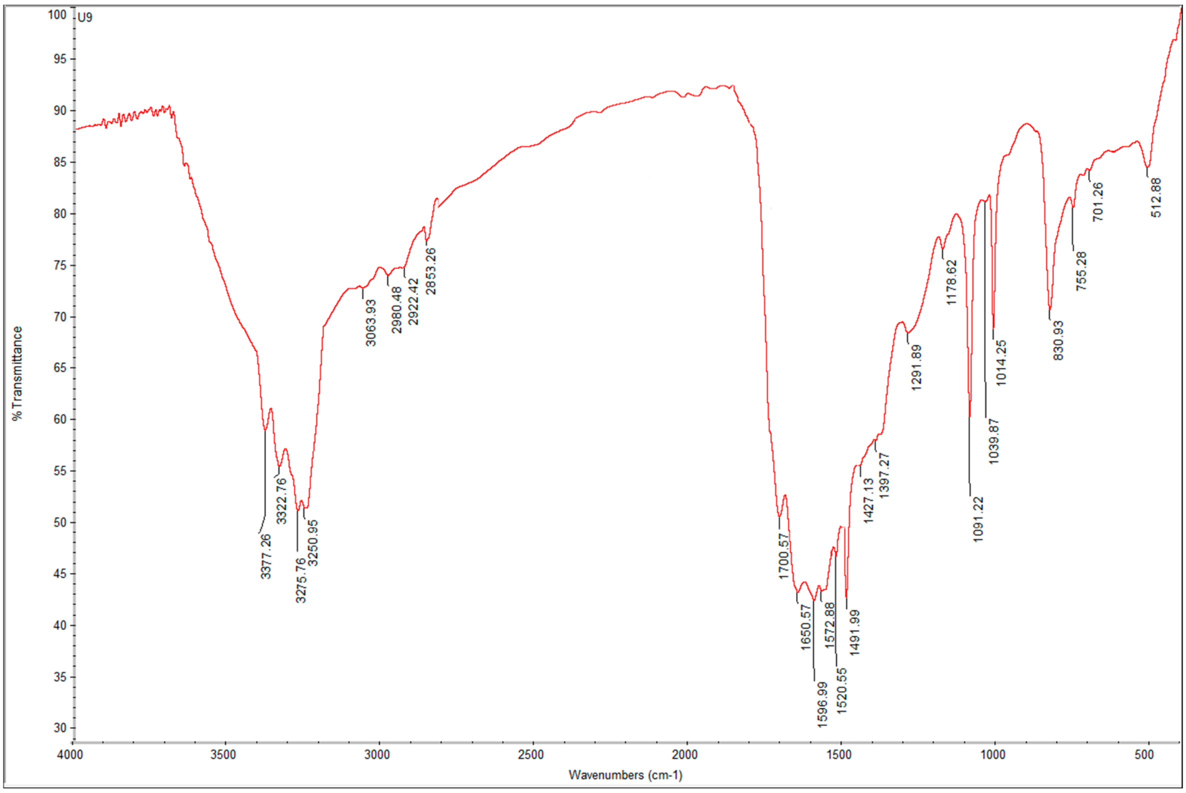

Figure S41 . IR of Compound **13**

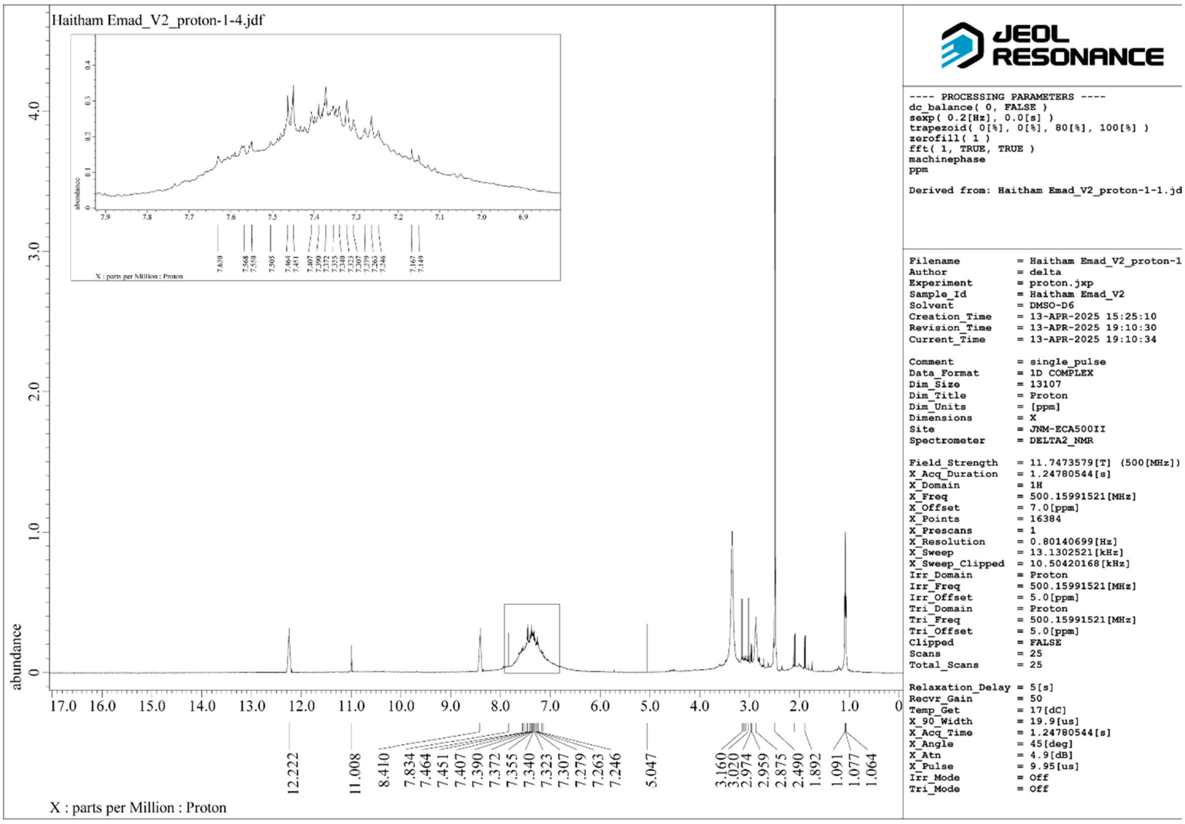

Figure S42. ^{1}H NMR spectrum (400 MHz, DMSO) of compound **13**

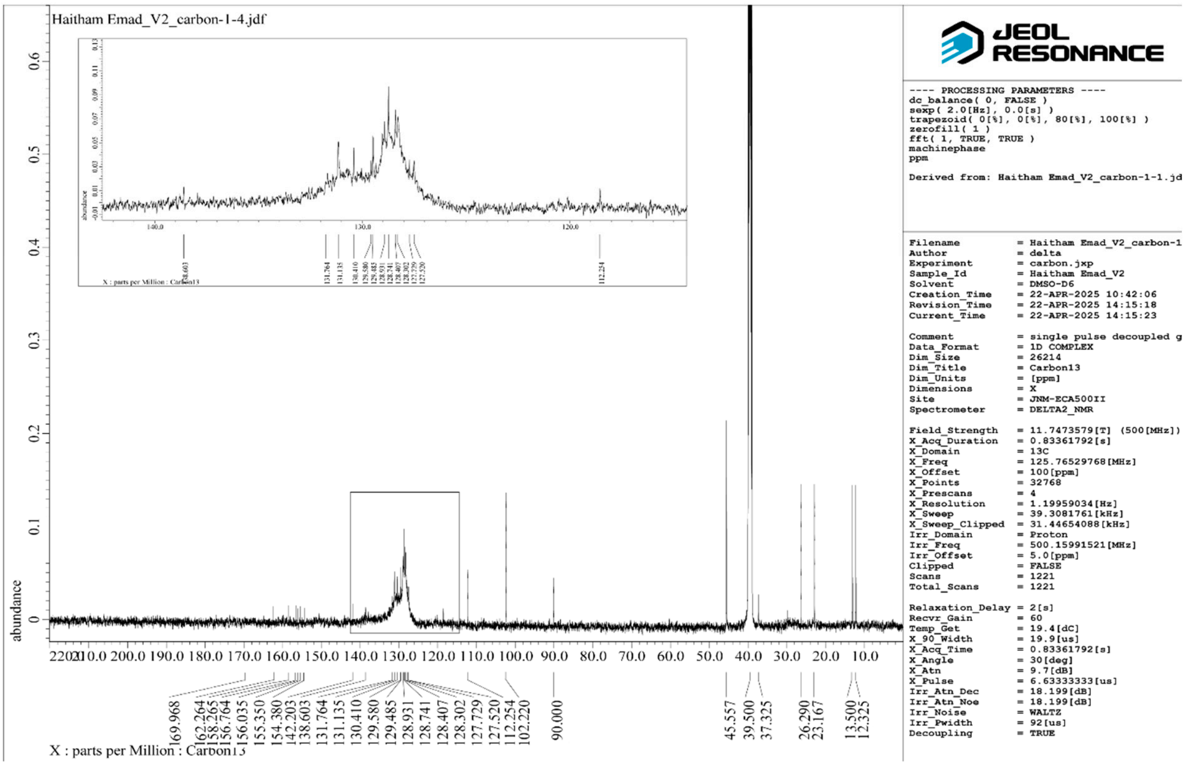

Figure S43. ^{13}C NMR spectrum (100 MHz, DMSO) of compound **13**

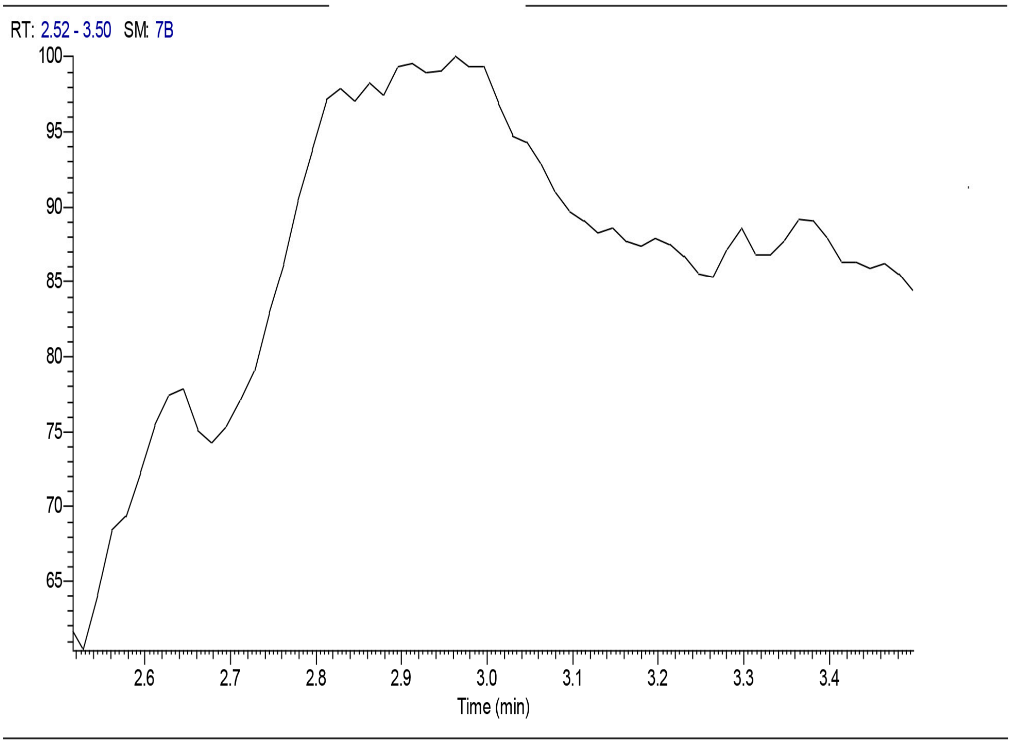

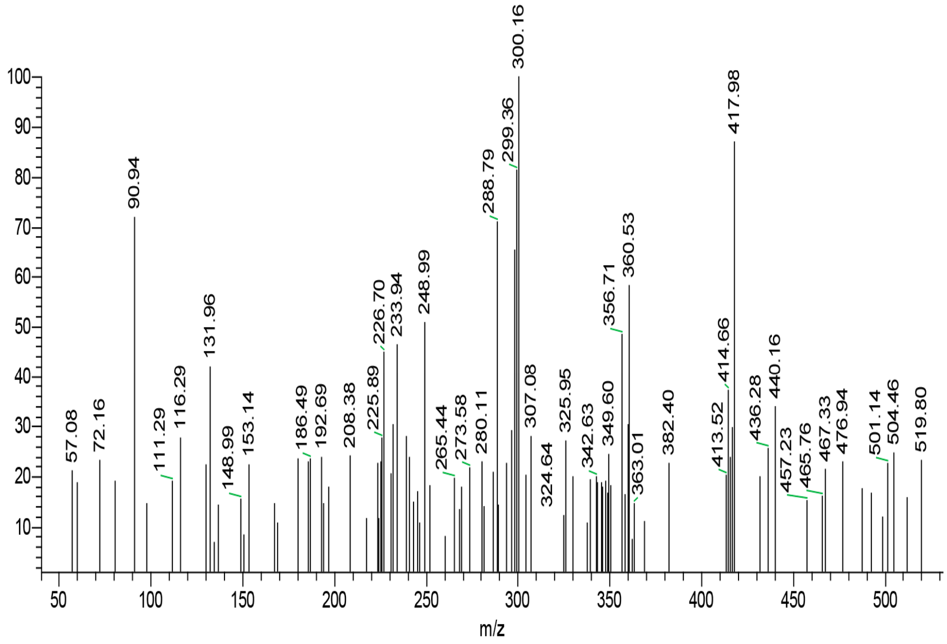

Figure S44. Mass spectrum of **13**

Characterization of Compound 14:-

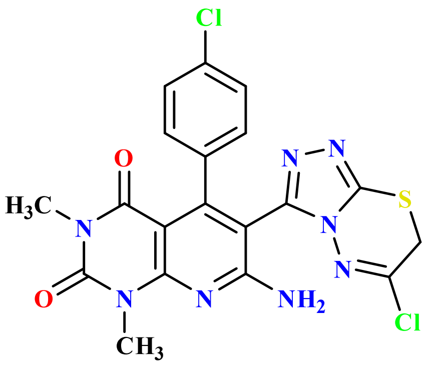

14

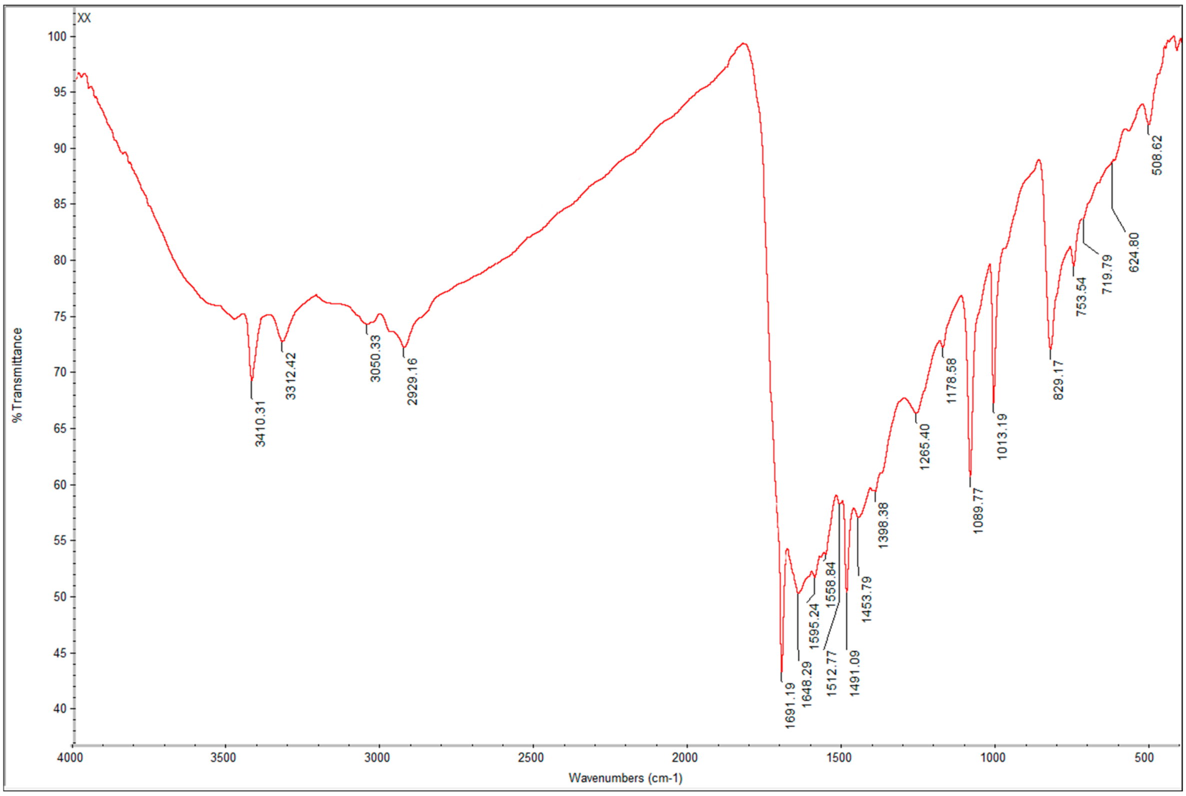

Figure S45 . IR of Compound **14**

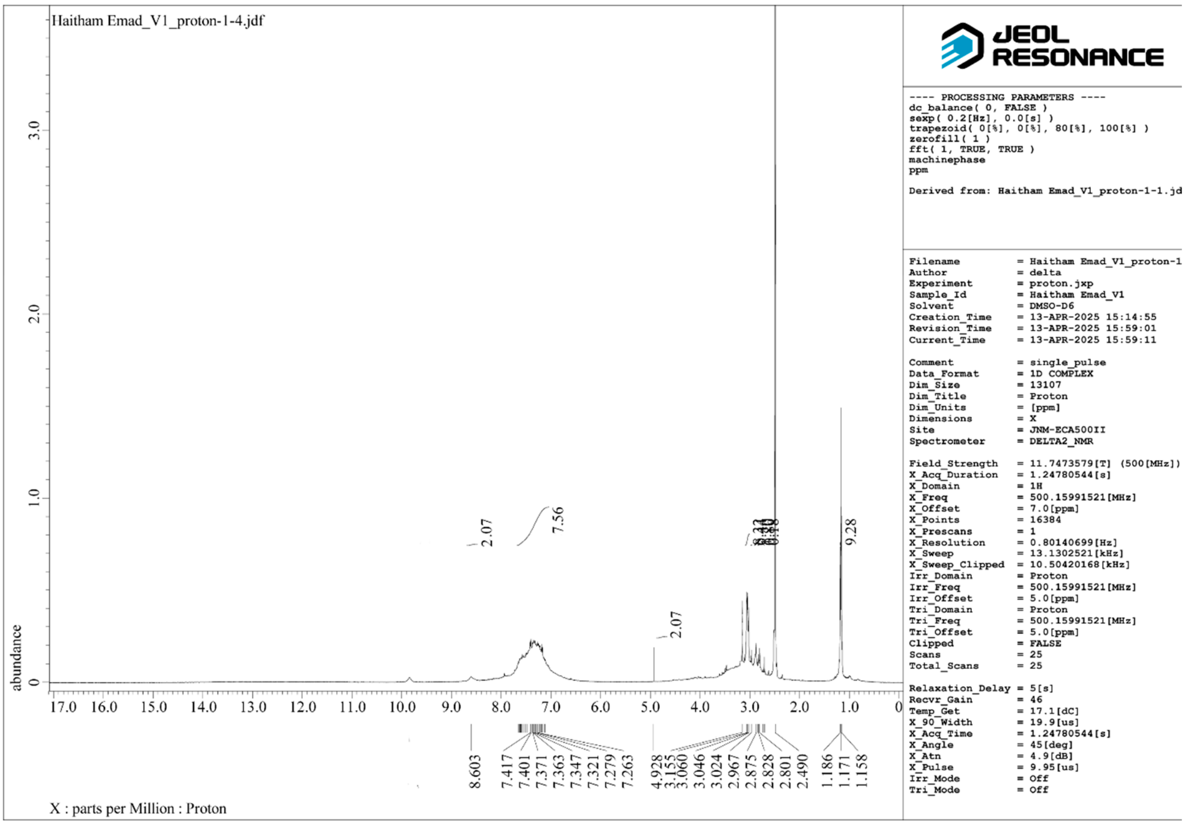

Figure S46. ^{1}H NMR spectrum (400 MHz, DMSO) of compound **14**

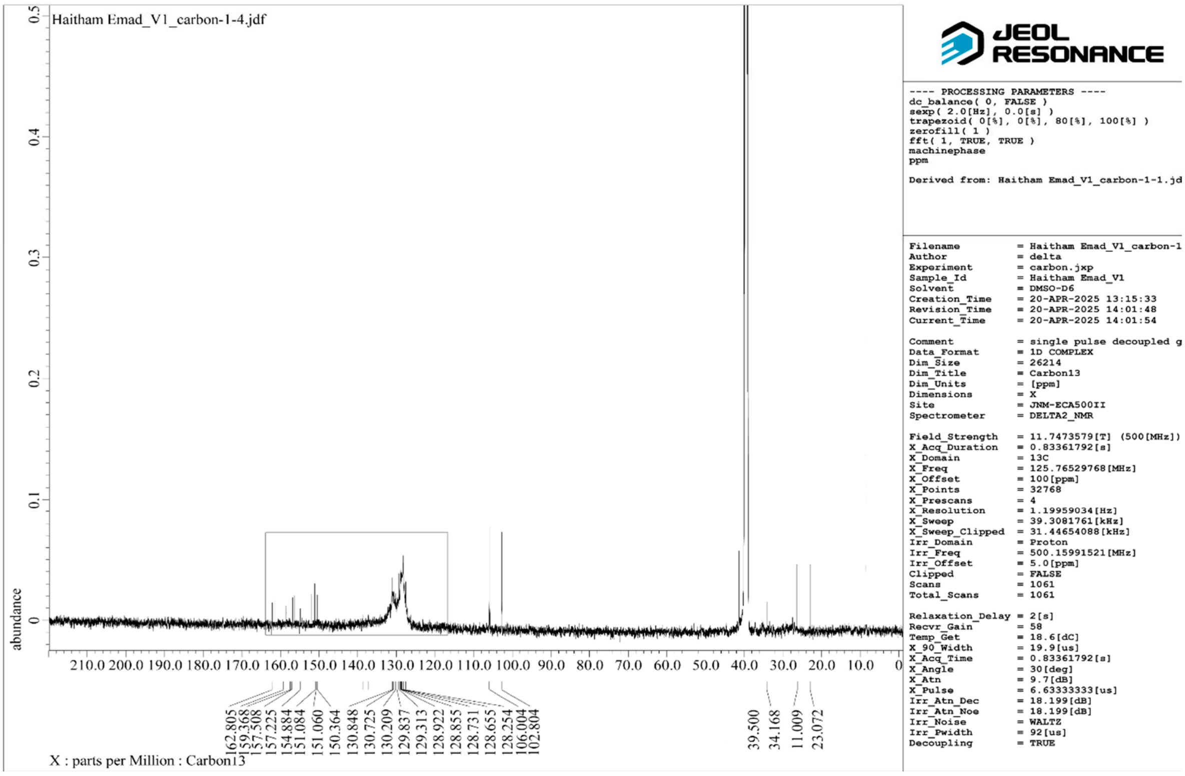

Figure S47. ^{13}C NMR spectrum (100 MHz, DMSO) of compound **14**

Characterization of Compound 15:-

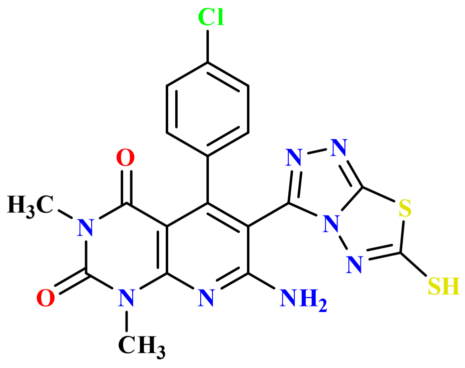

15

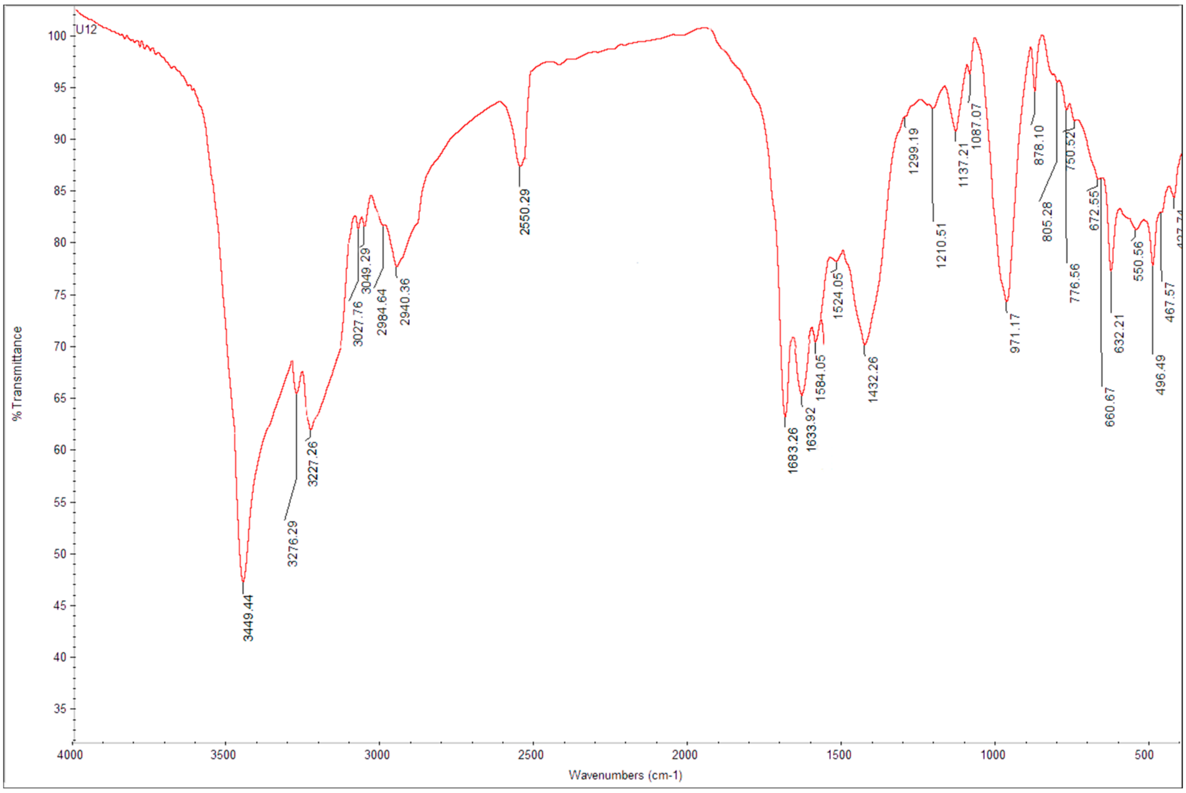

Figure S48 . IR of Compound **15**

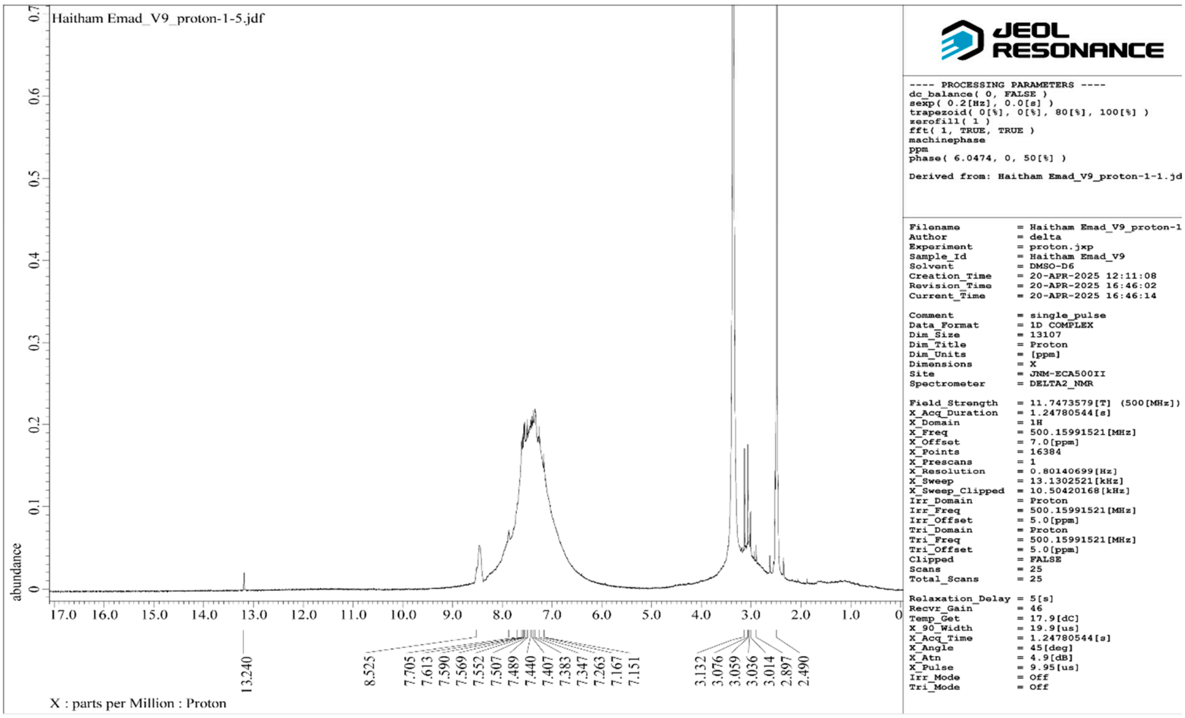

Figure S49. ^{1}H NMR spectrum (400 MHz, DMSO) of compound **15**

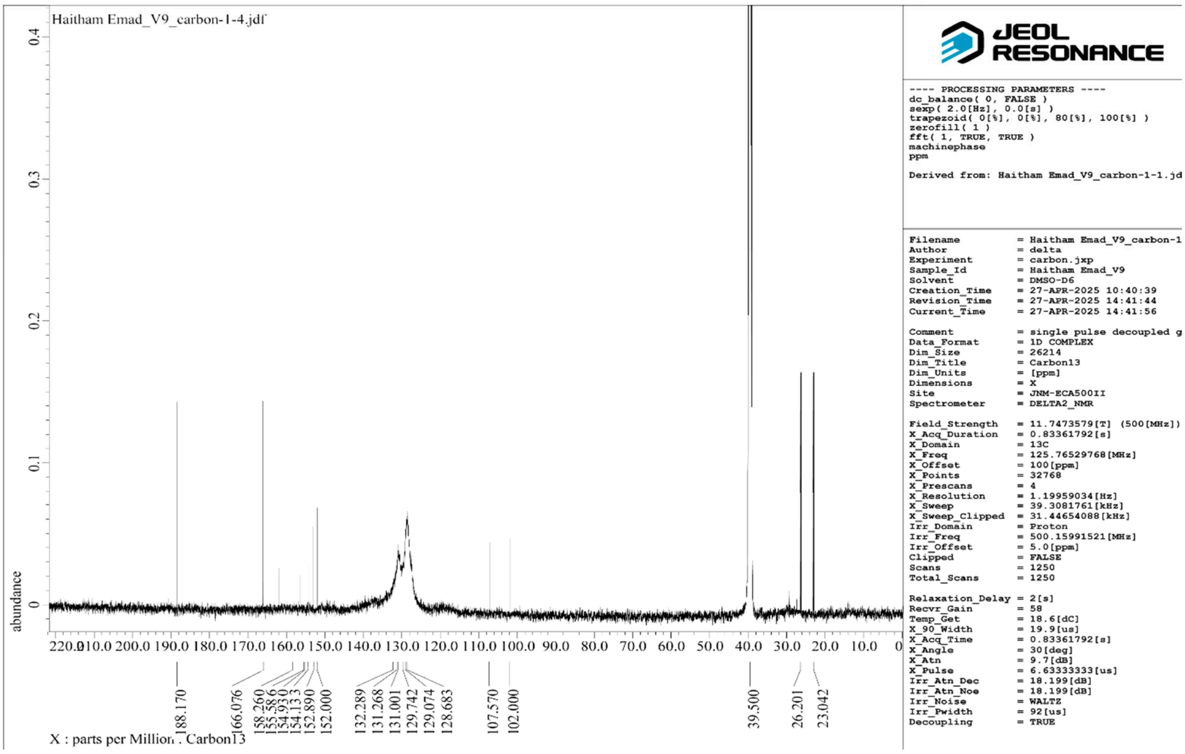

Figure S50. ^{13}C NMR spectrum (100 MHz, DMSO) of compound **15**

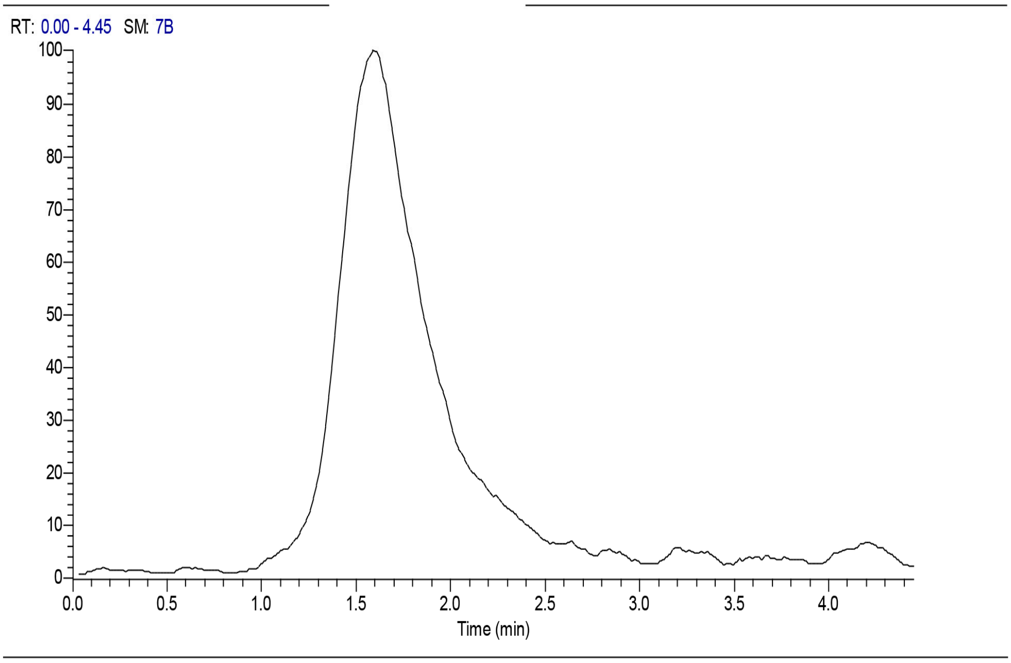

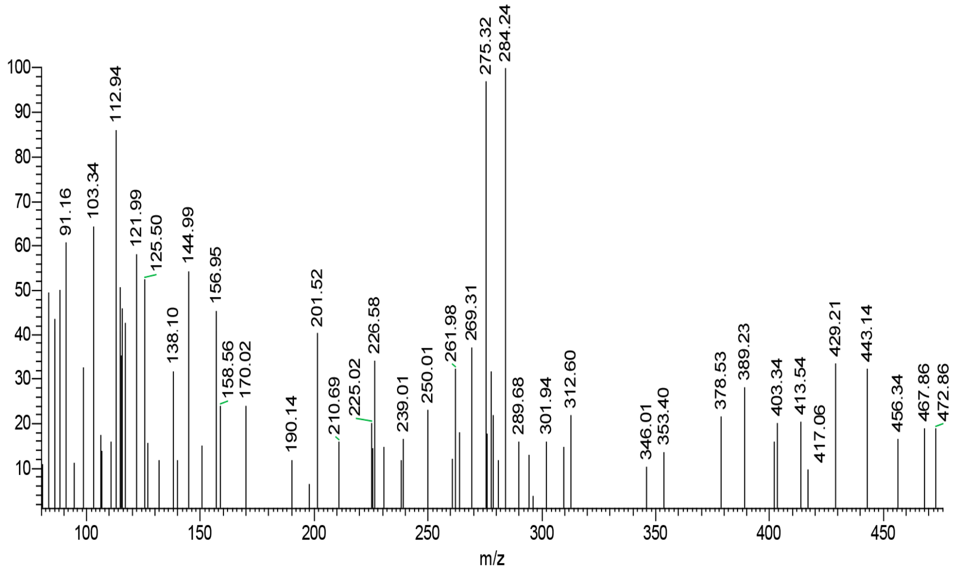

Figure S51. Mass spectrum of **15**

Characterization of Compound 16:-

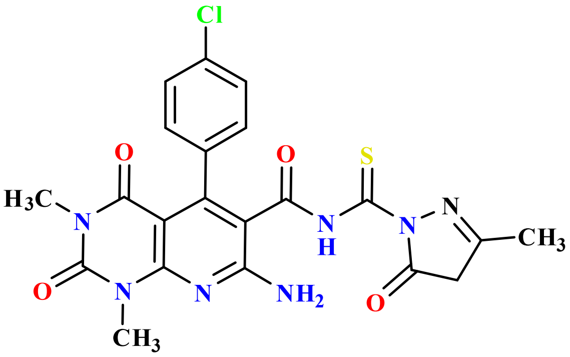

16

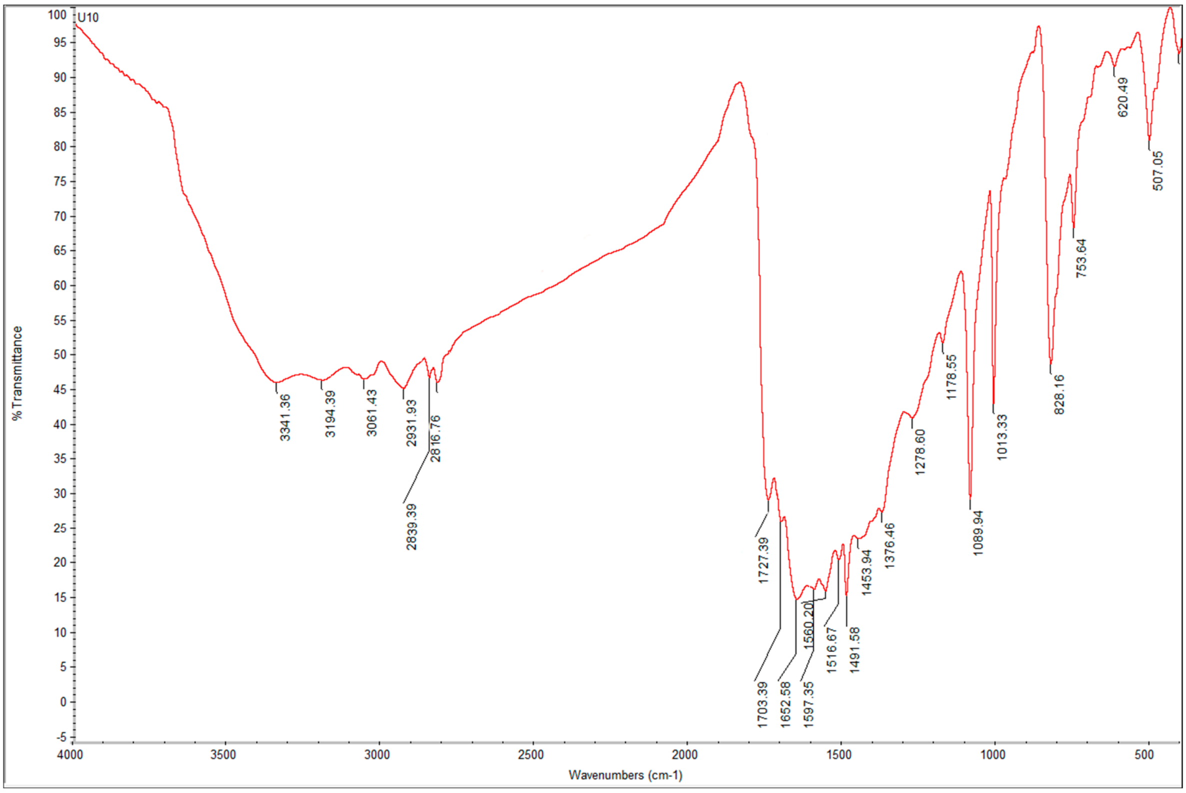

Figure S52 . IR of Compound **16**

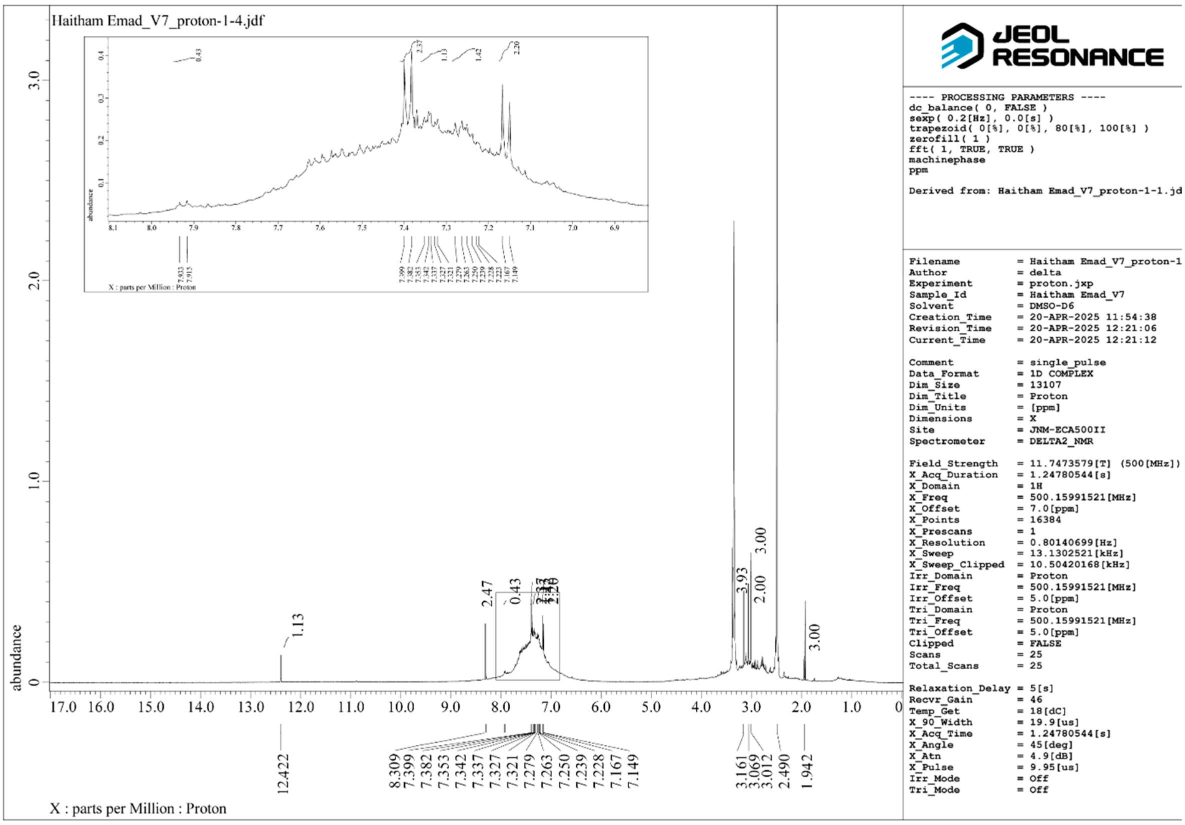

Figure S53. ^{1}H NMR spectrum (400 MHz, DMSO) of compound **16**

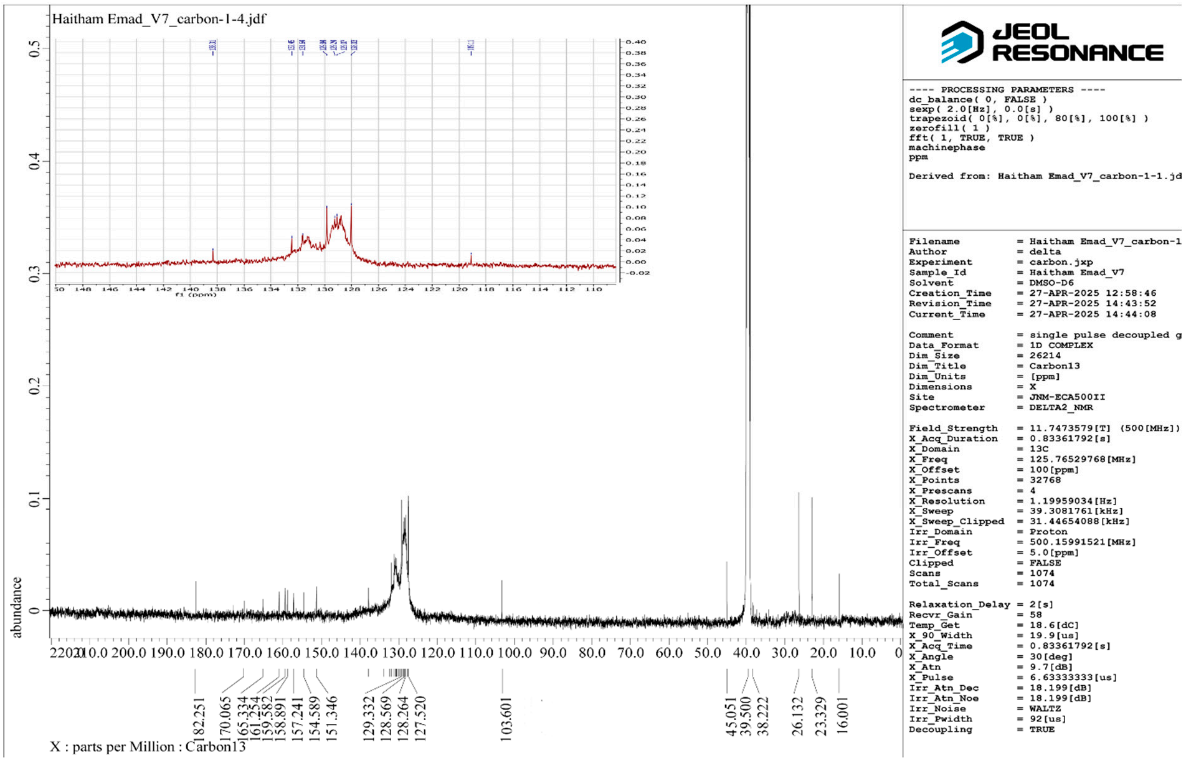

Figure S54. ^{13}C NMR spectrum (100 MHz, DMSO) of compound **16**

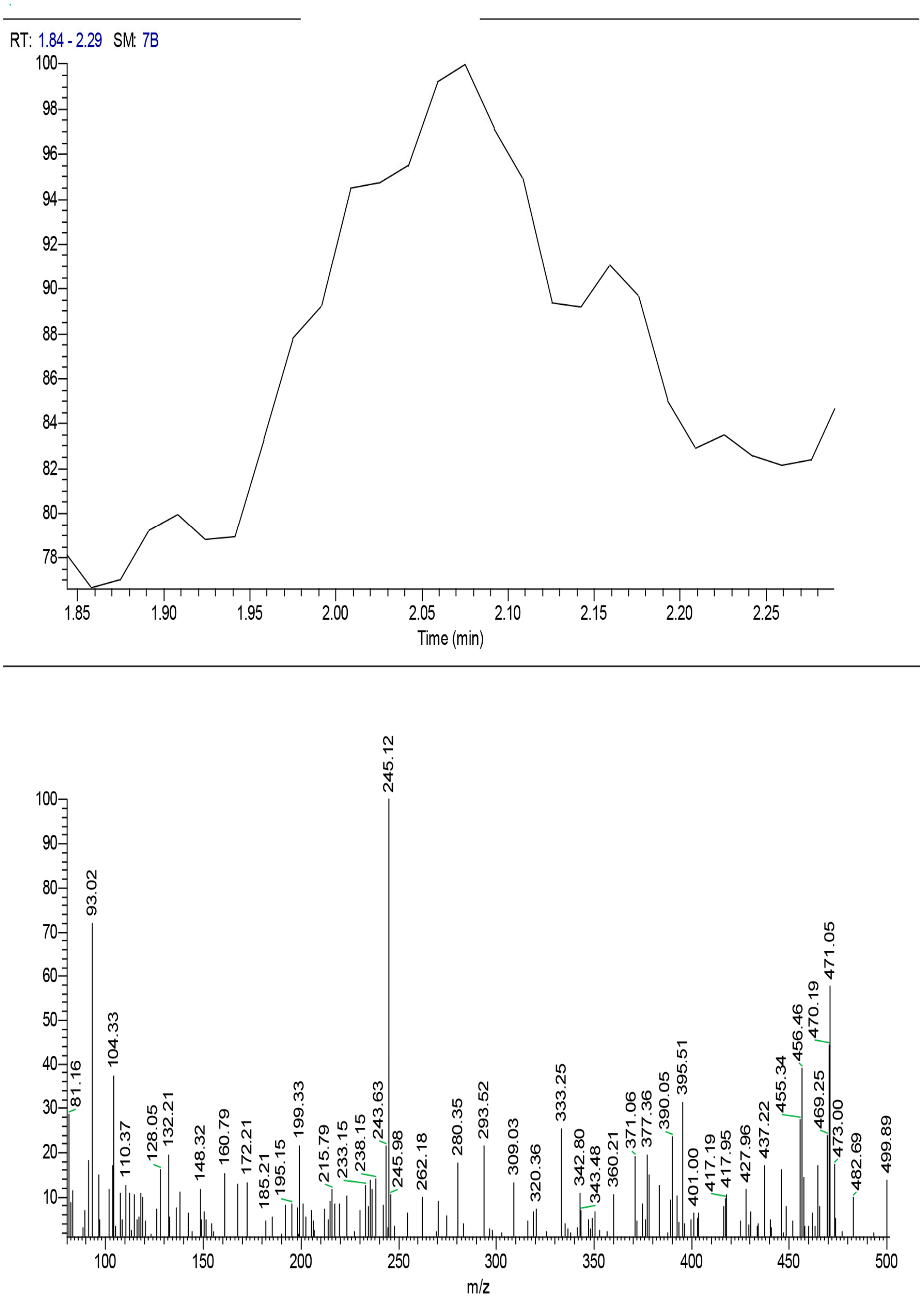

Figure S55. Mass spectrum of **16**

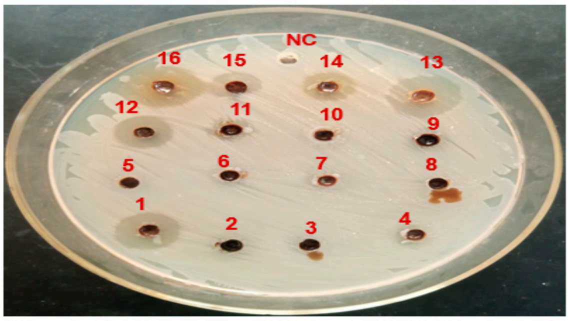

Escherichia coli

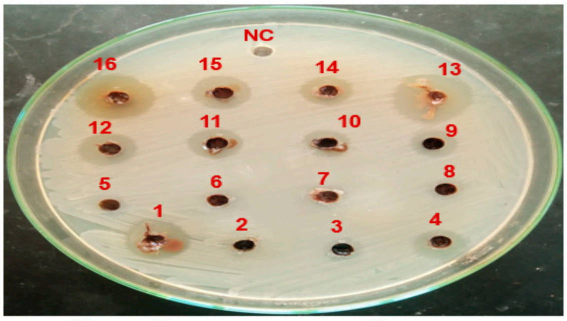

Pseudomonas aeruginosa

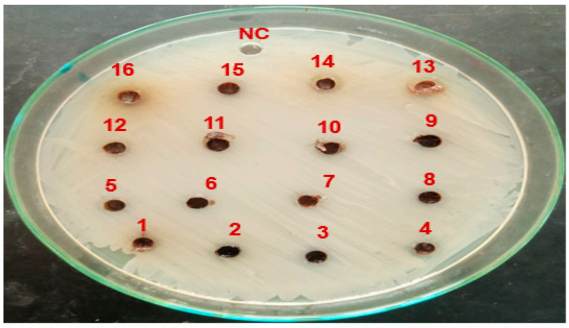

Staphylococcus aureus

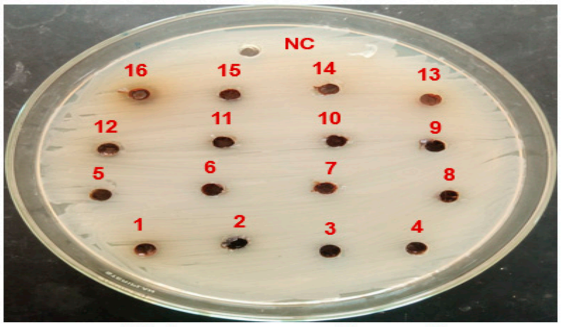

Enterococcus faecalis

Figure S56. Antimicrobial activity of samples evaluated by well diffusion method against *S. aureus* ATCC25923, *E. faecalis* ATCC29212 *P. aeruginosa* ATCC10145,and *E. coli* ATCC25915
